# Differences between the elimination of early and late transition metals: DFT mechanistic insights into the titanium-catalyzed synthesis of pyrroles from alkynes and diazenes†
†Electronic supplementary information (ESI) available: Additional computational results, energies, and Cartesian coordinates of the optimized structures. See DOI: 10.1039/c6sc04456e



**DOI:** 10.1039/c6sc04456e

**Published:** 2016-12-22

**Authors:** Jiandong Guo, Xi Deng, Chunyu Song, Yu Lu, Shuanglin Qu, Yanfeng Dang, Zhi-Xiang Wang

**Affiliations:** a School of Chemistry and Chemical Engineering , University of the Chinese Academy of Sciences , Beijing 100049 , China . Email: zxwang@ucas.ac.cn; b Collaborative Innovation Center of Chemical Science and Engineering , Tianjin 300072 , China

## Abstract

Early transition metals (TMs), such as titanium, generally resist undergoing reductive elimination to form C–X bonds due to their weak electronegativity. By analyzing the mechanism of the titanium-catalyzed synthesis of pyrroles from alkynes and diazenes, the present study revealed that titanium is able to promote C–N bond formation *via* an unconventional elimination pathway, passing through a comparatively stable masked Ti^II^ complex (*i.e.*, **IM4**) rather than pyrrole directly. The formation of **IM4** originates from the bilateral donation and back-donation between Ti and the pyrrole ligand. Formally, it could be considered that the two electrons resulting from the unconventional reductive elimination are temporarily buffered by back-donation to a symmetry-allowed unoccupied π-orbital of the pyrrole ring in **IM4** rather than becoming a lone pair on a Ti center as adopted in the catalysis of late TMs. Because of its stability, **IM4** requires additional oxidation by diazene to liberate pyrrole. The triplet counterpart (**IM4^T^**) of **IM4** is more stable than **IM4**, but the elimination is unlikely to reach **IM4^T^**, because the process is spin-forbidden and the spin–orbit coupling is weak. Alternatively, one may consider the forming pyrrole in **IM4** as a redox-active ligand, reserving the two electrons resulting from the formal reductive elimination and then releasing the electrons when **IM4** is oxidized by diazene. These insights allow us to propose the conditions for early TMs to undergo a similar elimination, whereby the forming product will have symmetry-allowed frontier molecular orbitals to form donation and back-donation bonding with a TM center and a substrate possessing a comparatively strong oxidizing ability to oxidize an **IM4**-like intermediate for product release. These insights may provide another way of constructing C–X bonds through a similar reductive elimination pathway, using early TM catalysts.

## Abstract 2

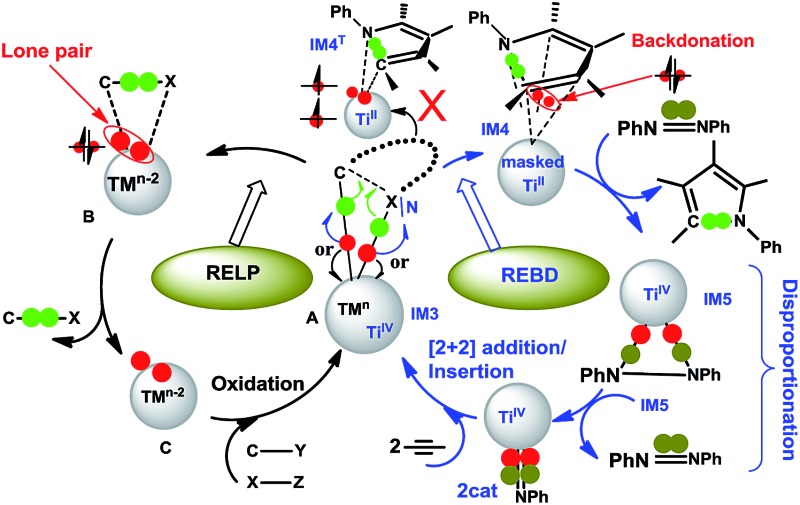
A DFT study demonstrates that titanium is capable of promoting C–N bond formation *via* an unconventional reductive elimination pathway featuring back-donation (REBD).

## 1. Introduction

**Scheme 1:**
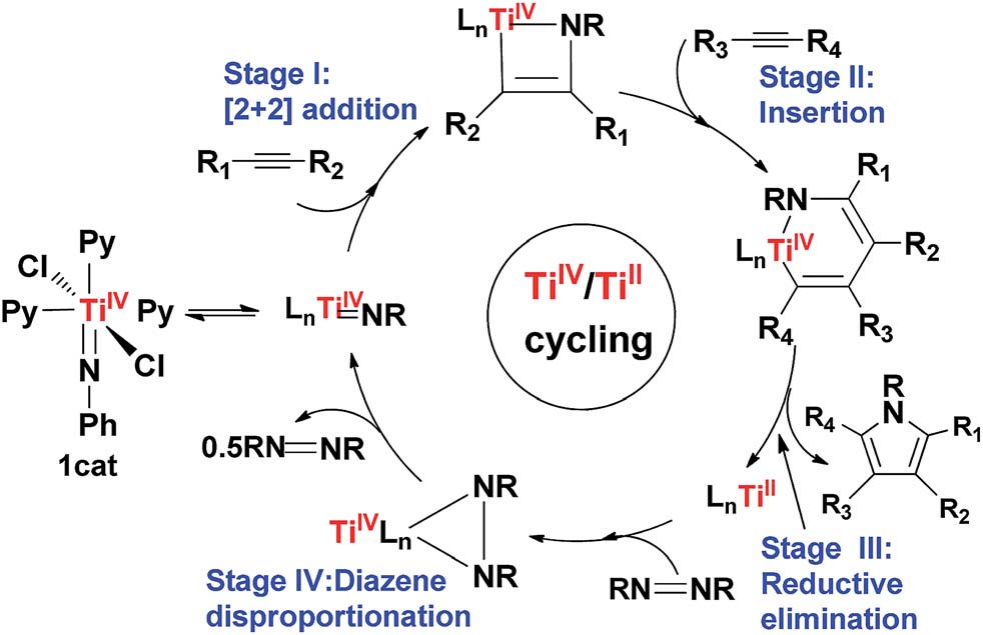
Proposed catalytic cycle by Tonks *et al.*

Titanium is a desirable transition metal (TM) for developing green catalytic transformations because of its abundance and non-toxicity.^1^ Among others, recent developments in titanium catalysis have been dominated by transformations involving hydrofunctionalization.^1,2^ Compared to catalysis with late TMs, such as Ru, Rh, Pd, and Ir, the variety of titanium catalysis is limited, which is commonly attributed to titanium's strong resistance to undergo redox cycling (*e.g.*, Ti^IV^/Ti^II^) because of its weak electronegativity, and thus efforts have been devoted to develop redox catalysis with early TMs using redox-active ligands.^3^ Nevertheless, Odom *et al.* ^1*a*^ prospected that “*this relative lack of diversity is largely due to the need for further development rather than an inherent lack of utility*”. Indeed, Tonks *et al.* ^4^ recently accomplished pyrrole synthesis from alkynes and diazenes (*e.g.*, eqn (1)), catalyzed by a titanium imido complex **1cat**.^5^ Intriguingly, these transformations were able to form new C–N bonds without using a redox-active ligand. It was proposed that the C–N bond formation could take place *via* a reductive elimination (Scheme 1). While reductive elimination is a ubiquitous elementary step in the late TM catalysis to form C–X bonds,^6–9^ it is rare in the early TM catalysis. Inspired by the remarkable experimental advances, we applied density functional theory (DFT) computations to gain an insight into the mechanism of the transformations, with an aim to unveil the mechanistic differences in the eliminations in the catalysis of early and late TMs. Interestingly, the study characterized that the C–N bond formation proceeds *via* a formal reductive elimination pathway involving donation and back-donation. Formally, one could consider that the two electrons resulting from the formal reductive elimination back-donate to a symmetry-allowed unoccupied molecular orbital of the pyrrole ring. This is different from the conventional reductive elimination in the catalysis of late TMs, where the two electrons resulting from the elimination become a lone pair located at the TM center.
1

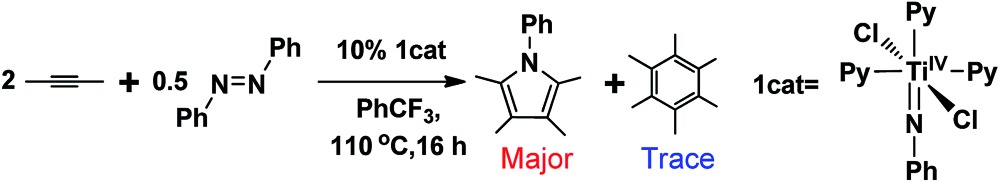




## 2. Computational methods

Using actual catalyst and substrates in eqn 1, all the geometries were optimized and characterized by frequency analysis calculations to be minima (without imaginary frequency) or transition states (having unique imaginary frequency) at the B3LYP/6-31G(d,p) level in the gas phase. With the optimized geometries, the energies were further refined by M06-L^10^/6-311++G(d,p) single-point energy calculations with solvent effects accounted for by the PCM solvent model in the experimentally used solvent (PhCF_3_). The refined energies were finally corrected to enthalpies and free energies at 298.15 K and 1 atm. using the gas phase B3LYP/6-31G(d,p) frequencies; only the free energies are discussed. The Wiberg bond indices (WBIs),^11^ natural bond orbital (NBO) charges,^12^ and nuclear independent chemical shift values (NICS)^13^ were calculated at the M06-L(PCM, solvent = PhCF_3_)/6-311++G(d,p)/B3LYP/6-31G(d,p) level. All the standard DFT calculations were performed using the Gaussian 09 program.^14^ Minimum energy crossing points (MECPs) were obtained using the MECP-location program developed by Harvey's group.^15^ The spin–orbit coupling (SOC) effects at the MECPs were evaluated using the MOLPRO program.^16^

## 3. Results and discussion

### 3.1 Energetic feasibility of the transformations

**Fig. 1:**
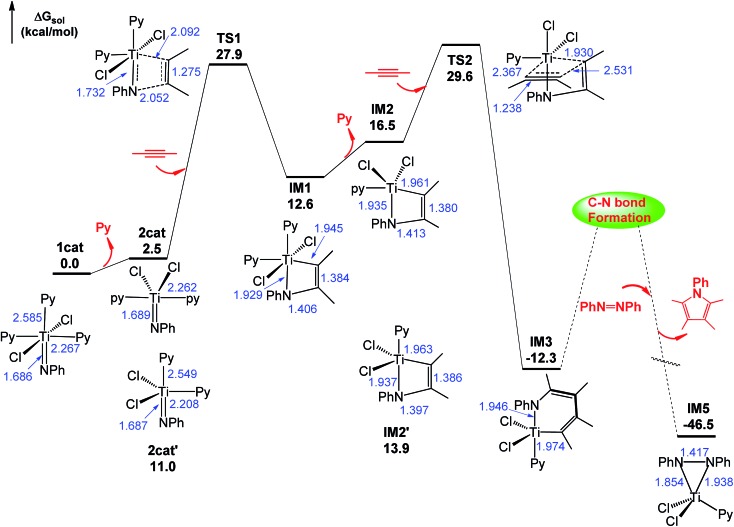
Free energy profile for stages I–III of the catalytic cycle. Optimized structures of the stationary points are displayed in Fig. S1.† The values in blue are the key bond distances in angstroms. The mechanism for C–N bond formation will be discussed in Section 3.2.

Using eqn (1) as a representative, we first examined the energetic feasibility to complete the catalytic cycle, as shown in Scheme 1. Fig. 1 illustrates the most favorable pathway from stage I to stage III of the cycle we explored. To undergo [2 + 2] addition (stage I), which often takes place in the alkyne hydroaminations catalyzed by early TM = NR imido complexes,^2a–k^ the coordinatively saturated 16e octahedral titanium complex **1cat** first releases a pyridine (Py) ligand, enabling the Ti center to interact with an alkyne substrate. In agreement with the experimental observation,^17^ the liberation of an axial Py ligand (Py^ax^) giving **2cat** is 8.5 kcal mol^–1^ more favorable than the release of an equatorial Py ligand (Py^eq^) giving **2cat′** due to the strong *trans* effect of the imido group on Py^ax^, as reflected by the longer Ti–N(Py) bond length (2.549 Å) in **2cat′** than that (2.262 Å) in **2cat** (Fig. 1). Note that the barrier for convertting **2cat′** to **2cat** *via* swinging the axial Py ligand to the equatorial position is low (4.3 kcal mol^–1^). Upon the active species being accessible, alkyne adds to the imido Ti=N bond. Taking both **2cat** and **2cat′** into account, we located the [2 + 2] addition transition states (TSs), among which **TS1** was the lowest (Fig. S2†). Relative to **1cat**, the addition spans a barrier of 27.9 kcal mol^–1^ and is endergonic by 12.6 kcal mol^–1^.

Subsequent to the alkyne addition forming **IM1**, the reaction proceeds to alkyne insertion (stage II). Since **IM1** is coordinatively saturated, a Py ligand should be liberated, which gives **IM2** *via* releasing a Py^ax^ or **IM2′** *via* releasing a Py^eq^. The insertions of an alkyne into the Ti–C and Ti–N bonds of **IM2** and **IM2′** were considered (Fig. S3†). **TS2** *via* alkyne interaction with the π orbitals of **IM2** is 4.2 kcal mol^–1^ lower than its TS counterpart of **IM2′**. Relative to **IM1**, the insertion giving **IM3** overcomes a barrier of 17.0 kcal mol^–1^ and is exergonic by 24.9 kcal mol^–1^.

After alkyne insertion, **IM3** undergoes C–N bond formation (stage III), leading to a pyrrole product and intermediate **IM5**. The mechanism of this stage is the focus of the study and will be discussed separately in Section 3.2.

Intermediate **IM5** is not the active catalyst (**2cat** or **2cat′**). A key for the reaction running catalytically is the use of a disproportionation reaction to close the catalytic cycle (*i.e.*, stage IV in Scheme 1), converting **IM5** to the active catalyst **2cat** or **2cat′**. The stage breaks the N–N single bond in **IM5**, but the entire catalytic cycle essentially cleaves an azobenzene N=N double bond. The previous interest of others^18^ in cleaving the azobenzene N=N bond encouraged us to characterize this stage in detail. Tonks *et al.* explored the disproportionation mechanism and proposed two possible mechanisms.^4^ In agreement with their proposal, our energetic results rule out the mechanism occurring *via* passing a Ti^II^ complex formed by dissociating azobenzene from **IM5** (Fig. S4†). Hence, as shown below, we characterized an alternative mechanism *via* dimerization.

**Fig. 2:**
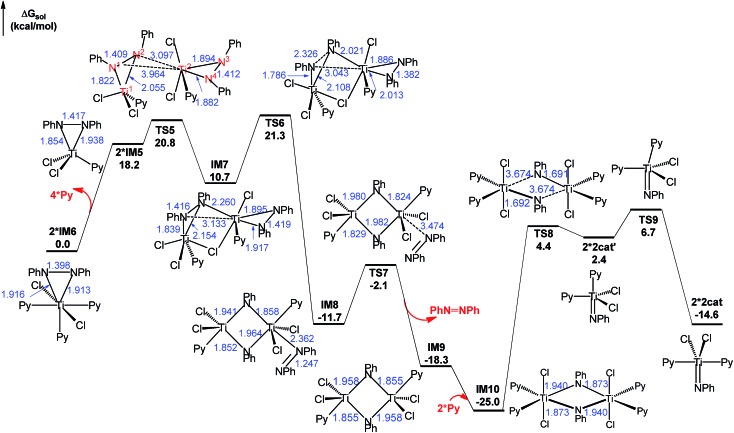
Free energy profile for regenerating the active catalysts *via* disproportionation, along with the optimized structures. The optimized structures of the stationary points are displayed in Fig. S5.† Key bond lengths in blue are given in angstroms.

Pyrrole product release from **IM3** gives **IM5** (Fig. 1). As shown in Fig. 2, on one hand, **IM5** associates with two Py ligands, giving **IM6**, with an energy release of 9.1 kcal mol^–1^. For the purpose of convenience, we used the more stable 2*IM6 as the energy reference to measure the energetics of the stage. On other hand, **IM5** can dimerize by crossing **TS5**, resulting in a weakly bound dimer **IM7**. The geometric parameters given in **IM7** indicate that it can be viewed as a doubly-bridged structure with one chlorine atom and one PhNNPh unit as the bridges. Subsequently, **IM7** climbs **TS6** to activate the N^1^–N^2^ bond. From **IM7** to **TS6**, the N^1^–N^2^ bond is significantly elongated from 1.416 to 2.326 Å, whereas the N^3^–N^4^ bond is shortened from 1.419 to 1.382 Å. Proceeding forward, a four-membered intermediate **IM8** is obtained, in which the N^1^–N^2^ bond is completely broken and a N^3^ =N^4^ double bond formed. The dissociation of azobenzene from **IM8** *via* **TS7** results in **IM9**. After associating with two Py ligands, **IM9** reaches the more stable **IM10**. The cleavage of two Ti–N bonds of the four-membered ring in **IM10** gives two **2cat′** species that can be easily isomerized to the more stable **2cat** *via* swinging the axial Py ligand to the equatorial position (**TS9**).

**Scheme 2:**
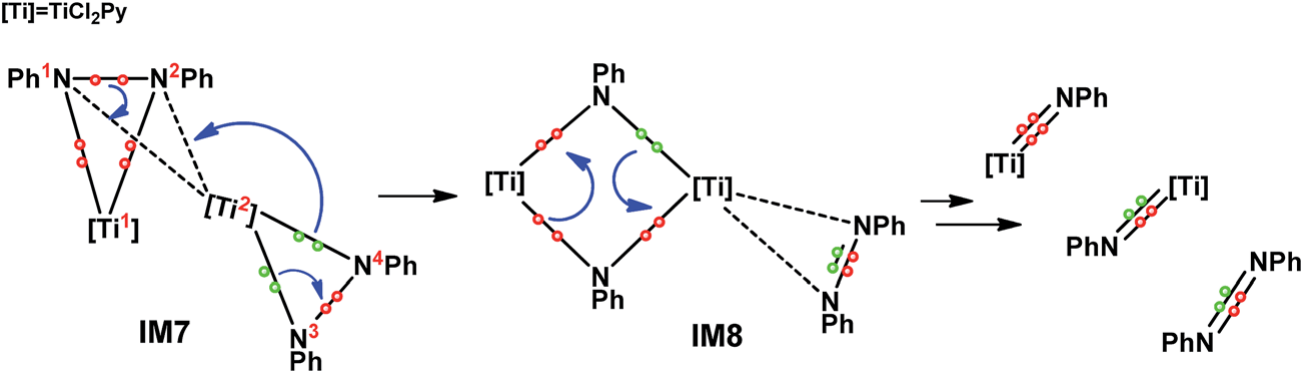
Mechanism for the bond formation and cleavage involved in **IM5** disproportionation, using electron-flow representation.

On the basis of the computed pathway, Scheme 2 simplifies our understanding of the disproportionation mechanism by tracing the electrons participating in bond formations and cleavages, using the electron-flow representation (Scheme 2). After forming the dimer **IM7**, among the two pairs of electrons in the two Ti–N bonds of the PhN^3^[Ti^2^]N^4^Ph unit, one pair flows to the N^3^–N^4^ bond, converting the single bond to a N=N double bond, and the remaining pair, together with the pair in the N^1^–N^2^ single bond, construct new Ti^2^–N^1^ and Ti^2^–N^2^ bonds between the two units, simultaneously breaking N^1^–N^2^ bond. These electron flows result in a four-membered (TiNTiN) intermediate **IM8**. By breaking two of the four Ti–N single bonds in **IM8**, two imido titanium complexes (the active species) are formed.

Examining the energetics of the pathway in Fig. 2, the barrier for the reaction to take place is 21.3 kcal mol^–1^ (**TS6** relative to 2*IM6), which is in agreement with the experimental fact that the disproportionation could take place rapidly at elevated temperature for a similar system (PhNNPh)TiCl_2_(NHMe_2_)_2_. However, though the conversion of 2*IM6 to 2*2cat is thermodynamically favorable by 14.6 kcal mol^–1^, the reaction could not lead to **2cat′** or **2cat** easily because of the high kinetic barrier (29.4 kcal mol^–1^) from **IM10** to **TS8** or 31.7 kcal mol^–1^ from **IM10** to **TS9**. Thus, experimentally, one could observe the occurrence of the reaction and most likely detect **IM10** but it could be difficult to detect **2cat** or **2cat′**. It is worth mentioning that numerous studies have reported X-ray structures featuring four-membered rings similar to that in **IM10** in azobenzene cleavage by metal complexes.^17^

Combining the energy profiles in Fig. 1 and 2, **IM10** is lowest in the catalytic cycle. Taking this into account, we estimated that the overall barrier to complete the catalytic cycle is 32.3 kcal mol^–1^, *i.e.*, the energy cost from **IM10** to **2cat** (5.2 kcal mol^–1^ = (25.0 – 14.6)/2, the division by 2 is due to that the disproportionation producing two **2cat** species) plus that from **2cat** to **TS2** (27.1 kcal mol^–1^, Fig. 1). This somewhat high barrier explains why these reactions needed to be performed at elevated temperatures (110.0 °C, eqn (1)).^4^ Briefly, our computed energetics demonstrates the feasibility of the catalytic cycle proposed by Tonks *et al.* ^4^ Furthermore, we gained an insight into the mechanism for the C–N bond formation stage.

### 3.2 Mechanism for the C–N bond formation stage

**Fig. 3:**
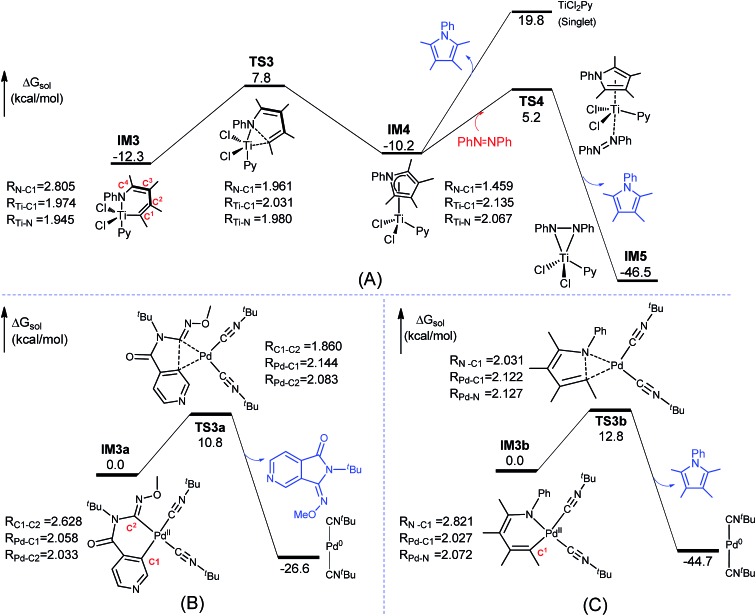
Comparisons of Ti- and Pd-eliminations. (A) Pathway for the C–N bond formation state omitted in Fig. 1(B) Pd-catalyzed C–C bond formation *via* reductive elimination. (C) Pd-catalyzed C–N bond formation *via* reductive elimination when replacing TiCl_2_Py in **IM3** with Pd(CN^*t*^Bu)_2_. Key bond lengths are given in angstroms. The optimized structures of all the stationary points are displayed in Fig. S7.†

The mechanism for the C–N bond formation stage omitted in Fig. 1 is detailed in Fig. 3A. The stage includes C–N bond formation from **IM3** to **IM4** and pyrrole release from **IM4** to **IM5**. Other alternative pathways leading from **IM4** to **IM5** were found to be less kinetically favorable than the **IM4**→**TS4**→**IM5** pathway (Fig. S6†). Fig. 3B shows the conventional reductive elimination to form a C–C bond involved in the Pd-catalyzed aerobic C–H bond functionalization of heterocycles,^19^ which was taken from our previous mechanistic study^20^ of C–H bond functionalization.

Compared to the elimination on the Pd center (termed as Pd-elimination hereafter), the elimination on the Ti center (termed as Ti-elimination hereafter) is different despite the geometric similarities between **IM3** and **IM3a**, both featuring long C–X distances (X = C or N), and between **TS3** and **TS3a**, both describing the trend to form C–X bonds. The transition state **TS3a** in the Pd-elimination straightforwardly leads to the product and active catalyst (Pd^0^(CN^*t*^Bu)_2_), whereas **TS3** in the Ti-elimination proceeds to a comparatively stable complex **IM4** (which can be considered as a masked Ti^II^ complex, *vide infra*). The liberation of the pyrrole product from **IM4** *via* direct dissociation is not easy, costing ∼30.0 kcal mol^–1^. Instead, it preferentially takes place by interchanging with azobenzene, as depicted by **TS4**, with a much lower barrier (15.4 kcal mol^–1^). Geometrically, as shown by the bond length evolution of the key bonds (N–C^1^/Ti–C^1^/Ti–N) from **IM3** to **TS3** to **IM4** (Fig. 3A), the C^1^–N bond is formed steadily, but the Ti–C^1^ and Ti–N bonds are only elongated by less than 0.2 Å. If TiCl_2_Py moiety in **IM3** is replaced with Pd(CN^*t*^Bu)_2_, Pd-elimination to form a C–N bond (Fig. 3C) is the same as Pd-elimination to form a C–C bond (Fig. 3B), and thus the differences between the Ti- and Pd-eliminations are not due to the different substrates but are due to the TM centers (*i.e.*, Pd *versus* Ti). These differences encouraged us to characterize the Ti-elimination more deeply.

**Fig. 4:**
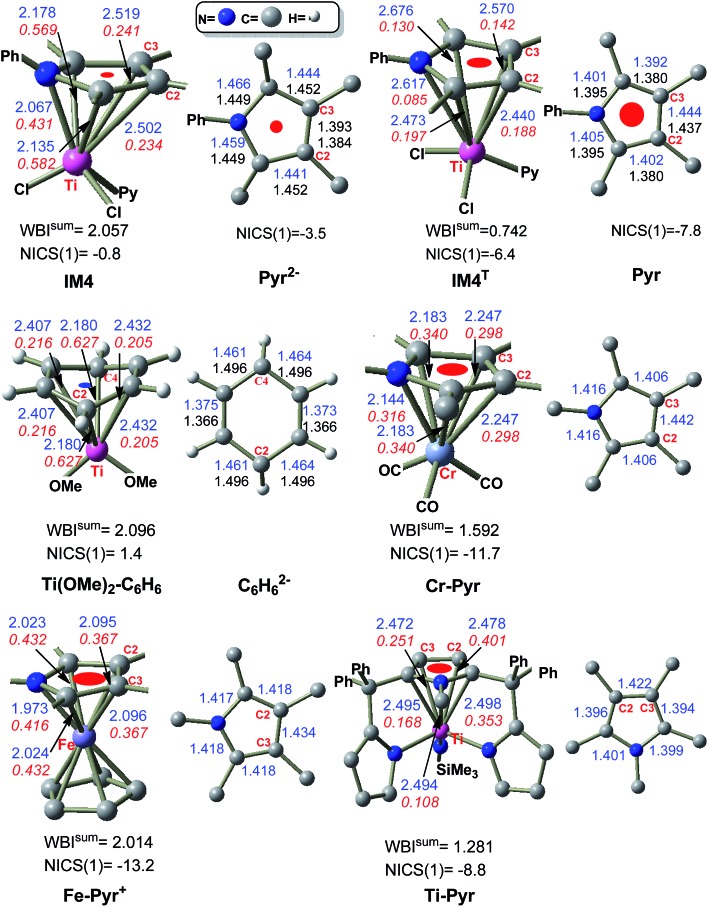
Optimized structures, together with the key bond lengths (blue values) in angstroms. The blue values in **Pyr^2–^**, **Pyr**, and **C_6_H_6_^2–^** are the bond lengths of the pyrrole ring in **IM4**, **IM4^T^**, and **Ti(OMe)_2_–C_6_H_6_**, respectively. NICS(1) is the NICS value (in ppm) at a point 1.0 Å above the ring center, respectively. The red italic values are WBIs and WBI^sum^ is the sum of the individual WBIs.

**Scheme 3:**
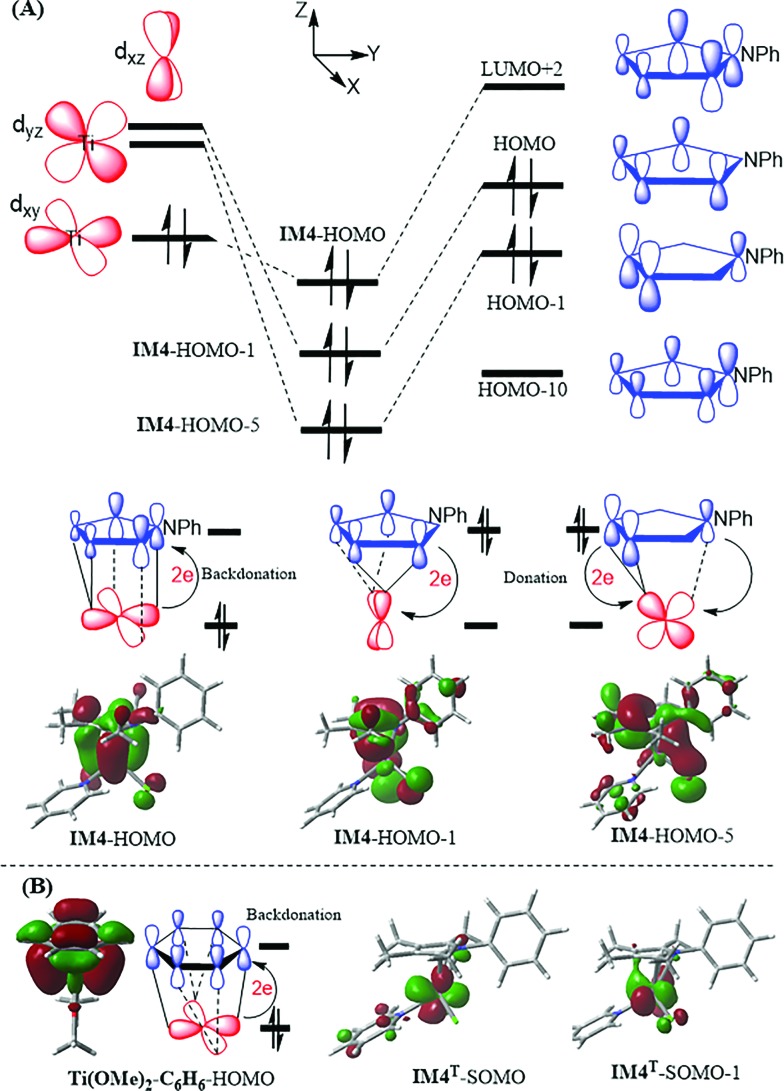
(A) Tracing the interactions of the molecular orbitals of pyrrole with those of TiCl_2_Py, resulting in **IM4**. (B) HOMO of **Ti(OMe)_2_–C_6_H_6_** and SOMOs of **IM4^T^**.

**IM3** and **TS3** in the Ti-elimination are similar to their counterparts (**IM3a/IM3b** and **TS3a/TS3b**) in the Pd-eliminations (Fig. 3); thereby we first focused on the abnormal complex **IM4**. The optimized structure of **IM4** is displayed in Fig. 4. In agreement with the large dissociation energy of pyrrole from **IM4**, the bond lengths between Ti and the atoms (N and C^1^–C^4^) of the pyrrole ring signify a tight bonding between the pyrrole ring and Ti center. The sum of the individual Wiberg bond indices (WBI^sum^) of these bonds reaches 2.057, indicating a double covalent bond nature, collectively involving the five atoms of the pyrrole ring. To characterize the covalent bonding in **IM4**, we analyzed how the pyrrole ring interacts with TiCl_2_Py. As illustrated in Scheme 3A, the HOMO of **IM4** originates from the symmetry-allowed interaction between the in-plane occupied d_*xy*_ orbital of titanium and the LUMO+2 of pyrrole (the LUMO and LUMO+1 of pyrrole are only relevant to the phenyl group), indicating a back-donation from Ti to the pyrrole ligand. The symmetry-allowed interactions of the unoccupied perpendicular Ti d_*xz*_/d_*xy*_ orbital with the occupied HOMO/HOMO–1 of the pyrrole ligand result in HOMO–1/HOMO–5 of **IM4**, respectively, which exemplify the donation from the pyrrole ligand to Ti. Other occupied orbitals involving less significant donation are not shown in Scheme 3A. On the basis of frontier molecular orbital interactions, we attributed the double covalent bond character between Ti and pyrrole in **IM4** to bilateral donation and back-donation.

Conventionally, a reductive elimination to form a C–X bond returns two of the four electrons in the TM–C and TM–X bonds to the TM center as a lone pair, thus reducing the TM center by two oxidation numbers^6–9^ (for example, see Fig. 3B and C). In contrast, the MO interaction analyses unraveled that the Ti-elimination back-donates the two electrons to the nascent pyrrole product. The differences between the Ti- and Pd-eliminations intrigued us to question what may happen if the two electrons return to the Ti center as adopted in the reductive elimination in late TM catalysis. We considered the triplet counterpart of **IM4**. Surprisingly, the HOMO–LUMO gap of **IM4** is very small (20.2 kcal mol^–1^, compared to 53.1 kcal mol^–1^ of **1cat**), indicating that **IM4** may have a lower triplet counterpart. Indeed, a 3.3 kcal mol^–1^ lower triplet (namely, **IM4^T^**) could be located, which is opposite to the cases of late TMs. For example, the triplet Pd^0^(CN^*t*^Bu)_2_ is 56.4 kcal mol^–1^ higher than its singlet counterpart. The reliability of M06-L//B3LYP calculations in ranking the singlet and triplet was validated by computing the experimentally well-characterized TiCl_2_(Py)_4_ complex. In agreement with the experimental results,^21^ the ground triplet of the complex was predicted to be 9.0 kcal mol^–1^ more stable than its singlet (Fig. S8†). Other levels of DFT calculations also predicted that **IM4^T^** is lower than **IM4** (Table S1†). In contrast to **IM4**, in which the two electrons resulting from the reductive elimination occupy bonding HOMO (see **IM4**–HOMO in Scheme 3A), the two electrons in **IM4^T^** singly occupy two MOs (see **IM4^T^**–SOMO and **IM4^T^**–SOMO–1 in Scheme 3B) dominated by Ti d orbitals, resulting in a spin density of *ρ* ^α^ = 1.70 on the Ti center. Furthermore, the two singly occupied orbitals of **IM4^T^** are very close in orbital energy (0.67 kcal mol^–1^), which is in favor of a ground triplet (*i.e.*, **IM4^T^**).

The two electrons in **IM4**, resulting from the elimination, form a bonding interaction *via* back-donation, whereas the two electrons in **IM4^T^** are the non-bonding ones. To characterize the back-donation effects, Fig. 4 compares the differences between **IM4** and **IM4^T^**. It could be found that (i) the pyrrole ring in **IM4** coordinates to the Ti center tighter than that in **IM4^T^**, as shown by the shorter Ti–C^1^/Ti–N/Ti–C^4^ atomic distances (2.135/2.067/2.178 Å) in **IM4** than those (2.473/2.617/2.676 Å) in **IM4^T^** and the greater WBI^sum^ (2.057) of **IM4** than that (0.742) of **IM4^T^**, and (ii) the pyrrole coordination in **IM4** is severely biased to the C^1^NC^4^ site and the N–C^1^ (1.466 Å) and N–C^4^ (1.459 Å) bonds in **IM4** are apparently longer than those (1.401 and 1.405 Å, respectively) in **IM4^T^**, which is in line with the orbital interaction pattern of the back-donation (see **IM4**–HOMO in Scheme 3A).

The back-donation even affects the aromatic nature of the pyrrole ring. To demonstrate this, the dianionic (**Pyr^2–^**) and neutral (**Pyr**) pyrrole were optimized (Fig. 4). It could be found that the pyrrole ring in **IM4** is best matched with **Pyr^2–^**, with both featuring the shortest C^2^–C^3^ bond, while the structure of **Pyr** is best fitted to the ring in **IM4^T^**, with both featuring the longest C^2^–C^3^ bond. These geometric matches imply that the pyrrole ring in **IM4** features a dianionic character, while it is neutral in **IM4^T^**. The nuclear independent chemical shift (NICS)^13^ has been proven to be a good criterion to measure the aromaticity of cyclic molecules. For example, at the present calculation level, the prototypical aromatic molecule benzene has a negative NICS(1) value of –10.3 ppm (NICS(1) representing the NICS value at a point 1.0 Å above the benzene ring), whereas the antiaromatic cyclobutadiene has a positive NICS(1) value of 15.1 ppm. Neutral pyrrole (**Pyr**) is aromatic with an NICS(1) value of –7.8 ppm. In contrast, the NICS(1) value of the pyrrole ring in **IM4** is insignificant (–0.8 ppm), indicating that the ring loses its aromaticity due to the back-donation. Because the pyrrole ring in **IM4^T^** is a neutral ligand only featuring donation, the NICS(1) value of the ring is –6.4 ppm, indicating that the ring maintains its aromaticity. It is interesting to mention that the donation does not affect the aromatic character of the ring, whereas back-donation does. The loss of aromaticity of the pyrrole ring in **IM4** rationalizes why **IM4**, which possesses favorable back-donation bonding, is conversely 3.3 kcal mol^–1^ higher than **IM4^T^** without back-donation. To validate this, ethylene–TiCl_2_Py complex was used to avoid the interference of aromaticity stabilization. As expected, singlet **C_2_H_4_–TiCl_2_Py^S^** was 12.1 kcal mol^–1^ lower than triplet **C_2_H_4_–TiCl_2_Py^T^**.

While the pyrrole ligand in **IM4** shows a dianionic character, pyrrole often acts as a neutral six-electron donor, forming η^5^-coordination TM complexes. To understand the difference and to reaffirm the dianionic character of **IM4**, we compared **IM4** with the experimental TM complexes (see **Cr–Pyr**,^22*a*^ **Fe–Pyr^+^**,^22*b*^ and **Ti–Pyr** ^22*c*^ in Fig. 4). In **Cr–Pyr** and **Fe–Pyr^+^**, the pyrrole ligand serves as a six-electron donor, endowing the complexes with an 18-electron count, whereas **Ti-Pyr** is a standard Ti^IV^ complex featuring Ti=NSiMe_3_ imido group and a 16-electron count. Geometrically, the bond length alternations in the pyrrole ligands of these experimental complexes match that in free **Pyr** rather than in **Pyr^2–^** (see Fig. 4). Consistently, the pyrrole ligands in these complexes have large negative NICS(1) values ranging from –8.8 to –13.3 ppm, which are significantly greater than that (–0.8 ppm) in **IM4**. Moreover, no occupied molecular orbital featuring significant back-donation could be found. Thus, a key difference between **IM4** and these experimental complexes is the back-donation in **IM4**. The comparisons demonstrate that the pyrrole ligand in **IM4** is indeed different from the neutral pyrrole ligand in these experimental complexes, possessing a dianionic character.

The viability of **IM4** is also supported by experimental facts. Although no low-valent titanium complex with pyrrole as a ligand has been reported to date, low-valent titanium complexes with arene as a ligand,^23^ as well as other low-valent early TM complexes,^24^ have been experimentally prepared. Most recently, Fortier *et al.* ^23*a*^ demonstrated that such a low-valent titanium complex could promote C(sp^3^)–H bond oxidative addition to perform transfer hydrogenation. In terms of the oxidation state of titanium in these complexes, the assignment is somewhat ambiguous, probably due to the lack of a rigorous way to assign the oxidation state of an organometallic complex, particularly for a non-classical complex. In the literature, these arene-coordinated low-valent titanium complexes have been considered as masked Ti^II^ complexes or Ti^II^ complexes with a significant Ti^IV^ character. However, Power *et al.* ^23*b*^ assigned the Ti^IV^ oxidation state to their Ti{N(H)Ar^i^Pr_6_}_2_ complex because of the observed diamagnetic nature of the complex. Nevertheless, regardless of what oxidation state could be assigned properly, a common feature of these low-valent complexes was the back-donation. Scheme 3 includes the molecular orbital (see **Ti(OMe)_2_–C_6_H_6_**–HOMO) related to the back-donation in a truncated model complex of arene-coordinated low-valent titanium complexes.

**Fig. 5:**
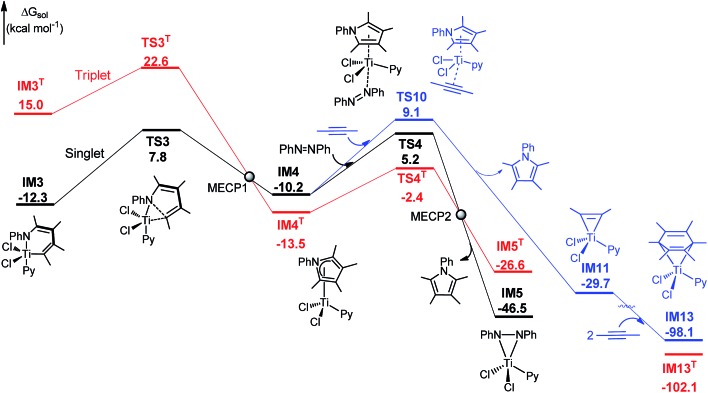
Free energy profile for C–N bond formation (stage III), with key bond lengths (in angstroms) of **IM3**, **TS3**, and **IM4**. For optimized structures of all the stationary points, see Fig. S1, S9 and S10.†

**IM4^T^** is lower than **IM4**, and thus the Ti-elimination transition state **TS3** may proceed to **IM4^T^** through spin-orbit coupling (Fig. 5),^25^ although the process is spin-forbidden. Fig. 5 shows the singlet-triplet potential energy surface crossing. Extensive calculations revealed that, except for **IM4** and **TS4**, all the intermediates and transition states in Fig. 1 and 3A have triplet states significantly higher than their singlet counterparts (Fig. S9†). We located the MECPs (minimum energy crossing points) between the two surfaces, using the MECP-location program developed by Harvey's group.^15^ At **MECP1**, the RMS SOC was predicted to be 38.6 cm^–1^ (SI2), which is much smaller than the value (*ca.* 250 cm^–1^ at a doublet-quartet MECP) that was considered to allow hopping for a molybdenum complex.^26^ An SOC value of 427 cm^–1^ at a triplet-quintet MECP was reported for β-hydride elimination of an iron(ii)–alkyl complex.^27^ In short, the spin–orbit coupling between the singlet and triplet in the present reaction is not strong, and thus the C–N bond formation stage may mainly proceed along the singlet energy surface. An effort was made to locate **MECP2** but we could not reach a converged structure. Because the stage mainly proceeds on the singlet surface, we expect that **MECP2** would not play an important role in this transformation.

It was assumed that azobenzene and alkyne may act as a redox-active ligand to promote the C–N bond formation. By displacing the Py ligand in **IM3** with azobenzene and alkyne, as well as PhCF_3_ (the solvent molecule), we located the relevant pathways. The energetic results, given in Fig. S11,† show that these pathways are kinetically much less favorable than the black pathway in Fig. 3A by at least 11.0 kcal mol^–1^, excluding these possibilities. Therefore, azobenzene/alkyne/PhCF_3_ are not effective to serve as redox-active ligands to promote C–N bond formation.

Examining the favorable pathway in Fig. 1 and 3A, one or more Py ligands stay(s) coordinated to the Ti center. Given the fact that the system contains other electron donors (*i.e.*, alkyne, azobenzene, and PhCF_3_), we examined whether these donors can coordinate to the Ti center to result in more stable complexes, and thus deviate the favorable pathway. As detailed in Table S2,† these possibilities can be excluded safely.

### 3.3 Further discussion on the difference between Ti-elimination and Pd-elimination

After characterizing the electronic structures of **IM4** and **IM4^T^**, we now seek to further understand the difference between the Ti- and Pd-eliminations. Referring to Fig. 3C, the Pd-elimination from **IM3b** to Pd^0^(CN^*t*^Bu)_2_ + free pyrrole is a two-electron reduction process, reducing the Pd^II^(CN^*t*^Bu)_2_ moiety in **IM3b** to Pd^0^(CN^*t*^Bu)_2_. Referring to Fig. 3A, if the pyrrole ring in **IM4** and the PhNNPh moiety in **IM5** could be considered as neutral electron donors, the entire process from **IM3** to **IM4** to **IM5** + free pyrrole would also be a two-electron reduction process, reducing the Ti^IV^Cl_2_Py moiety in **IM3** to Ti^II^Cl_2_Py in **IM4** and **IM5**. Thus, the bottom line is whether the pyrrole ring in **IM4** and the PhNNPh moiety in **IM5** could really be considered as neutral ligands. In Section 3.2, we demonstrated the dianionic character of the pyrrole ring in **IM4** and that the PhNNPh unit in **IM5** is apparently a dianionic ligand, as assigned by Tonks *et al.* (see Scheme 1). The difference between Ti-elimination and Pd-elimination can be attributed to the lower electronegativity of titanium than palladium. Generally, as reductive elimination proceeds, the TM moiety (*e.g.*, the Pd(CN^*t*^Bu)_2_ moiety in **IM3b**) gradually gains electron density. For the Pd-elimination, due to the great electronegativity of palladium, the Pd moiety is able to stabilize the growing electron density throughout the entire process; thus Pd-elimination leads to the pyrrole directly and, moreover, releases the active catalyst Pd^0^(CN^*t*^Bu)_2_. For Ti-elimination, particularly in the early stage, because TiCl_2_Py is dominated by the Ti^IV^ character, it is still capable of stabilizing the gained electron density, and the elimination should proceed in a way similar to that of the Pd-elimination. As the elimination progresses further, more and more electron density accumulates on the TiCl_2_Py moiety. However, due to the small electronegativity of titanium, the TiCl_2_Py moiety is no longer able to stabilize the gained electron density and a portion of electron density must be accommodated in an energetically more favorable way. Principally, there are two ways to accommodate the excessive electron density on TiCl_2_Py. One way is through back-donation of the electron density to the pyrrole ring, forming a favorable bonding interaction, which is the role of **IM4**. Another way is to become a triplet by taking advantage of the stabilization effect of exchange correlation energy, which is one of the reasons why **IM4^T^** is more stable than **IM4**. Because the spin–orbit coupling is not strong, the latter way is not feasible (*vide supra*) and the former way is thus adopted. To complete the two-electron reduction for eventually releasing pyrrole product from **IM4**, one way is to enforce the pyrrole ring to dissociate from **IM4**; however, this is energetically unfavorable, costing about 30.0 kcal mol^–1^ due to the bonding interactions of donation and back-donation. A mild way is to use an oxidant to pull the back-donated electrons back, which is the role of azobenzene, oxidizing the masked Ti^II^ intermediate (**IM4**) to a genuine Ti^IV^ complex (**IM5**), while simultaneously, the back-donated electrons are withdrawn to the Ti center.

The stronger ability of Pd(CN^*t*^Bu)_2_ than TiCl_2_Py in stabilizing the gained electron density is also reflected by the fact that the singlet Pd^0^(CN^*t*^Bu)_2_ is 58.0 kcal mol^–1^ lower than its triplet counterpart, whereas the singlet Ti^II^Cl_2_Py species is 18.1 kcal mol^–1^ higher than its triplet counterpart. To stabilize the two non-bonding electrons in TiCl_2_Py, the two electrons have to singly occupy two molecular orbitals in order to take advantage of the stabilization effect of exchange correlation.

**Fig. 6:**
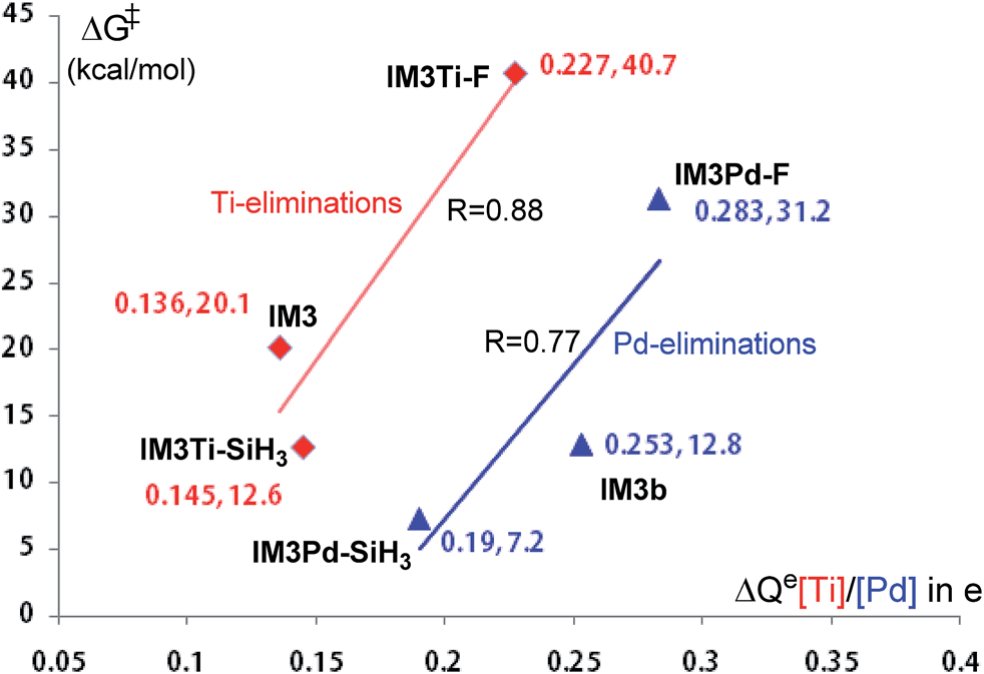
Correlation of the elimination barriers with the electron gains on the [Ti]/[Pd] moiety from the **IM3/IM3b** analogs to the **TS3/TS3b** analogs. [Ti] = TiCl_2_Py and [Pd] = Pd(CN^*t*^Bu)_2_. For example, in the case of **IM3** elimination, the electron gain Δ*Q* ^e^ = –[*Q* ^NBO^([Ti] at **TS3**) – *Q* ^NBO^([Ti] at **IM3**)].

It should be noted that although the Ti-elimination features back-donation and donation, the general trend of two-electron reduction cannot be altered. Thus, the substitution effect on the elimination barrier of Ti-elimination is similar to that in Pd-elimination. For example, the Ti-elimination barriers of **IM3Ti–F**, **IM3**, and **IM3Ti–SiH_3_** (see Scheme 3) are 40.7, 20.1, and 12.6 kcal mol^–1^, respectively and are in the same order as that of Pd-elimination barriers of **IM3Pd–F**, **IM3b**, and **IM3Pd–SiH_3_** being 31.2, 12.8, and 7.2 kcal mol^–1^, respectively. The high barriers in the case of X = F can be attributed to the strong electron-withdrawing nature of fluorine atoms, which hinders the tendency of electron transfer to the TM moiety (*i.e.*, Pd(CN^*t*^Bu)_2_ or TiCl_2_Py) before reaching the transition states. Fig. 6 correlates the electron gains of the TM moieties from the **IM3/IM3b** analogs to the **TS3/TS3b** analogs with the elimination barriers. Similar to Hammond's postulation, for Ti-eliminations and Pd-eliminations, it is generally true that more the electrons the TM moiety gains, the higher the elimination barrier. It is interesting to mention that **IM4Ti–F** should possess stronger back-donation than **IM4** and **IM4Ti–SiH_3_**, but the elimination barrier of **IM3Ti–F** is higher than that of the others, indicating that the back-donation is only one of the factors determining the barrier for Ti-elimination. Other factors include the intrinsic effects (TM^*n*^/TM^*n*–2^ reduction) like in conventional reductive elimination and donation. In the case of X = F, as the back-donation is relatively strong, the donation should be relatively weak.

**Scheme 4:**
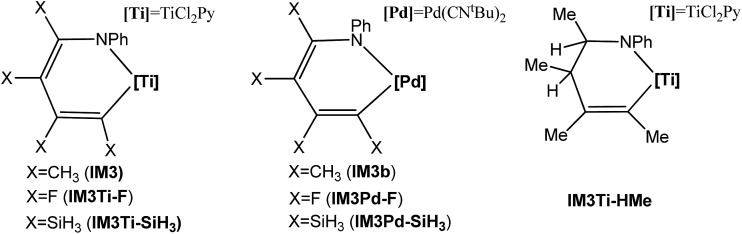
Hypothesized molecules used to study the substitution effect of the substituents. The optimized structures of the **IM3/IM3b**, **TS3/TS3b** and **IM4/IM4b** analogs are displayed in Fig. S12.†

In Ti-elimination, both donation and back-donation bonding interactions favor the stabilization of the system, thus facilitating the elimination. To prove this, we saturated one of the remote double bonds in **IM3**, resulting in **IM3Ti–HMe** (see Scheme 4). We did not saturate another C=C double bond because the elimination would form a C(sp^3^)–N bond, which is not comparable with **IM3** elimination to form C(sp^2^)–N. As expected, its elimination barrier (25.5 kcal mol^–1^) is higher than the 20.1 kcal mol^–1^ of **IM3** elimination.

**Scheme 5:**
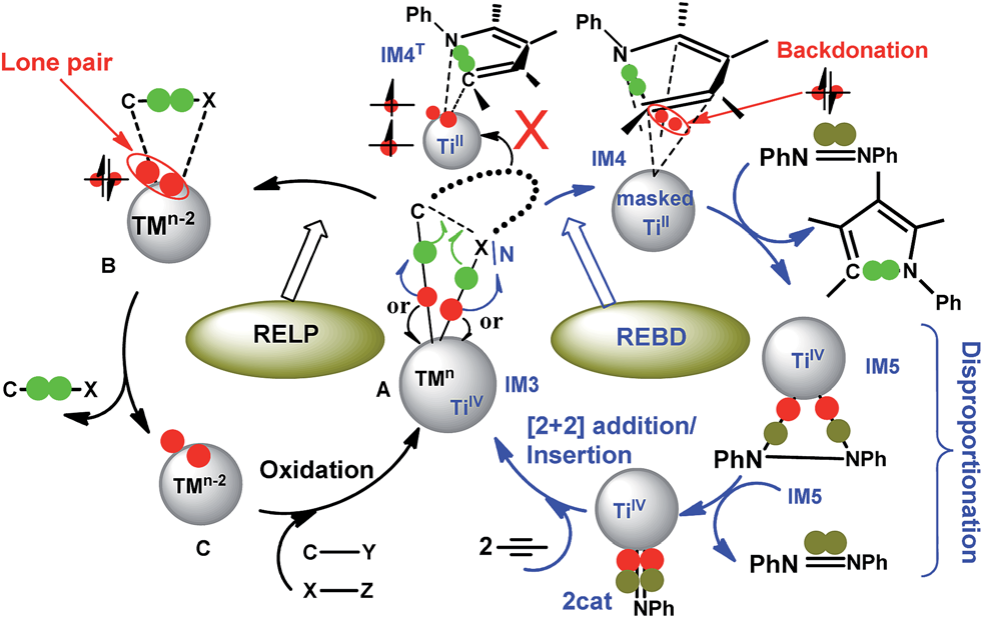
Comparisons of conventional reductive elimination *via* forming a lone pair on the TM center (RELP, left) with the present reductive elimination *via* back-donation (REBD, right). Note that **B** generally involves weakly bound complexes and may not exist in some cases (*e.g.*, those in Fig. 3B and C).

In an alternative/formal way, Scheme 5 compares conventional reductive elimination with the present reductive elimination by focusing on how the two pairs of electrons in the TM–C and TM–X/N bond in **A/IM3** flow. As the C–X/N bond formation proceeds, a pair of electrons (in green) would be used to form a new C–X/N bond. The competition to pull electrons between TM and the forming product determines the destination where the remaining pair of electrons (in red) ultimately goes. For late TMs with strong electronegativity (the left cycle), the pair of electrons prefers migrating to the TM center to become a lone pair, reducing TM^*n*^ in **A** to TM^*n*–2^ in **B**. We denote this way of reductive elimination as RELP in Scheme 5. In Ti-elimination (the right cycle), due to the small electronegativity of titanium, the pair of electrons prefer back-donating to a symmetry-allowed unoccupied orbital of the pyrrole ring (see **IM4**–HOMO in Fig. 4). We call this way of reductive elimination as REBD in Scheme 5. Note that in addition to the back-donation, **IM4** also features donation from pyrrole to the Ti center. As compared in Fig. 3, the barrier (20.1 kcal mol^–1^, **TS3** relative to **IM3**) for Ti-elimination is higher than that (12.8 kcal mol^–1^, **TS3b** relative to **IM3b**) for Pd-elimination, implying that the RELP adopted by late TMs is more effective to stabilize the two electrons than the REBD used by early TMs. The remaining pair of electrons could migrate to the Ti center in **IM4^T^**, but requires spin-flipping. Because the spin–orbit coupling to enable the spin-flip is weak, the process from **IM3** to **IM4^T^** *via* **TS3** is unlikely.

The process from **IM4** to **IM5** takes place *via* the oxidation of azobenzene, which oxidizes the masked Ti^II^ to Ti^IV^ and, moreover, pulls the two back-donated electrons back to the Ti center, forming two Ti–N bonds with the PhNNPh unit in **IM5**. This is why the pyrrole release from **IM4** prefers to undergo an interchange mechanism rather than a dissociative route (see Fig. 3A). On the basis of the mechanistic understanding of the Ti-elimination, we were able to conceive the necessary requirements for early TMs to undergo REBD elimination, including that (i) the product has symmetry-allowed empty orbitals to accommodate the two electrons from the elimination *via* back-donation and (ii) the substrate (*i.e.*, azobenzene) should possess a comparatively strong oxidizing ability to oxidize the electron-buffering intermediate (*e.g.*, **IM4**) for product release. To some extent, the pyrrole product acts like a redox-active ligand, reserving electrons during the process of forming a C–N bond from **IM3** to **IM4** and liberating the reserved electrons when reacting with an oxidant (azobenzene) from **IM4** to **IM5**.

Experimentally, it was reported that a trace amount of alkyne trimerization product (*i.e.*, hexamethylbenzene) could be obtained.^4^ Our predicted mechanism is able to explain this experimental observation. Referring to Fig. 5, as an alternative to azobenzene attacking **IM4** and giving **IM5**, the alkyne, serving as an oxidant, can also attack **IM4** *via* **TS10** (the blue pathway in Fig. 5), leading to the **IM5** analog (*i.e.*, **IM11**). As detailed in Fig. S9,† **IM11** can readily react with two alkynes, resulting in hexamethylbenzene after azobenzene or alkyne attacks **IM13**. Because of the weaker oxidizing ability of alkyne than azobenzene, **TS10** is 3.9 kcal mol^–1^ higher than **TS4**. The barrier difference (3.9 kcal mol^–1^) rationalizes why trace and only trace amounts of alkyne trimerization product (*i.e.*, hexamethylbenzene) could be produced (eqn (1)).

**Fig. 7:**
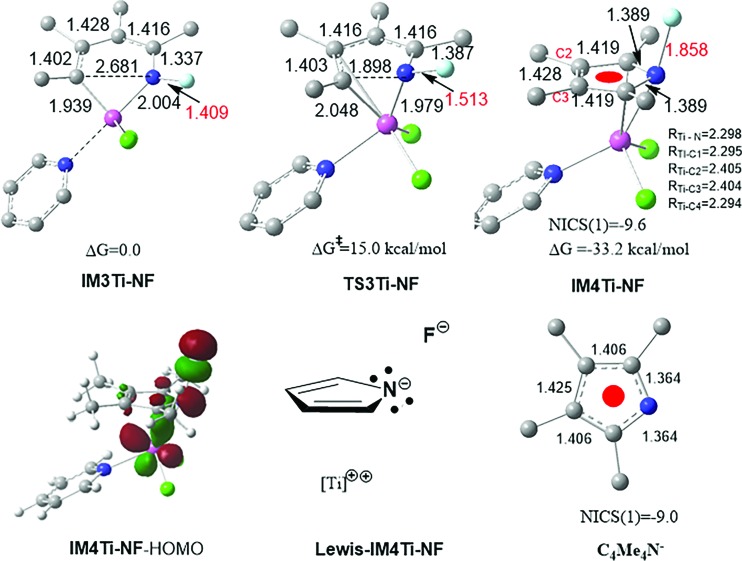
Optimized structures, together with the key bond lengths in angstroms. NICS(1) is the NICS value (in ppm) at a point 1.0 Å above the ring center.

As discussed above, the essence of REBD is that the two electrons resulting from the elimination are stabilized by a back-donation bonding interaction. There are other ways to stabilize the two electrons, such as the strategy of using a redox-active ligand. Herein, we show that a negative hyperconjugation^28^ can also stabilize the two electrons. We replaced the phenyl group in **IM3** with a F atom, resulting in **IM3Ti-NF** (see Fig. 7), and then calculated the elimination pathway from **IM3Ti-NF** to **TS3Ti-NF** to **IM4Ti-NF**. As compared in Fig. 7, **IM4Ti-NF** has a N–F bond length (1.858 Å) much longer than that (1.409 Å) in **IM3Ti-NF**, indicating the negative hyperconjugation described by **IM4Ti-NF**–HOMO. By comparing the five-membered ring in **IM4Ti-NF** with free **C_4_Me_4_N^–^** in terms of NICS(1) values (both having a NICS (1) value of *ca.* –9.0 ppm) and the bond length alternation pattern of the rings, we assigned the Lewis structure (Lewis–**IM4Ti-NF**) to **IM4Ti-NF**. Therefore, one difference between **IM3** and **IM3Ti-NF** eliminations is that the two electrons in the former elimination are stabilized by the back-donation bonding, whereas the two electrons in the latter elimination are stabilized by the negative hyperconjugation. The occurrence of the negative hyperconjugation further supports the dianionic character of the pyrrole ring in **IM4** and **IM4Ti-NF**. Thus, a more general principle for early TM catalysts to undergo reductive elimination is that the two electrons resulting from elimination must be stabilized, no matter what favorable interactions (*e.g.*, back-donation and negative hyperconjugation) could be used, rather than becoming a lone pair located at the TM center, as adopted in the late TM catalysis systems.

## 4. Conclusion

In summary, the present study reveals that the C–N bond formation in the titanium(iv)-catalyzed pyrrole synthesis from alkynes and diazenes takes place *via* an elimination pathway featuring donation and back-donation. Different from the conventional reductive elimination adopted by late TMs, where the two electrons resulting from elimination are stabilized by the TM center as a lone pair, the two electrons in the present reductive elimination are stabilized by donating to a symmetry-allowed unoccupied orbital of the forming pyrrole product. Because of the back-donation and donation, the elimination leads to a comparatively stable masked Ti^II^ complex (**IM4**) rather than the pyrrole product directly. **IM4** requires undergoing an additional oxidation step by azobenzene to liberate the pyrrole product. The triplet counterpart (**IM4^T^**) of **IM4** is more stable than **IM4**; however, the elimination is unlikely to lead to **IM4^T^** because it is spin-forbidden and the spin–orbit coupling to enable the hopping from the singlet to triplet energy surfaces is weak. The study provides an insight into the unconventional elimination mechanism. Although the weak electronegativity of titanium does not favor stabilizing the two formal electrons resulting from the reductive elimination, the same feature favors back-donation to allow the two electrons to donate to the nascent product easier. Alternatively, one may consider the pyrrole product as a redox-active ligand, reserving electrons resulting from the formal reductive elimination and releasing electrons when reacting with an oxidant. On the basis of the mechanistic understanding of the reaction, we conceived two requirements for early TMs to undergo similar elimination, including that (i) the forming product has a symmetry-allowed unoccupied orbital to accommodate the two electrons resulting from the elimination, which as a lone pair cannot be stabilized by early TMs and (ii) a comparatively strong oxidizing reagent (*e.g.*, azobenzene in the present case) is important to pull the back-donated electrons back, thus releasing the product. It should be noted that back-donation is one way to stabilize the two electrons resulting from the reductive elimination; these two electrons can also be stabilized *via* other ways, such as a negative hyperconjugation. The insights from the present study could provide another way to construct C–X bonds using early TM catalysts.
