# Supplementary material for: Differences between the elimination of early and late transition metals: DFT mechanistic insights into the titanium-catalyzed synthesis of pyrroles from alkynes and diazenes
Source: Chem Sci. 2016 Dec 22;8(3):2413–25. doi: 10.1039/c6sc04456e (PMC5369339; doi:10.1039/c6sc04456e)
Supplement: Supplementary file 1 [file SC-008-C6SC04456E-s001.pdf]

Electronic Supplementary Material (ESI) for Chemical Science.
This journal is © The Royal Society of Chemistry 2016

Supporting Information

What are the differences between eliminations of early and late transition metals? DFT mechanistic insights into the titanium-catalyzed synthesis of pyrroles from alkynes and diazenes

Jiandong Guo,[†] Xi Deng,[†] Chunyu Song,[†] Shuanglin Qu,[†] Yanfeng Dang,[†,‡], Zhi-Xiang Wang*[,†,‡]

[†]School of Chemistry and Chemical Engineering, University of Chinese Academy of Sciences, Beijing 100049, China

[‡]Collaborative Innovation Center of Chemical Science and Engineering, Tianjin 300072, China

Table of Contents

SI1: Fig.S1~Fig.S10 and Table S1 and Table S2 ....(S2~S15)

SI2: Details results for spin-orbit coupling calculations ....(S16~S18)

SI3: Cartesian coordinates, SCF energies, and free energies for all optimized structures ....(S19~S77)

SI1: Fig.S1~Fig.S10, Table S1 and Table S2

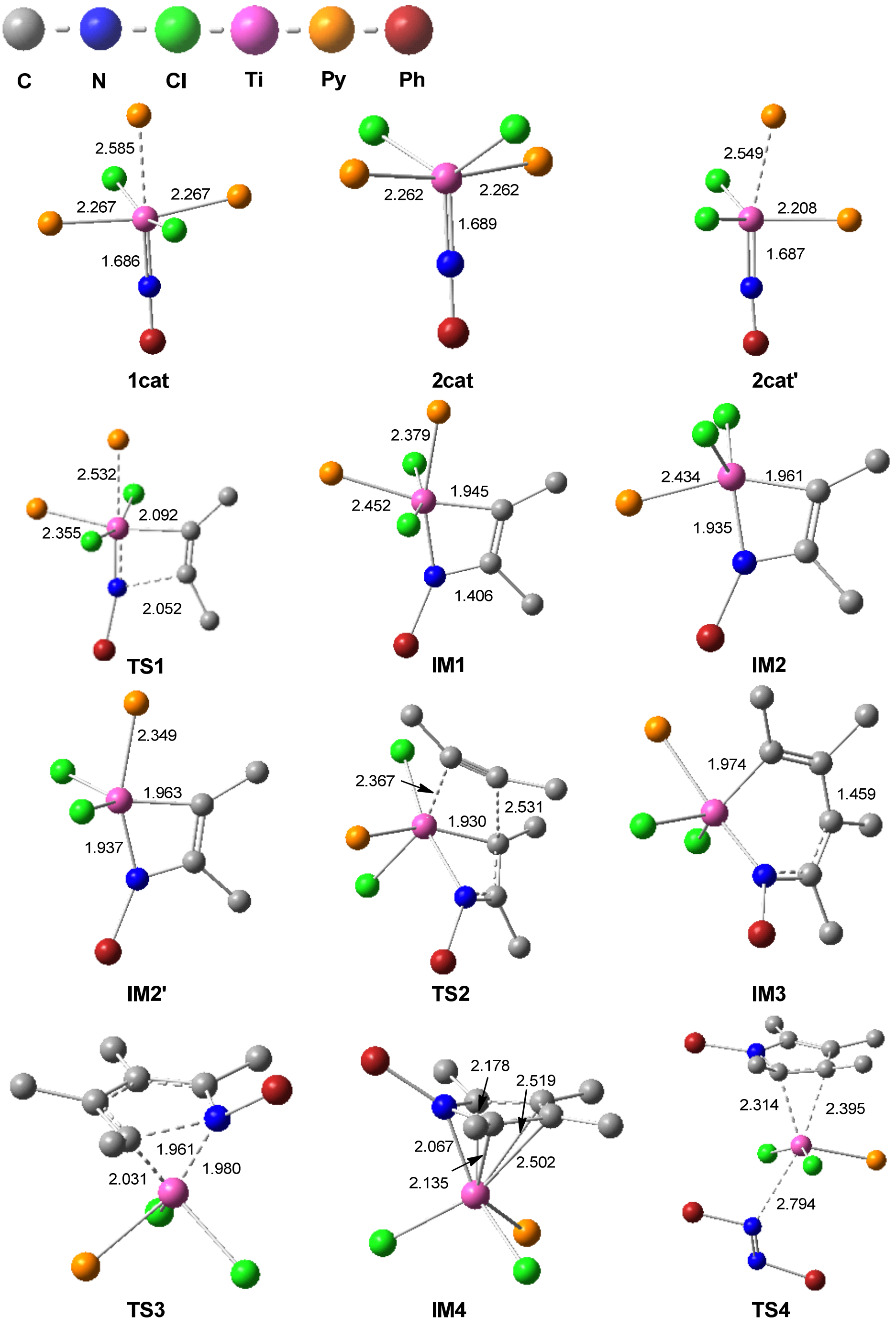

Fig.S1. B3LYP/6-31G(d,p) optimized structures of stationary points involved in Fig.1 and 3A, along with key bond lengths in angstroms. Trivial hydrogen atoms are omitted for clarity.

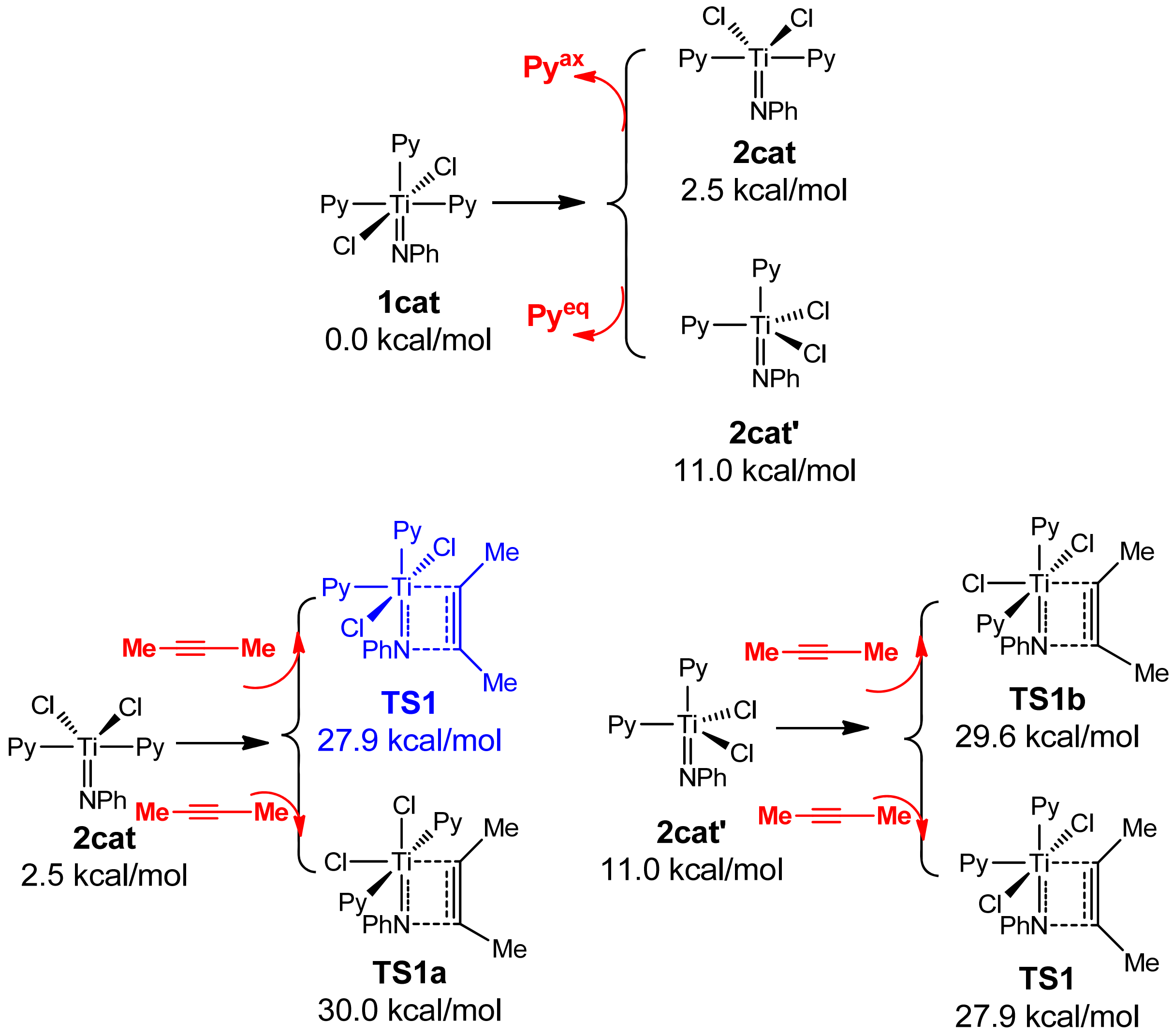

Fig.S2. Energetic comparisons for alkyne [2+2] addition to imido Ti=N bond of **2cat** and **2cat'**. Values are free energies relative to **1cat** and are mass balanced.

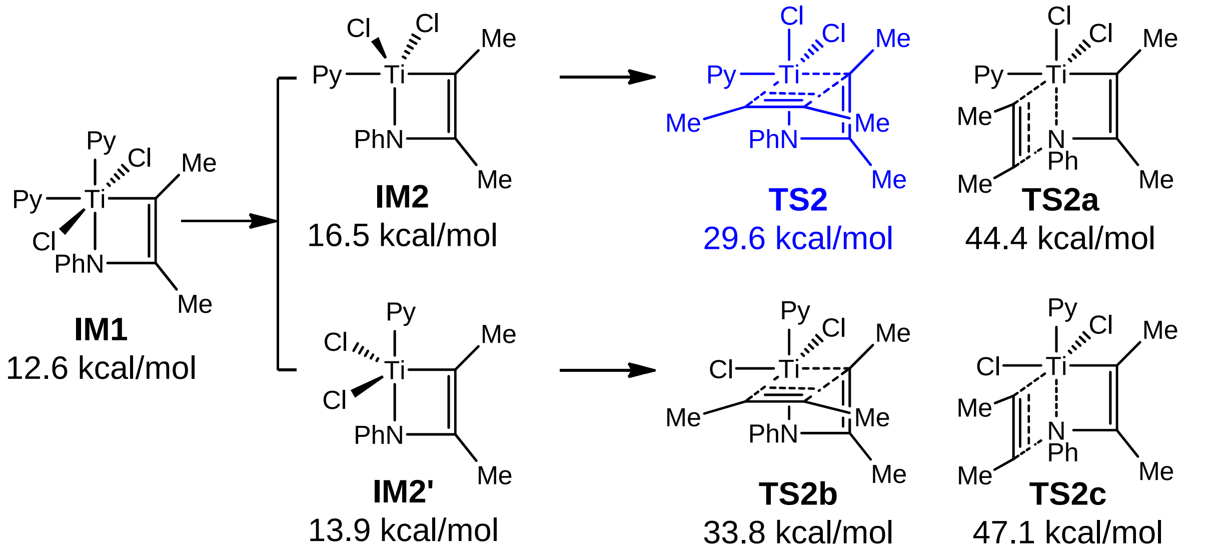

Fig.S3. Energetic comparisons for the insertions of alkyne into the Ti–C bond or Ti–N bond. Values are free energies relative to **1cat** and are mass balanced.

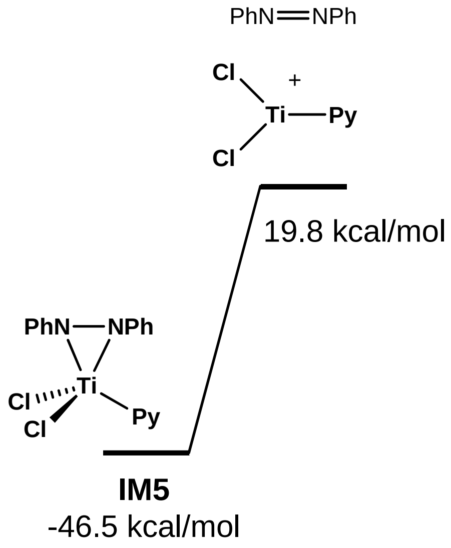

Fig.S4. Dissociation energy of azobenzene from **IM5**. Values are free energies relative to **1cat** and are mass balanced.

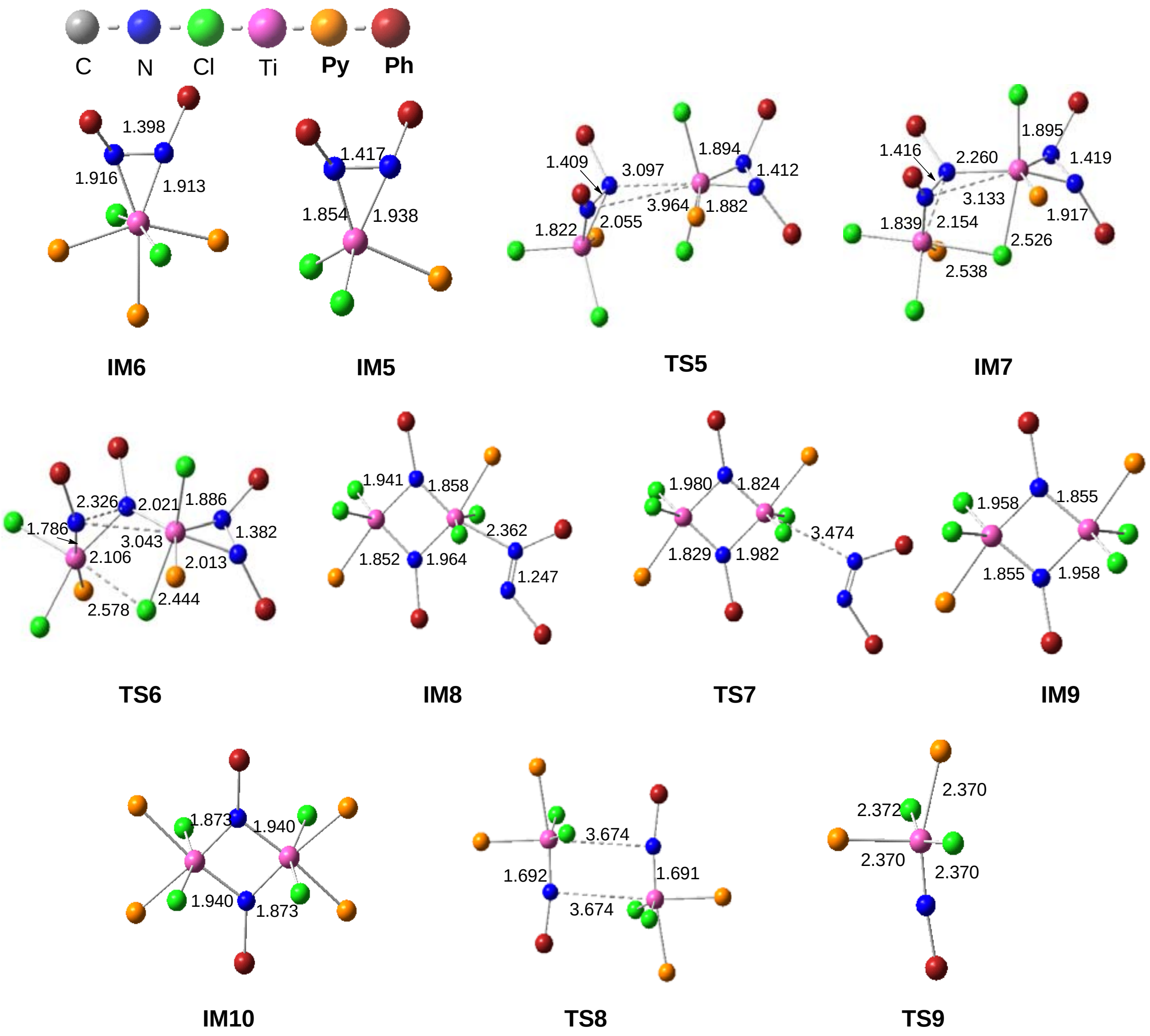

Fig.S5. B3LYP/6-31G(d,p) optimized structure of key stationary points involved in Fig.2, along with key bond lengths in angstroms. Trivial hydrogen atoms are omitted for clarity.

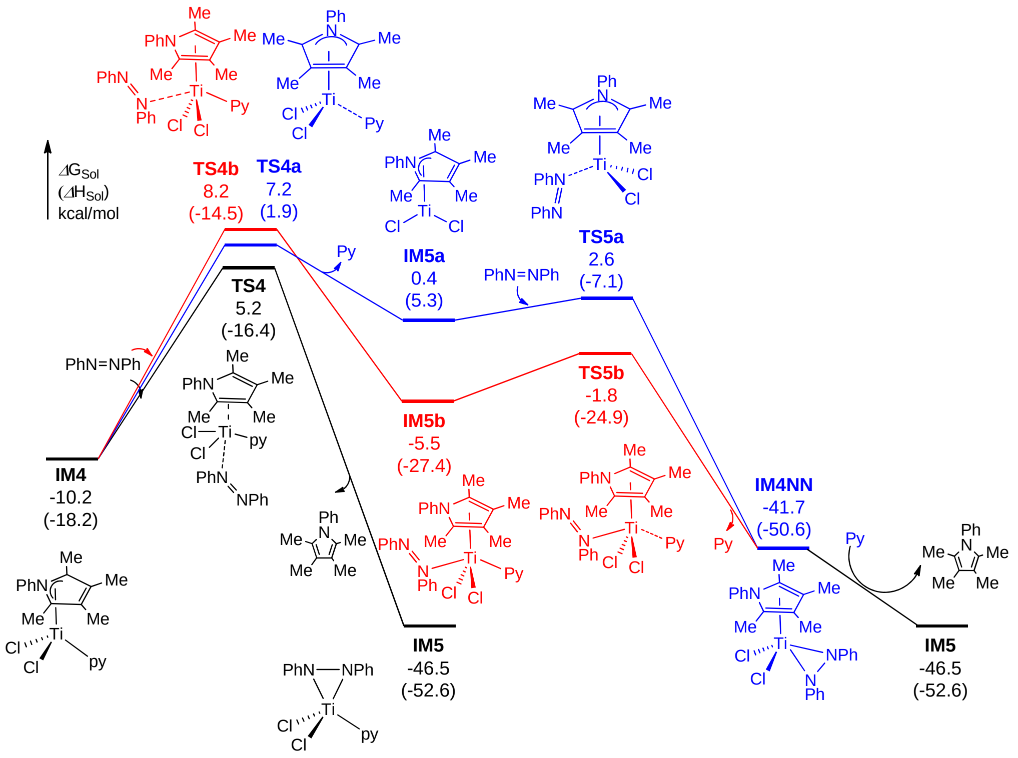

Fig.S6A. When PhNNPh is used to attack **IM4**, the alternative pathways to our reported **IM4→TS4→IM5**. Values are free energies relative to **1cat** and are mass balanced. Key optimized structures are displayed in Fig.S5B.

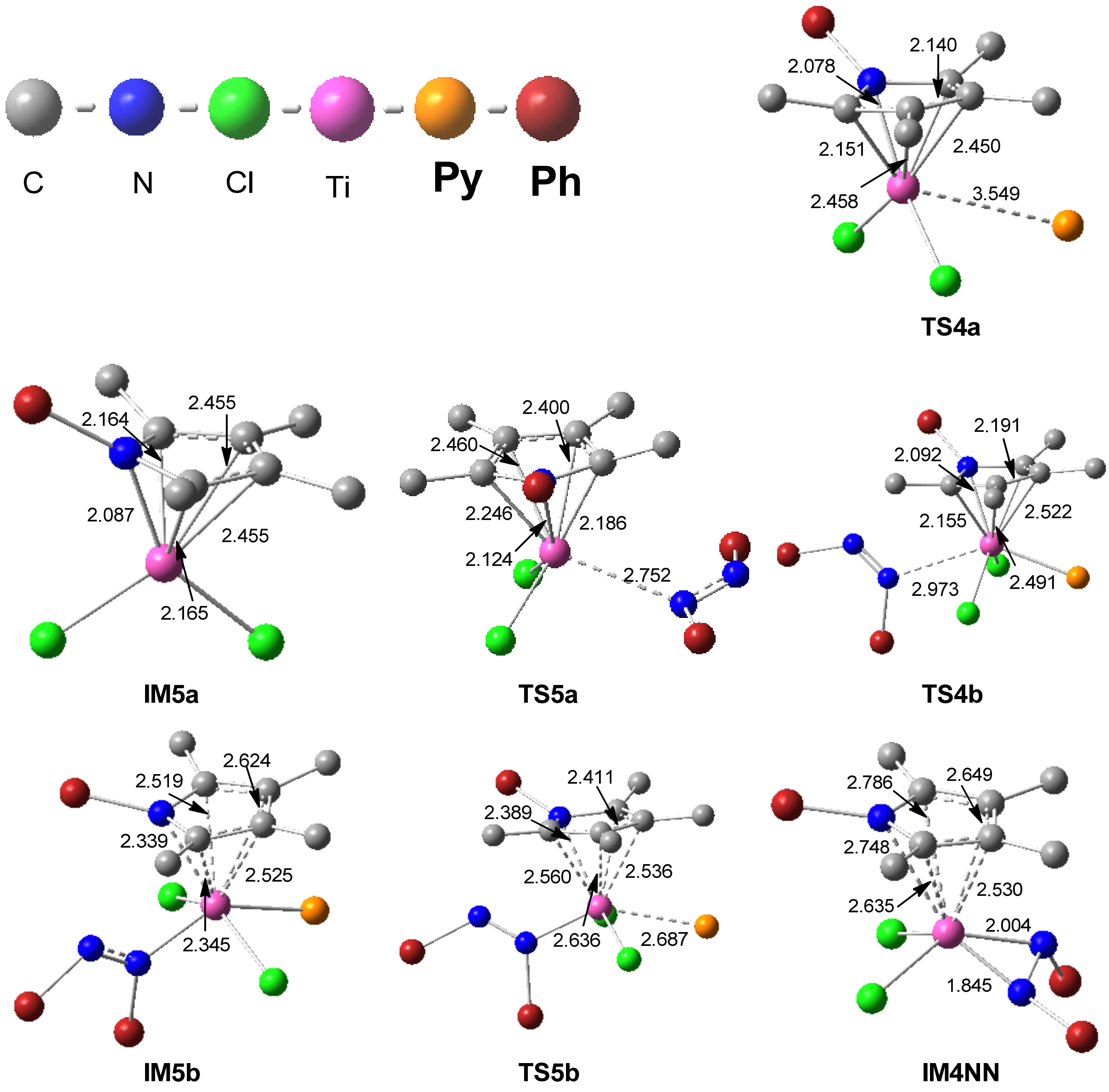

Fig.S6B. B3LYP/6-31G(d,p) optimized structure of key stationary points involved in Fig.S6A, along with key bond lengths in angstroms. Trivial hydrogen atoms are omitted for clarity.

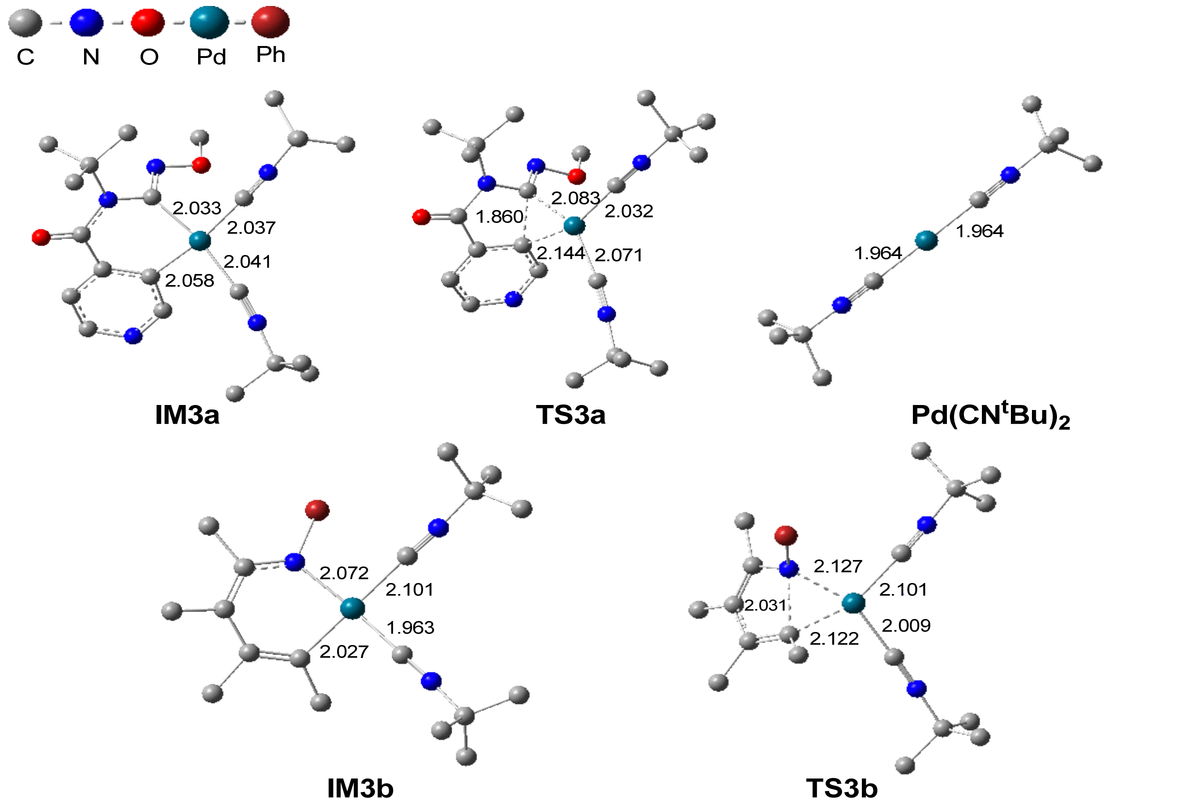

Fig.S7. B3LYP/6-31G(d,p) optimized structure of key stationary points in Fig.3B and 3C, along with key bond lengths in angstroms. Trivial hydrogen atoms are omitted for clarity.

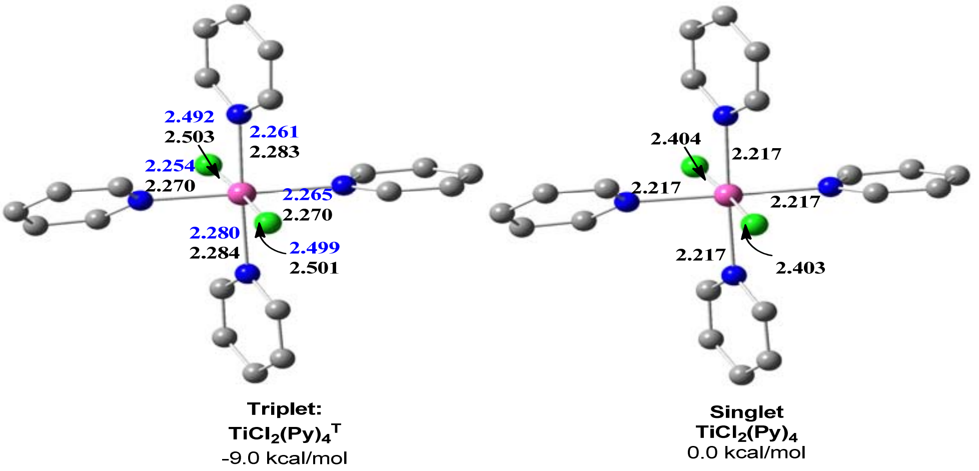

Fig.S8. B3LYP/6-31G** optimized structures and M06-L(PCM)/6-311++G(d,p) energetic results for the experimentally well-characterized Ti(II) complex ($TiCl_2(Py)_4$). Blue values are X-ray parameters.

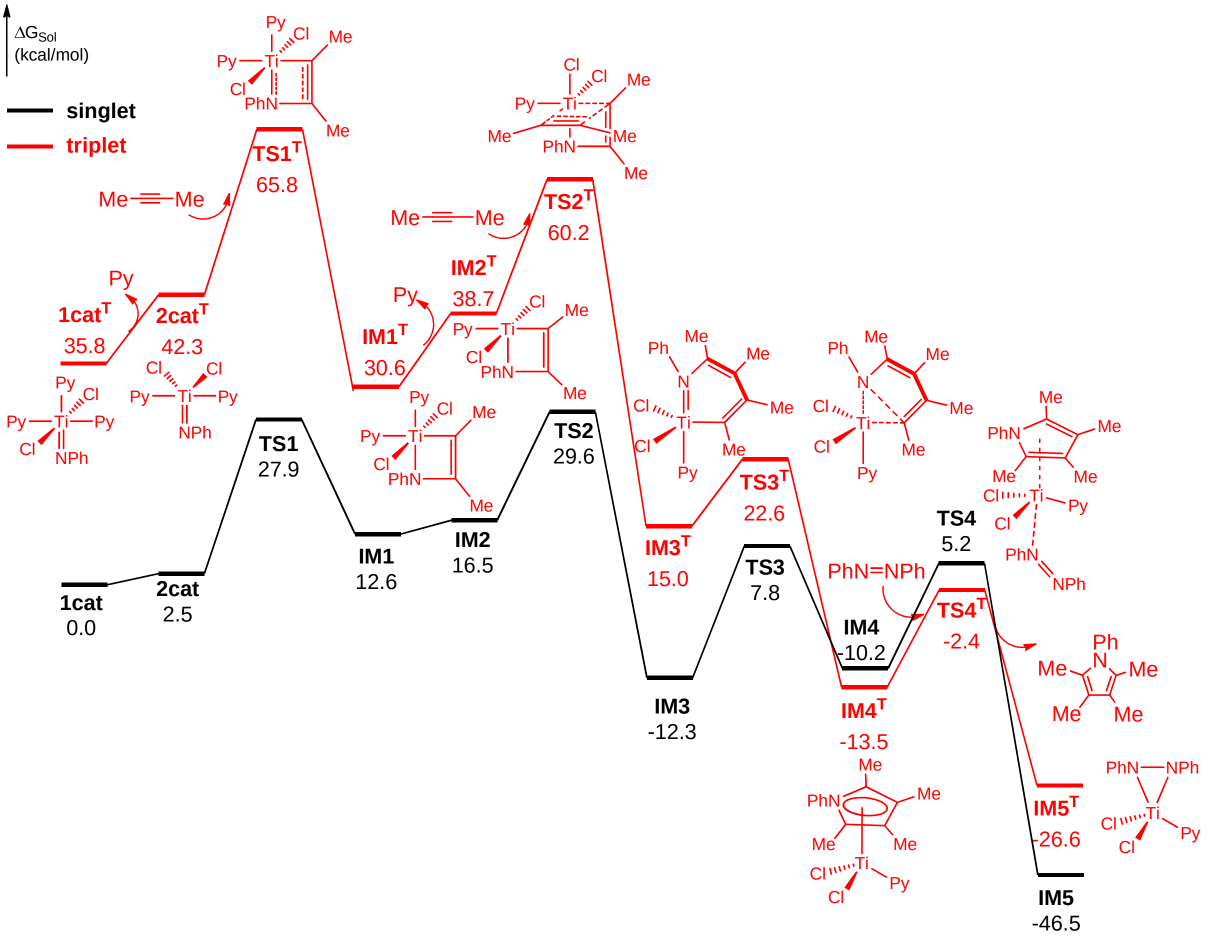

Fig.S9A. Free energy profiles of singlet and triplet for the first three stages (stage I~stage III in Scheme 1B). Values are free energies relative to **1cat** and are mass balanced. Optimized structures of singlet are displayed in Fig.S1. Optimized structures of triplet are displayed in Fig.S9B.

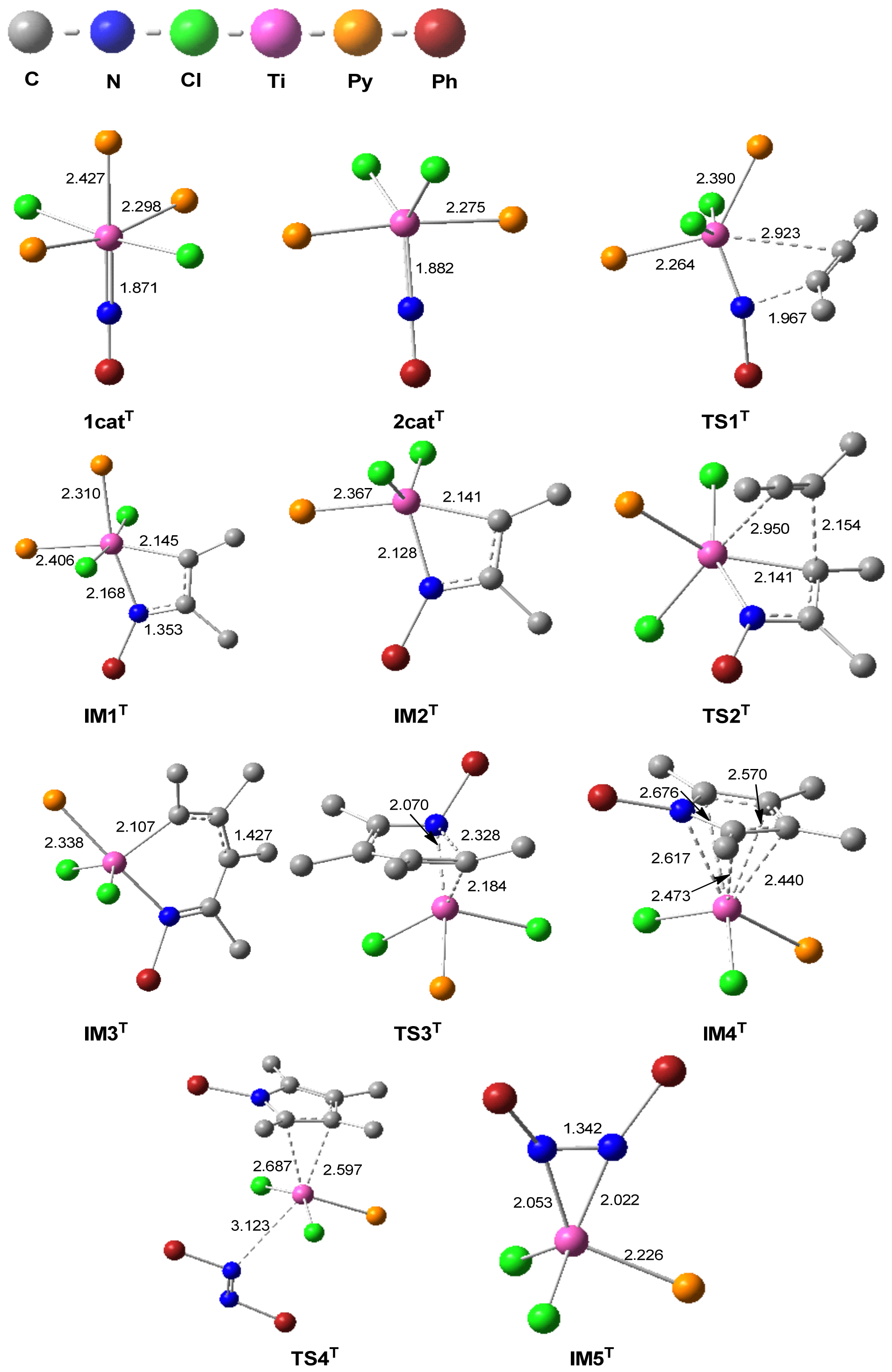

Fig.S9B. B3LYP/6-31G(d,p) optimized structures of stationary points (triplet) involved in Fig.S9A, along with key bond lengths in angstroms. Trivial hydrogen atoms are omitted for clarity.

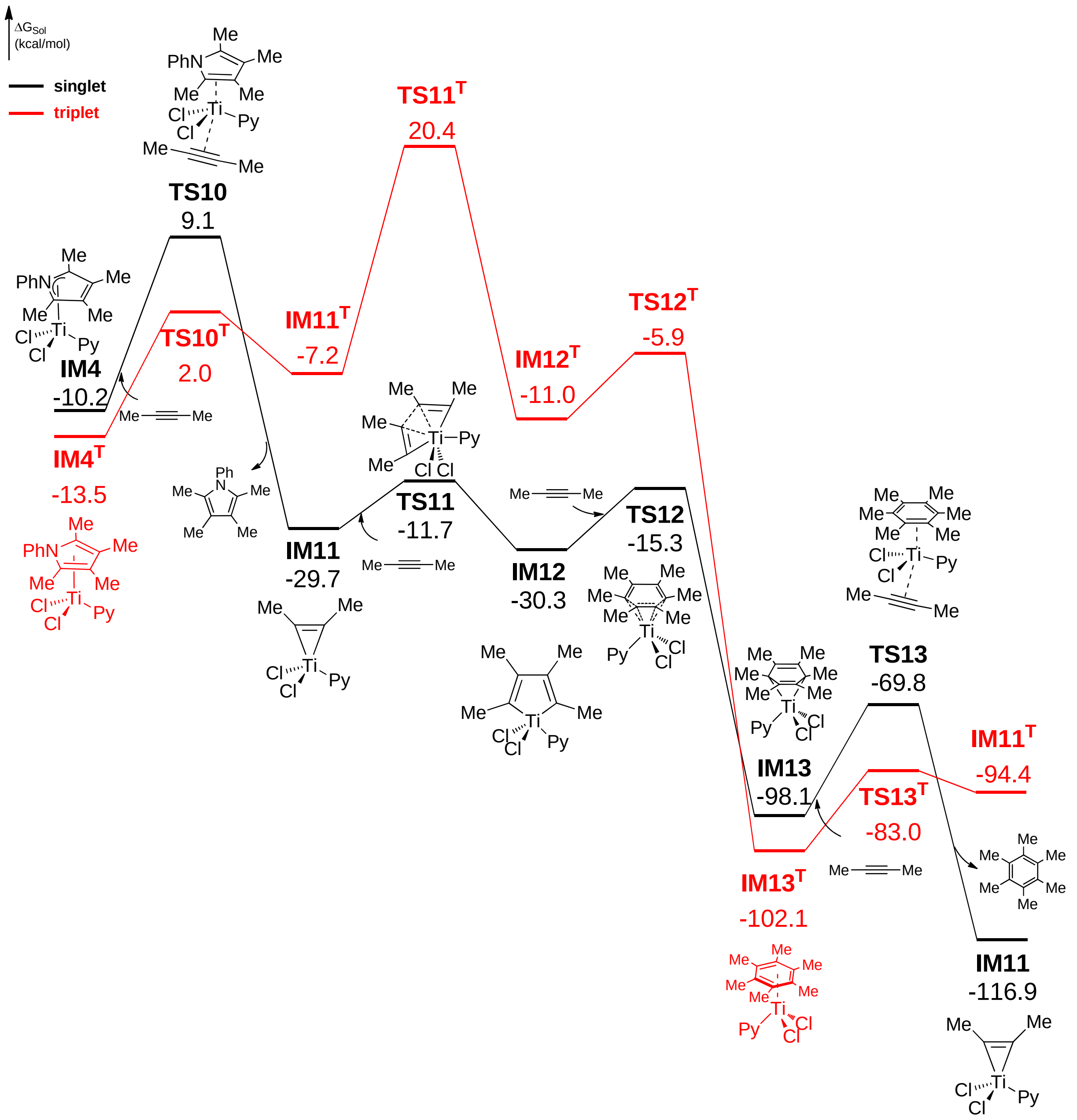

Fig.S10A. Free energy profiles of singlet and triplet for the alkyne trimerization. Values are free energies relative to **1cat** and are mass balanced. Optimized structures of stationary points are displayed in Fig.S10B.

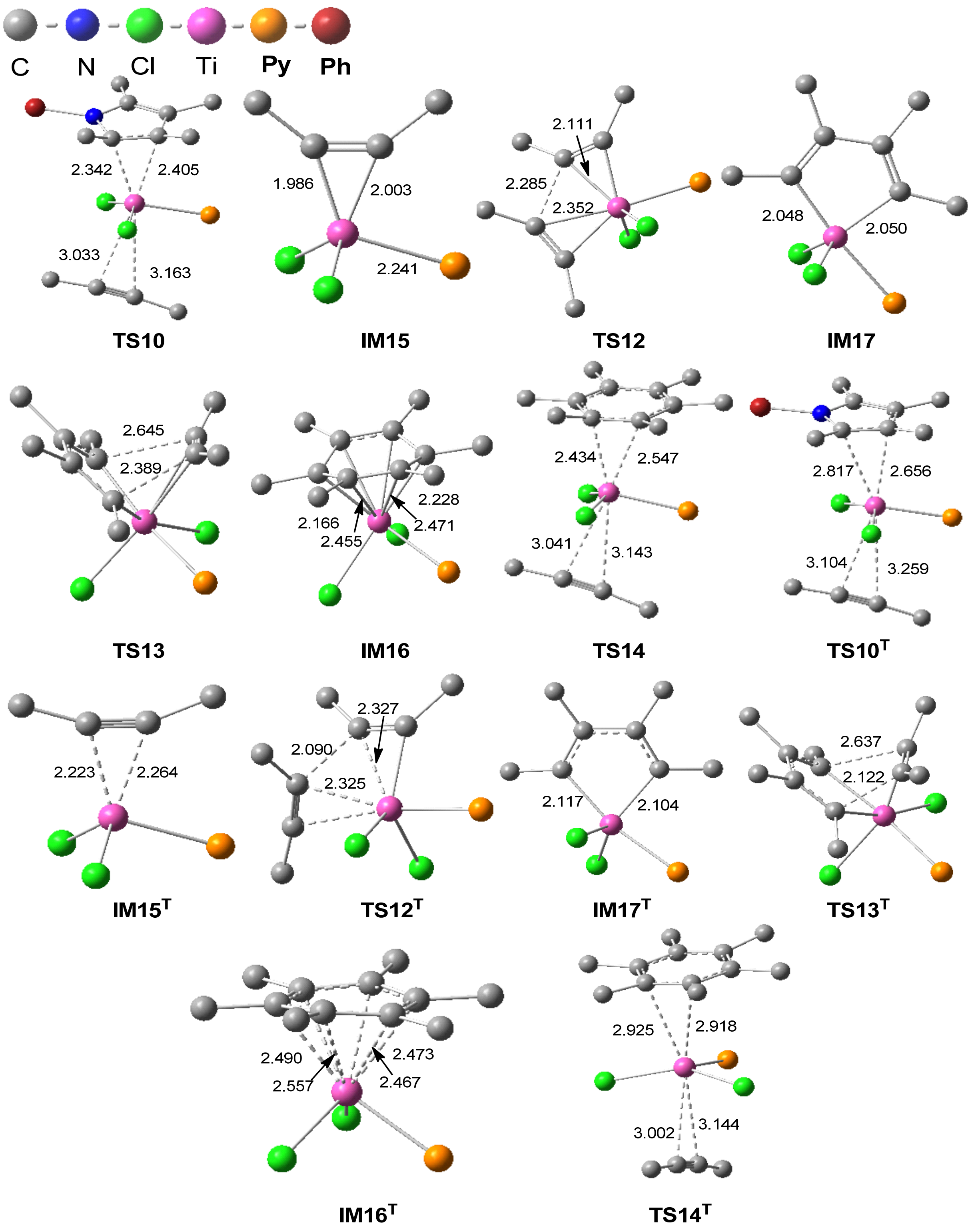

Fig.S10B. B3LYP/6-31G(d,p) optimized structures of stationary points involved in Fig.S10A, along with key bond lengths in angstroms. Trivial hydrogen atoms are omitted for clarity.

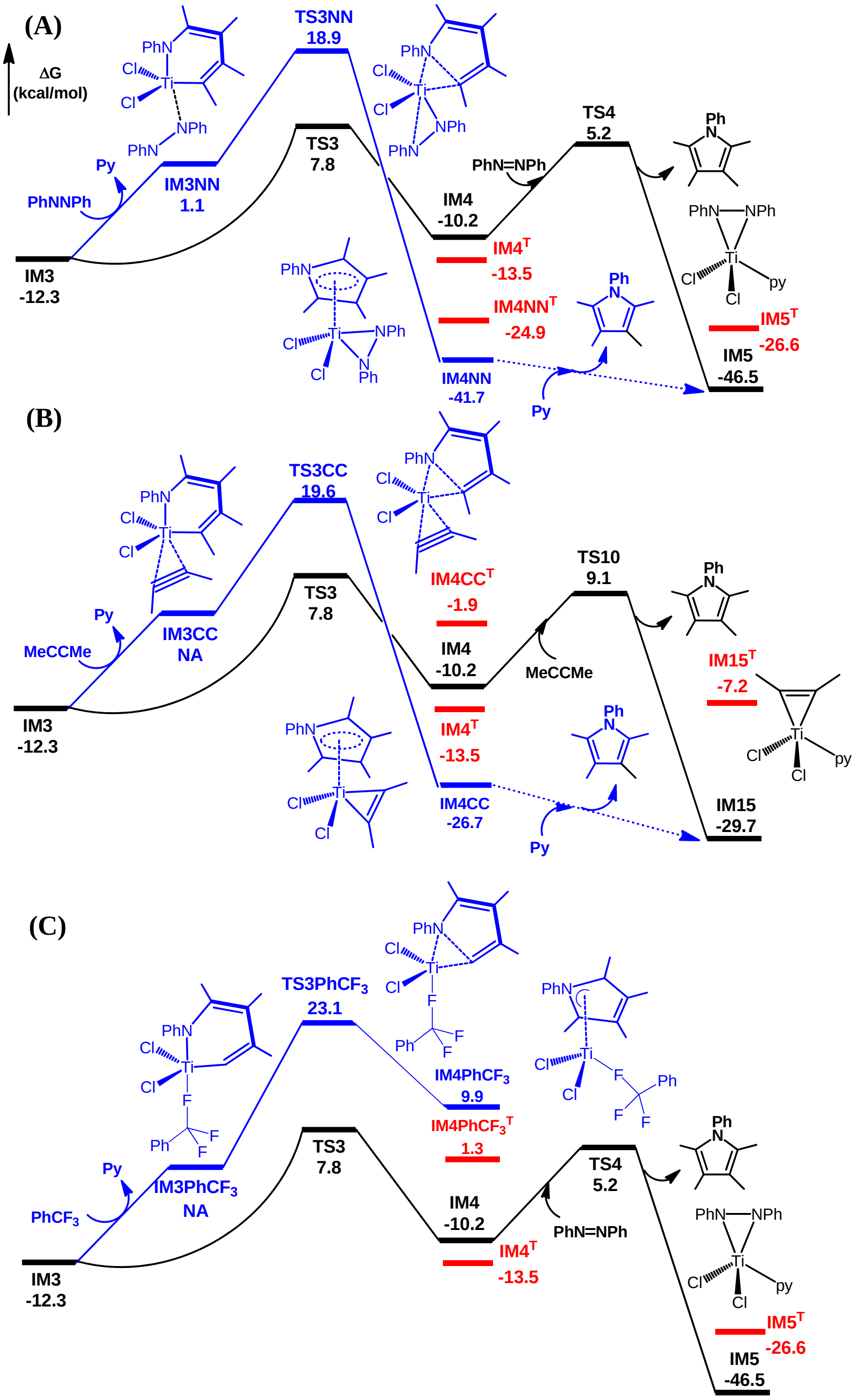

Fig.S11A. Free energy profile for the C–N bond formation pathways (the blue one) involving azobenzene,alkyne and $PhCF_3$ as a ligand. Optimized structures of stationary points are displayed in Fig.S11B.

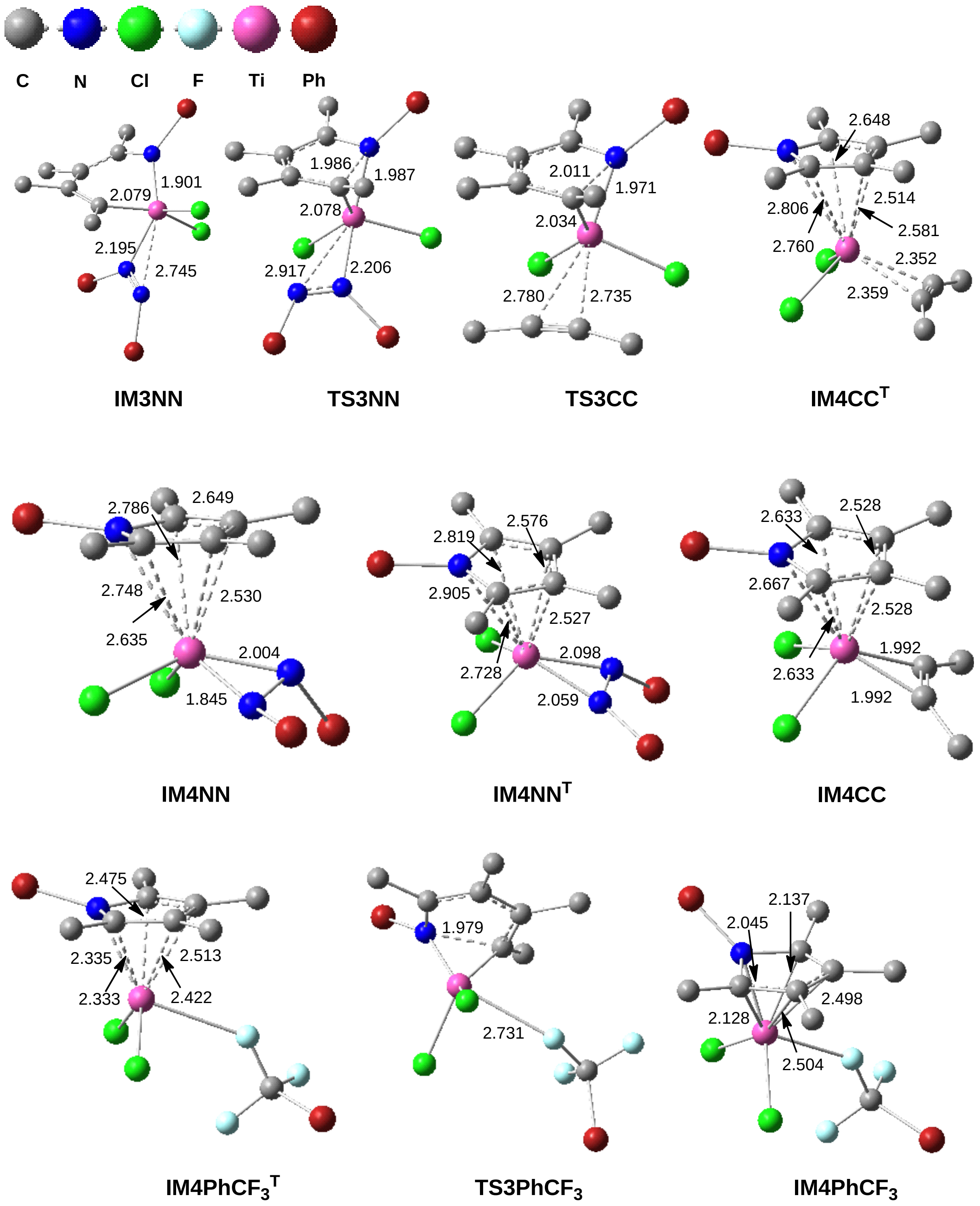

Fig.S11B. B3LYP/6-31G(d,p) optimized structures of stationary points involved in Fig. S11A, along with key bond lengths in angstroms. Trivial hydrogen atoms are omitted for clarity.

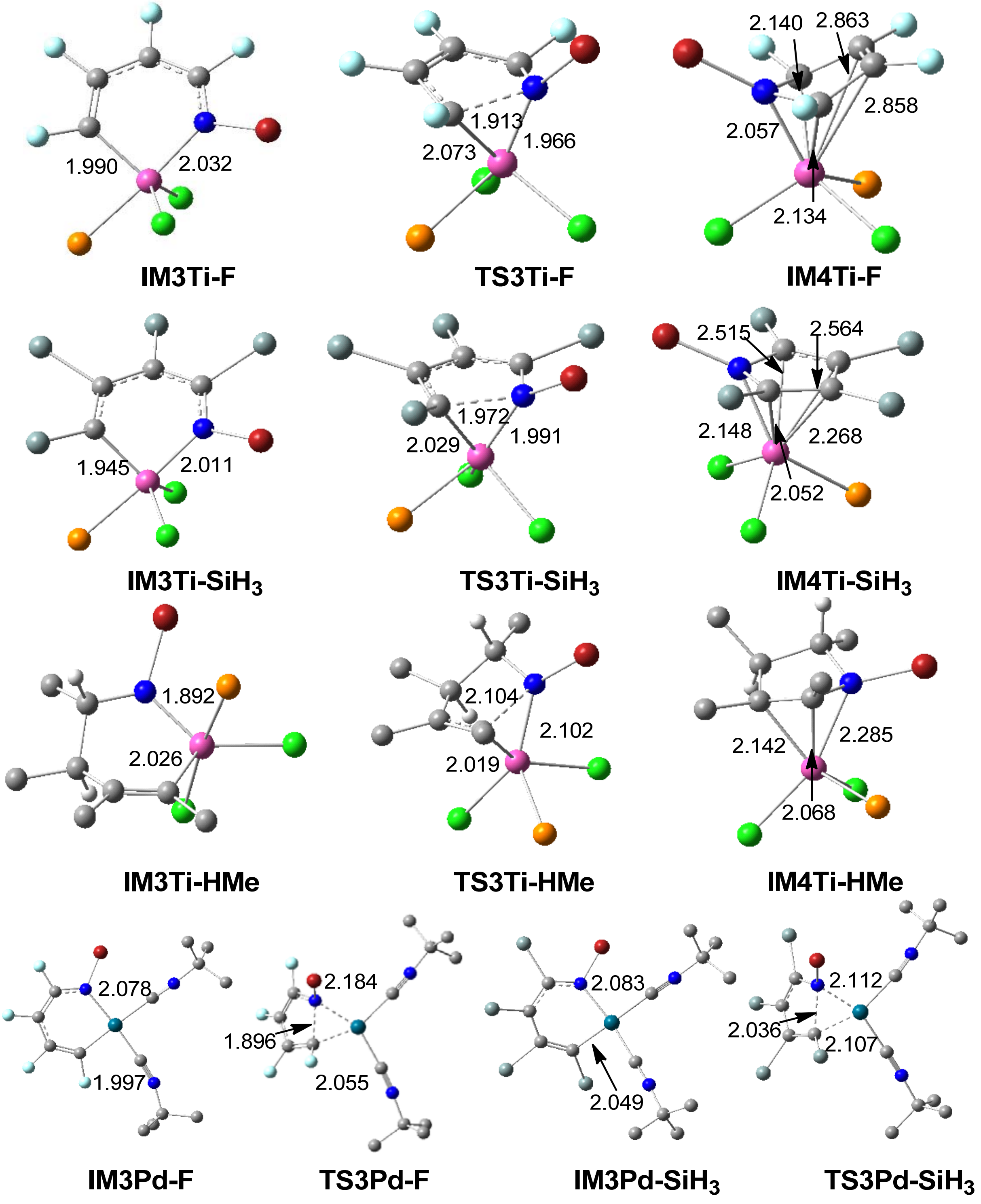

Fig.S12. B3LYP/6-31G(d,p) optimized structures of stationary points involved in Scheme 4, along with key bond lengths in angstroms. Trivial hydrogen atoms are omitted for clarity.

Table S1. Different levels of DFT calculations for **IM4^{T}** and **IM4.** The energies were refined by single-point energy calculations with solvent effects accounted by PCM solvent model in the experimentally used solvent ($PhCF_3$).

	M06-L/6-311++G** //B3LYP/6-31G**	B3LYP/6-311++G** //B3LYP/6-31G**	M06/6-311++G** //M06/6-31G**	BP86/6-311++G** //BP86/6-31G**	wB97XD/6-311++G** //wB97XD/6-31G**
G(IM4^{T})-G(IM4) (kcal/mol)	-3.3	-11.4	-4.8	-3.9	-10.4

Table S2: Thermodynamics cost (in kcal/mol) for displacing Py ligand with PhNNPh, MeCCMe, and $PhCF_3$. NA means that optimized structure could not located.

Entry 1	Entry 2	Entry 3	Entry 4
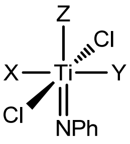<br><br>**1cat**	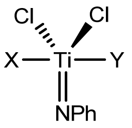<br><br>**2cat**	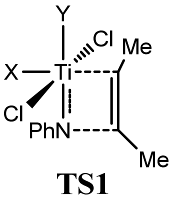<br><br>**TS1**	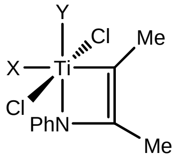<br><br>**IM1**
**X=Y=Z=Py ΔG_{Sol}=0.0**<br>X=Y=Py Z=PhNNPh ΔG_{Sol}=13.2<br>X=Z=Py Y=PhNNPh ΔG_{Sol}=15.2<br>Z=Py X=Y=PhNNPh ΔG_{Sol}=29.6<br>X=Py Y=Z=PhNNPh NA<br>X=Y=Z=PhNNPh NA<br>X=Y=Py Z=MeCCMe NA<br>X=Z=Py Y=MeCCMe ΔG_{Sol}=13.5<br>Z=Py X=Y=MeCCMe ΔG_{Sol}=26.6<br>X=Py Y=Z=MeCCMe NA<br>X=Y=Z=MeCCMe NA<br>X=Z=Py Y=$PhCF_3$ ΔG_{Sol}=16.9	**X=Y=Py ΔG_{Sol}=2.5**<br>X=Py Y=PhNNPh ΔG_{Sol}=17.1<br>X=Y=PhNNPh ΔG_{Sol}=31.6<br>X=Py Y=MeCCMe ΔG_{Sol}=15.[illegible]<br>X=Y=MeCCMe ΔG_{Sol}=28.2<br>X=Py Y=$PhCF_3$ ΔG_{Sol}=21.2	**X=Y=Py ΔG_{Sol}=27.9**<br>X=PhNNPh Y=Py ΔG_{Sol}=39.7<br>X=Py Y=PhNNPh ΔG_{Sol}=41.7<br>X=Y=PhNNPh ΔG_{Sol}=56.6<br>X=MeCCMe Y=Py NA<br>X=Py Y=MeCCMe NA<br>X=Y=MeCCMe NA<br>X=$PhCF_3$ Y=Py ΔG_{Sol}=39.3	**X=Y=Py ΔG_{Sol}=12.6**<br>X=Py Y=PhNNPh ΔG_{Sol}=20.9<br>X=PhNNPh Y=Py ΔG_{Sol}=21.0<br>X=Y=PhNNPh ΔG_{Sol}=34.9<br>X=MeCCMe Y=Py NA<br>X=Py Y=MeCCMe NA<br>X=Y=MeCCMe NA<br>X=Py Y=$PhCF_3$ NA
Entry 5	Entry 6	Entry 7	Entry 8
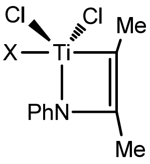<br><br>**IM2**	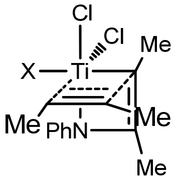<br><br>**TS2**	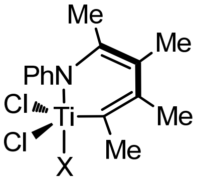<br><br>**IM3**	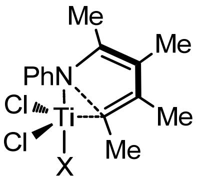<br><br>**TS3**
**X=Py ΔG_{Sol}=16.5**<br>X=PhNNPh ΔG_{Sol}=25.0<br>X=MeCCMe NA<br>X=$PhCF_3$ ΔG_{Sol}=27.5	**X=Py ΔG_{Sol}=29.6**<br>X=PhNNPh ΔG_{Sol}=44.2<br>X=MeCCMe NA<br>X=$PhCF_3$ NA	**X=Py ΔG_{Sol}=-12.3**<br>X=PhNNPh ΔG_{Sol}=1.1<br>X=MeCCMe NA<br>X=$PhCF_3$ NA	**X=Py ΔG_{Sol}=7.8**<br>X=PhNNPh ΔG_{Sol}=18.9<br>X=MeCCMe ΔG_{Sol}=19.6<br>X=$PhCF_3$ ΔG_{Sol}=23.1
Entry 9			

<table>
<tr>
<td>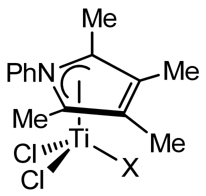

IM4</td>
<td rowspan="2">Note that only these complexes in case 1~3 (entry 9) are stable, but:
the complex in case 1 will proceed to IM5 in Figure 3 or 5
The complex in case 2 will proceed to IM5 in Figure 3 or 5 after a Py replaces pyrrole.
The complex in case 3 will proceed to IM11 in Figure 5. after a Py replaces pyrrole.
Thus, the formations of these complexes will not affect the pathways reported in the main text.</td>
</tr>
<tr>
<td>X=Py ΔG_{Sol}=-10.2 (Case 1)
X=PhNNPh ΔG_{Sol}=-41.7 (case 2)
X=MeCCMe ΔG_{Sol}=-26.7 (case 3)
X=$PhCF_3$ ΔG_{Sol}=9.9</td>
</tr>
</table>

SI2: Detailed results for spin-orbital coupling calculations

SI2a. Structure of MECP1

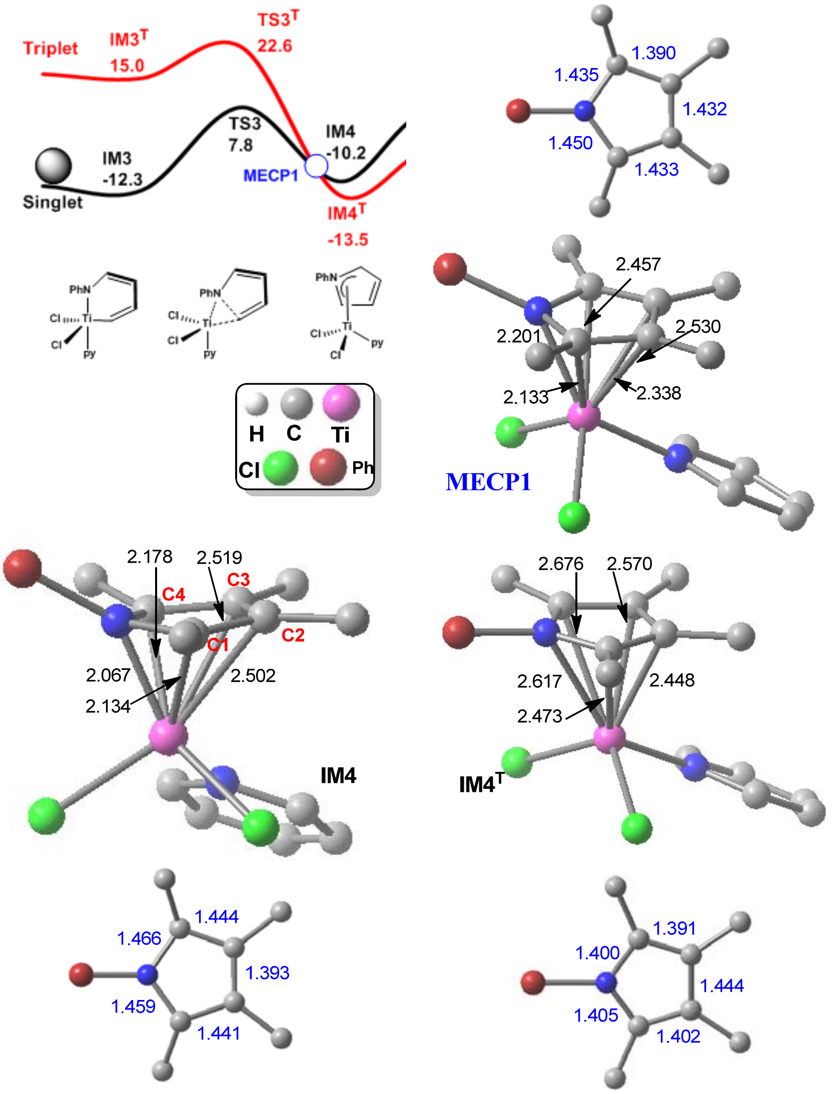

SI2b. Technical details for for spin-orbit coupling (SOC) calculations

Due to the relative large system (46 atoms in total) for high level wavefunction-based computations, the SOC value is calculated at MRCI/CASSCF(2,5)/6-31G(d) level[1~3] using full Breit-Pauli operator.

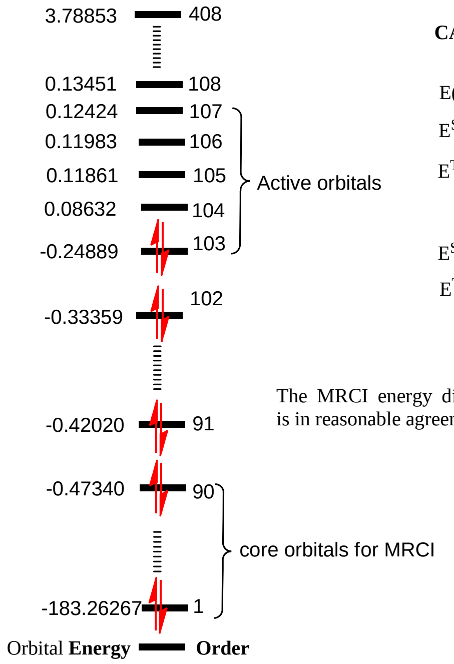

CASSCF(2, 5)/6-31G(d)

E(RHF)=-2608.65872 hartree

E^S(MCSCF)=-2608.68724 hartree (Singlet)

E^T(MCSCF)=-2608.69450 hartree (Triplet)

$\Delta E(MCSCF)=E^T-E^S=-4.6$ kcal/mol

E^S(MRCI)=-2609.04165 hartree (Singlet)

E^T(MRCI)=-2609.04357 hartree (Triplet)

$\Delta E(MRCI)=E^T-E^S=-1.2$ kcal/mol

The MRCI energy difference (1.2 kcal/mol) between the two states is in reasonable agreement with the ideal value (0.0 kcal/mol) of MECP.

SI2c. Spin-orbit coupling matrix (in cm^{-1}) using full Breit-Pauli operator

State	Sym	Spin	/ Nr.	1	2	3	4
1	1	|0 0>		422.502	-28.565	0.000	0.000
				0.000	0.000	2.016	-25.863
1	1	|1 1>+		-28.565	0.000	0.000	0.000
				0.000	0.000	0.000	-0.000
1	1	|1 0>		0.000	0.000	0.000	0.000
				-2.016	0.000	0.000	-0.000
1	1	|1 1>-		0.000	0.000	0.000	0.000
				25.863	-0.000	0.000	0.000

RMS SOC = $SQRT[(-28.565)^2 + 2.016^2 + 25.863^2]=38.6$ cm^{-1}

SI2d: References

1 References for CASSCF (a) H.-J. Werner and P. J. Knowles, *J. Chem. Phys.* 1985, **82**, 5053;(b) P. J. Knowles and H.-J. Werner, *Chem. Phys. Lett.* 1985,**115**, 259; (c) H.-J. Werner and W. Meyer, *J. Chem. Phys*. 1980, **73**, 2342; (d) H.-J. Werner and W. Meyer, *J. Chem. Phys*. 1981, **74**, 5794;(e) H.-J. Werner, *Adv. Chem. Phys.* LXIX, 1987, 1.

2 References for MRCI (Multireference internally contracted CI) (a) H.-J. Werner and P. J. Knowles, *J. Chem. Phys*. 1988， **89**, 5803; (b) P. J. Knowles and H.-J. Werner, *Chem. Phys. Lett*. 1988， **145**, 514; (c) H.-J. Werner and E.A. Reinsch, *J. Chem. Phys*. 1982, **76**, 3144; (d) H.-J. Werner, *Adv. Chem. Phys.* LXIX, 1987, 1.

3 References for spin-orbit coupling calculations: A. Berning, M. Schweizer, H.-J. Werner, P. J. Knowles and P. Palmieri, *Mol. Phys*. 2000, **98**, 1823.

SI3: Cartesian Coordinates in Å, SCF Energies and Free Energies (in a.u.) at 298.15 K and 1 atm for the Optimized Structures [BS1=6-31G(d,p), BS2=6-311++G(d,p)]

MeCCMe

B3LYP/BS1 SCF energy: -155.987341 a.u.
M06-L/BS2 SCF energy in solution: -155.999089 a.u.
M06-L/BS2 Free energy in solution: -155.941921 a.u.

C	0.000000	0.000000	0.604691
C	0.000000	0.000000	-0.604691
C	0.000000	0.000000	-2.065654
H	0.000000	1.020906	-2.464047
H	-0.884130	-0.510453	-2.464047
H	0.884130	-0.510453	-2.464047
C	0.000000	0.000000	2.065654
H	0.000000	1.020906	2.464047
H	0.884130	-0.510453	2.464047
H	-0.884130	-0.510453	2.464047

PhNNPh

B3LYP/BS1 SCF energy: -572.777801 a.u.
M06-L/BS2 SCF energy in solution: -572.826989 a.u.
M06-L/BS2 Free energy in solution: -572.674458 a.u.

N	-0.384637	0.499329	0.000169
N	0.384637	-0.499330	0.000169
C	-1.768039	0.184809	0.000083
C	-4.534505	-0.200704	-0.000111
C	-2.633534	1.286502	0.000051
C	-2.293557	-1.118939	-0.000016
C	-3.670748	-1.303512	-0.000117
C	-4.013832	1.094117	-0.000040
H	-2.198311	2.280692	0.000143
H	-1.607757	-1.958080	-0.000032
H	-4.079673	-2.309876	-0.000156
H	-4.680939	1.950856	-0.000084
H	-5.609646	-0.354535	-0.000156
C	1.768039	-0.184809	0.000083
C	4.534505	0.200704	-0.000111
C	2.633534	-1.286502	0.000051
C	2.293557	1.118939	-0.000016
C	3.670748	1.303512	-0.000117
C	4.013832	-1.094117	-0.000040
H	2.198311	-2.280692	0.000143
H	1.607756	1.958080	-0.000032
H	4.079673	2.309876	-0.000156
H	4.680939	-1.950856	-0.000084
H	5.609646	0.354535	-0.000156

Pyridine (Py)

B3LYP/BS1 SCF energy: -248.292597 a.u.
M06-L/BS2 SCF energy in solution: -248.314061 a.u.
M06-L/BS2 Free energy in solution: -248.252587 a.u.

H	-0.004868	2.440327	0.000000
C	0.000000	1.351495	0.000000
C	-0.071489	-1.372626	0.000000
C	1.209075	0.653811	0.000000
C	1.171338	-0.740186	0.000000
C	-1.220652	-0.580166	0.000000
H	2.151838	1.192119	0.000000
H	2.089529	-1.320411	0.000000
H	-2.206129	-1.043211	0.000000
H	-0.152975	-2.455184	0.000000
N	-1.201003	0.758914	0.000000

Pyrrole
B3LYP/BS1 SCF energy: -598.514016 a.u.
M06-L/BS2 SCF energy in solution: -598.556366 a.u.
M06-L/BS2 Free energy in solution: -598.325246 a.u.

C	1.260430	0.000058	-0.000053
C	4.059156	0.000056	0.000187
C	1.963843	0.537727	1.084093
C	1.964043	-0.537644	-1.084092
C	3.358824	-0.542886	-1.078556
C	3.358660	0.542957	1.078801
H	1.409910	0.938439	1.926885
H	1.410204	-0.938366	-1.926941
H	3.898144	-0.962827	-1.922502
H	3.897801	0.962894	1.922864
H	5.145028	0.000037	0.000262
C	-2.284561	0.714437	-0.072880
N	-0.165256	0.000034	-0.000187
C	-0.970435	1.132980	-0.111864
C	-0.386018	2.499075	-0.287985
H	-1.178491	3.215466	-0.515104
H	0.336584	2.532293	-1.112046
H	0.134944	2.861298	0.607062
C	-3.515036	1.568333	-0.167002
H	-3.270280	2.625050	-0.305784
H	-4.131405	1.495521	0.738502
H	-4.154679	1.268955	-1.007106
C	-0.970368	-1.133007	0.111730
C	-2.284502	-0.714500	0.072999
C	-0.385726	-2.499031	0.287527
H	0.337575	-2.532100	1.110973
H	0.134497	-2.861360	-0.607914
H	-1.177942	-3.215466	0.515445
C	-3.514863	-1.568528	0.167305
H	-4.154435	-1.269219	1.007491
H	-3.269954	-2.625216	0.306045
H	-4.131395	-1.495811	-0.738102

1cat
B3LYP/BS1 SCF energy: -2801.368573 a.u.
M06-L/BS2 SCF energy in solution: -2801.524984 a.u.
M06-L/BS2 Free energy in solution: -2801.220043 a.u.

Ti	0.021905	0.000082	0.000029
N	-0.193307	-2.256363	0.055178
C	-0.299353	-5.044581	0.084534
C	-0.949865	-2.907165	0.957798
C	0.516824	-2.982637	-0.830072
C	0.491053	-4.372886	-0.846980
C	-1.032850	-4.295840	1.002251
H	-1.479770	-2.282320	1.666980
H	1.107616	-2.415475	-1.539070
H	1.084106	-4.910259	-1.578740
H	-1.658143	-4.771251	1.750035
H	-0.339323	-6.129694	0.096830
N	-0.191033	2.256609	-0.055341
C	-0.294784	5.044921	-0.084725
C	0.520178	2.982318	0.829502
C	-0.947515	2.908030	-0.957592
C	-1.029378	4.296762	-1.002037
C	0.495574	4.372595	0.846371
H	1.110895	2.414697	1.538188
H	-1.478291	2.283610	-1.666495
H	-1.654663	4.772675	-1.749510
H	1.089488	4.909493	1.577779
H	-0.333850	6.130066	-0.097050
N	-2.563198	0.001113	-0.000008
C	-5.366450	0.001272	0.000124
C	-3.260602	0.594531	0.986186
C	-3.260778	-0.592233	-0.986136
C	-4.653416	-0.623058	-1.021307
C	-4.653240	0.625521	1.021485
H	-2.666894	1.036691	1.778669
H	-2.667194	-1.034437	-1.778693
H	-5.159525	-1.123322	-1.840308
H	-5.159209	1.125853	1.840529
H	-6.452658	0.001325	0.000182

N	1.708234	-0.000697	0.000140
C	3.081465	-0.001200	0.000168
C	5.898661	-0.002205	0.000076
C	3.802334	0.208528	-1.197132
C	3.802244	-0.211439	1.197424
C	5.194196	-0.211122	1.189344
C	5.194287	0.207215	-1.189142
H	3.245789	0.368760	-2.115182
H	3.245635	-0.371247	2.115510
H	5.734647	-0.374897	2.118058
H	5.734785	0.370608	-2.117898
H	6.984742	-0.002595	0.000054
Cl	-0.340901	0.087201	2.384382
Cl	-0.340656	-0.086534	-2.384448

2cat

B3LYP/BS1 SCF energy: -2553.064327 a.u.

M06-L/BS2 SCF energy in solution: -2553.186039 a.u.

M06-L/BS2 Free energy in solution: -2552.963437 a.u.

Ti	-0.000024	-0.735994	-0.000002
N	2.250644	-0.940511	0.102261
C	5.028932	-1.170447	-0.004081
C	2.882602	-1.949018	0.730233
C	2.991718	-0.041704	-0.574349
C	4.377662	-0.120965	-0.650395
C	4.265281	-2.099988	0.698538
H	2.249110	-2.635678	1.280018
H	2.442026	0.755614	-1.060287
H	4.927870	0.630355	-1.205947
H	4.725133	-2.931609	1.220899
H	6.110238	-1.259866	-0.045180
N	-2.250703	-0.940413	-0.102262
C	-5.029001	-1.170230	0.004085
C	-2.991740	-0.041558	0.574324
C	-2.882702	-1.948910	-0.730208
C	-4.265387	-2.099823	-0.698507
C	-4.377688	-0.120758	0.650369
H	-2.442016	0.755752	1.060239
H	-2.249239	-2.635613	-1.279973
H	-4.725273	-2.931440	-1.220844
H	-4.927866	0.630602	1.205897
H	-6.110311	-1.259603	0.045184
N	0.000013	0.953145	0.000001
C	0.000049	2.329869	0.000003
C	0.000160	5.148022	0.000006
C	-0.045858	3.051711	-1.213560
C	0.046027	3.051706	1.213568
C	0.046117	4.443776	1.206446
C	-0.045845	4.443781	-1.206436
H	-0.085833	2.497193	-2.146114
H	0.085971	2.497182	2.146120
H	0.083347	4.983626	2.148812
H	-0.083030	4.983636	-2.148801
H	0.000201	6.233874	0.000007
Cl	-0.151913	-1.701782	2.119523
Cl	0.151828	-1.701776	-2.119534

2cat'

B3LYP/BS1 SCF energy: -2553.042991 a.u.

M06-L/BS2 SCF energy in solution: -2553.171296 a.u.

M06-L/BS2 Free energy in solution: -2552.949921 a.u.

Ti	-0.067531	-0.869134	-0.075704
N	-0.209716	1.333810	-0.051922
C	-0.177023	4.119065	-0.127020
C	-0.916917	2.004792	-0.981466
C	0.523051	2.036555	0.834913
C	0.551945	3.426116	0.839128
C	-0.917734	3.393235	-1.058370
H	-1.487834	1.399181	-1.676092
H	1.106750	1.453209	1.537720
H	1.149793	3.947023	1.578516
H	-1.492870	3.887377	-1.833402
H	-0.162812	5.204104	-0.157007
N	-2.586560	-0.495565	0.034856
C	-5.349240	-0.186476	0.369923
C	-3.448336	-0.861879	-0.930017

C	-3.098301	0.016173	1.168927
C	-4.462746	0.192612	1.377322
C	-4.830049	-0.726355	-0.804128
H	-2.998452	-1.284419	-1.822623
H	-2.382018	0.271113	1.942864
H	-4.816154	0.609310	2.314425
H	-5.477124	-1.045349	-1.614273
H	-6.420826	-0.069059	0.501150
N	1.615525	-0.752873	-0.068156
C	2.975048	-0.563058	-0.027544
C	5.758847	-0.147152	0.069599
C	3.671312	-0.627085	1.200521
C	3.704572	-0.294120	-1.206728
C	5.079792	-0.088074	-1.151012
C	5.047156	-0.420066	1.241159
H	3.112102	-0.855028	2.102740
H	3.170614	-0.260954	-2.151021
H	5.627777	0.116198	-2.066939
H	5.569250	-0.476576	2.192612
H	6.832360	0.012481	0.106423
Cl	-0.499464	-1.753144	-2.174183
Cl	-0.456294	-1.895737	1.963342

TS1

B3LYP/BS1 SCF energy: -2709.024009 a.u.

M06-L/BS2 SCF energy in solution: -2709.169739 a.u.

M06-L/BS2 Free energy in solution: -2708.864878 a.u.

Ti	0.092629	-0.471199	-0.089374
N	0.481960	1.852025	-0.012737
C	0.703622	4.636603	0.072800
C	-0.137079	2.624080	-0.925252
C	1.206576	2.458874	0.944471
C	1.349813	3.841495	1.017044
C	-0.057473	4.013578	-0.914187
H	-0.693067	2.095816	-1.690638
H	1.661689	1.803371	1.677297
H	1.951698	4.277243	1.807191
H	-0.581948	4.586658	-1.671034
H	0.789728	5.718674	0.106395
N	2.622343	-0.508773	0.030450
C	5.421621	-0.659302	0.129844
C	3.381787	-0.071045	-0.991438
C	3.257261	-1.021034	1.101980
C	4.644012	-1.119868	1.190255
C	4.774207	-0.121594	-0.980077
H	2.838092	0.314827	-1.846183
H	2.616255	-1.338865	1.917112
H	5.095496	-1.547062	2.079520
H	5.330298	0.251930	-1.833489
H	6.505551	-0.717927	0.167882
C	-2.994861	-0.212491	0.038710
C	-5.721983	0.363582	0.346239
C	-3.564054	-0.131382	1.326641
C	-3.810018	0.000626	-1.091622
C	-5.163527	0.288264	-0.932739
C	-4.918842	0.155389	1.471713
H	-2.920260	-0.284734	2.186829
H	-3.357507	-0.058503	-2.076651
H	-5.785352	0.454277	-1.807874
H	-5.351134	0.220130	2.466220
H	-6.778012	0.587226	0.465870
Cl	0.475772	-0.233357	-2.458698
Cl	0.311192	-0.399134	2.315446
C	0.148775	-2.560775	-0.198198
N	-1.642554	-0.454370	-0.110647
C	-1.120044	-2.436773	-0.208183
C	-2.438712	-3.092757	-0.283495
H	-2.269462	-4.172849	-0.351570
H	-3.004449	-2.770775	-1.162158
H	-3.048833	-2.890123	0.601076
C	1.193288	-3.618232	-0.288429
H	1.837360	-3.599560	0.596034
H	1.831291	-3.436415	-1.158974
H	0.757872	-4.620213	-0.377270

IM1

B3LYP/BS1 SCF energy: -2709.046318 a.u.

M06-L/BS2 SCF energy in solution: -2709.192906 a.u.

M06-L/BS2 Free energy in solution:
-2708.889254 a.u.

Ti	-0.131509	-0.421499	0.194915
N	-0.746885	1.949436	0.087523
C	-1.190411	4.707129	-0.082655
C	-0.300116	2.785383	1.042686
C	-1.413226	2.482721	-0.952791
C	-1.666658	3.846231	-1.069779
C	-0.488845	4.164163	0.991127
H	0.201125	2.317401	1.882501
H	-1.729736	1.785040	-1.718742
H	-2.217704	4.218056	-1.926883
H	-0.096438	4.789521	1.785755
H	-1.362084	5.777368	-0.149622
N	-2.461160	-0.706463	-0.193874
C	-5.171491	-1.252356	-0.618915
C	-3.397689	-0.285337	0.676591
C	-2.868049	-1.401046	-1.273965
C	-4.205540	-1.697032	-1.519999
C	-4.756862	-0.530952	0.498432
H	-3.027314	0.248890	1.544017
H	-2.084736	-1.705828	-1.958474
H	-4.475108	-2.262761	-2.405341
H	-5.467780	-0.163936	1.230837
H	-6.223706	-1.464582	-0.783781
C	3.028211	-0.311722	0.010733
C	5.547538	0.733065	-0.677294
C	3.827806	-0.952269	-0.953053
C	3.502317	0.867979	0.610978
C	4.749305	1.382625	0.267421
C	5.077557	-0.432636	-1.284801
H	3.450594	-1.833823	-1.459853
H	2.885204	1.355332	1.359110
H	5.102576	2.291918	0.745613
H	5.681366	-0.936565	-2.034320
H	6.520886	1.134731	-0.941953
Cl	-0.632609	-0.081643	2.508151
Cl	0.052175	-0.156269	-2.162098
C	0.018196	-2.351208	0.387748
N	1.757687	-0.774696	0.365083
C	1.380962	-2.124584	0.475475
C	2.410256	-3.186375	0.805750
H	2.031209	-3.824005	1.608870
H	3.354865	-2.741330	1.121160
H	2.604756	-3.828108	-0.060523
C	-0.681069	-3.639616	0.659729
H	-1.568067	-3.748046	0.026561
H	-1.032070	-3.674564	1.700601
H	-0.047886	-4.520149	0.491808

IM2

B3LYP/BS1 SCF energy: -2460.741162 a.u.
M06-L/BS2 SCF energy in solution: -2460.850965 a.u.
M06-L/BS2 Free energy in solution:
-2460.630482 a.u.

Ti	-1.430246	-0.522075	0.025007
N	0.736042	-1.629948	0.069596
C	3.267321	-2.803604	0.202460
C	1.213587	-2.358864	-0.954004
C	1.509689	-1.485358	1.159542
C	2.776389	-2.050572	1.267676
C	2.468849	-2.961292	-0.928539
H	0.554736	-2.463224	-1.809791
H	1.086311	-0.904002	1.971483
H	3.357850	-1.901611	2.170932
H	2.803937	-3.542790	-1.780666
H	4.250714	-3.261100	0.254569
C	0.913556	1.661344	-0.153820
C	3.500605	2.761366	-0.141669
C	1.333017	2.488849	0.902829
C	1.811556	1.383075	-1.196454
C	3.094242	1.928506	-1.186131
C	2.614430	3.037579	0.901430
H	0.654949	2.681779	1.728487
H	1.484347	0.745350	-2.011007
H	3.775757	1.707679	-2.002658
H	2.923509	3.675949	1.724220
H	4.498726	3.188538	-0.138822
Cl	-2.043135	-1.728791	-1.799298

Cl	-1.818344	-1.481370	2.047656
C	-2.677348	0.990691	0.018737
N	-0.369194	1.084167	-0.166803
C	-1.579620	1.811637	-0.137263
C	-1.567517	3.305834	-0.344127
H	-2.575741	3.675278	-0.533989
H	-0.924702	3.565431	-1.191327
H	-1.169841	3.826652	0.532788
C	-4.118725	1.374823	0.053975
H	-4.569963	1.073478	1.007117
H	-4.678289	0.855257	-0.732905
H	-4.289874	2.452087	-0.068092

IM2’

B3LYP/BS1 SCF energy: -2460.744607 a.u.

M06-L/BS2 SCF energy in solution: -2460.856094a.u.

M06-L/BS2 Free energy in solution: -2460.634599 a.u.

Ti	-0.311823	-0.325880	-0.014111
N	-2.656947	-0.295711	0.108796
C	-5.430909	0.022814	0.262484
C	-3.440002	-0.514154	-0.964657
C	-3.249688	0.081097	1.258628
C	-4.625259	0.250228	1.377009
C	-4.823839	-0.366339	-0.929495
H	-2.928836	-0.826707	-1.868066
H	-2.589559	0.235685	2.104217
H	-5.048106	0.550678	2.329409
H	-5.405423	-0.559038	-1.824381
H	-6.508212	0.144165	0.322729
C	2.841060	0.084300	-0.014158
C	5.536168	-0.660310	0.273956
C	3.735225	0.904350	0.697875
C	3.310863	-1.123069	-0.561217
C	4.646743	-1.486312	-0.417661
C	5.071389	0.531400	0.832347
H	3.376515	1.812649	1.169188
H	2.616289	-1.754646	-1.105761
H	4.994699	-2.419649	-0.850626
H	5.749131	1.172630	1.388751
H	6.577390	-0.946951	0.384817
Cl	-0.469329	-1.707495	-1.839493
Cl	-0.275600	-1.173542	2.105108
C	-0.473655	1.610250	-0.296629
N	1.482710	0.389658	-0.154560
C	0.908286	1.640827	-0.392388
C	1.733866	2.836664	-0.804289
H	1.151217	3.473936	-1.473758
H	2.656202	2.532397	-1.303487
H	2.006889	3.441558	0.067832
C	-1.398730	2.757982	-0.550809
H	-2.436040	2.424170	-0.641300
H	-1.150394	3.297399	-1.475848
H	-1.367301	3.492716	0.265638

TS2

B3LYP/BS1 SCF energy: -2616.714624 a.u.

M06-L/BS2 SCF energy in solution: -2616.855893 a.u.

M06-L/BS2 Free energy in solution: -2616.551504 a.u.

Ti	1.258290	-0.066423	-0.383633
N	-0.169094	1.735920	-0.101053
C	-1.744943	4.020585	0.193715
C	-0.152881	2.709246	-1.032174
C	-0.967868	1.889289	0.970100
C	-1.771283	3.009558	1.153583
C	-0.918357	3.866351	-0.917226
H	0.480931	2.533385	-1.893651
H	-0.947988	1.078907	1.688607
H	-2.404380	3.078829	2.031343
H	-0.865917	4.621647	-1.693564
H	-2.359020	4.908707	0.308199
C	-1.965806	-1.161286	0.257801
C	-4.766116	-1.027145	0.018266
C	-2.782468	-1.104824	1.399473
C	-2.571749	-1.139218	-1.009612
C	-3.960552	-1.070006	-1.121337
C	-4.170319	-1.046395	1.280751

H	-2.310542	-1.123832	2.377897
H	-1.945094	-1.148187	-1.894324
H	-4.414164	-1.048818	-2.108356
H	-4.786015	-1.016044	2.175487
H	-5.846783	-0.976259	-0.076364
Cl	0.457684	-0.107287	-2.608642
Cl	3.343996	0.669619	-1.030418
C	1.577640	-1.923978	0.030211
N	-0.559013	-1.188168	0.449394
C	0.192416	-2.209721	0.022313
C	-0.359603	-3.487816	-0.567470
H	-0.364853	-3.421779	-1.660931
H	-1.382457	-3.672973	-0.233467
H	0.272970	-4.335194	-0.290154
C	2.688501	-2.907781	-0.165411
H	2.615781	-3.384561	-1.152610
H	2.677474	-3.713704	0.582933
H	3.661511	-2.412808	-0.124095
C	1.963734	0.772823	1.713768
C	1.881953	-0.418561	2.041730
C	2.336937	2.190328	1.909924
H	1.499246	2.864838	1.717165
H	3.147471	2.457006	1.225777
H	2.677681	2.351029	2.938675
C	1.820209	-1.604332	2.907520
H	1.989202	-1.293772	3.945872
H	2.578230	-2.344991	2.640734
H	0.835824	-2.076737	2.844825

IM3

B3LYP/BS1 SCF energy: -2616.795393 a.u.

M06-L/BS2 SCF energy in solution: -2616.925108 a.u.

M06-L/BS2 Free energy in solution: -2616.618296 a.u.

Ti	-0.288201	-0.239361	-0.241244
N	-2.670412	-0.783831	-0.130335
C	-5.441447	-1.154927	0.003867
C	-3.522218	0.218287	-0.418270
C	-3.194772	-1.969134	0.223130
C	-4.567407	-2.196943	0.300695
C	-4.904690	0.078764	-0.362995
H	-3.068924	1.156903	-0.716671
H	-2.479136	-2.752809	0.445020
H	-4.932212	-3.177099	0.588338
H	-5.540876	0.922087	-0.609076
H	-6.516467	-1.299425	0.054413
C	2.843157	-0.474141	0.062470
C	5.293570	-1.782882	0.445039
C	3.774575	0.018945	0.989393
C	3.149119	-1.626524	-0.673141
C	4.368155	-2.273607	-0.478126
C	4.991844	-0.633167	1.177153
H	3.527783	0.907079	1.563475
H	2.431394	-2.004396	-1.391423
H	4.595767	-3.164737	-1.055678
H	5.702454	-0.244214	1.900848
H	6.241068	-2.292220	0.592825
Cl	-0.851219	0.449176	-2.399706
Cl	0.028063	-2.451365	0.223266
C	0.533955	2.367727	0.008005
N	1.603125	0.196721	-0.104365
C	1.572401	1.536229	-0.425036
C	2.593345	2.054278	-1.416818
H	2.141620	2.102414	-2.415499
H	3.454432	1.389184	-1.472803
H	2.943739	3.057167	-1.160412
C	0.411447	3.732949	-0.646273
H	0.553678	3.666645	-1.727629
H	1.152555	4.446589	-0.262424
H	-0.574380	4.169151	-0.479368
C	-0.684579	0.748169	1.421659
C	-0.386744	2.049292	1.094417
C	-1.422896	0.314075	2.659667
H	-1.421453	-0.773941	2.762144
H	-2.472364	0.639026	2.648605
H	-0.963431	0.730468	3.565507
C	-0.990968	3.219656	1.858795
H	-1.321241	2.902701	2.849827
H	-1.867375	3.631390	1.342304
H	-0.276662	4.038673	1.985636

TS3
B3LYP/BS1 SCF energy: -2616.762200 a.u.
M06-L/BS2 SCF energy in solution: -2616.895706 a.u.
M06-L/BS2 Free energy in solution: -2616.586303 a.u.

Ti	-0.400874	0.135838	-0.700061
N	-2.437286	-0.702401	-0.077175
C	-4.887859	-1.680875	0.842730
C	-3.461065	0.132741	0.183729
C	-2.631463	-2.022430	0.104402
C	-3.835321	-2.550136	0.560668
C	-4.694778	-0.314911	0.648183
H	-3.276594	1.181135	-0.019355
H	-1.794150	-2.663166	-0.147517
H	-3.938426	-3.622777	0.683038
H	-5.486178	0.401335	0.840207
H	-5.840613	-2.061311	1.198375
C	2.717729	-0.410554	0.097219
C	5.180460	-1.697573	0.464364
C	3.783474	0.260969	0.722154
C	2.888969	-1.737294	-0.327064
C	4.116603	-2.371007	-0.138373
C	5.008190	-0.378653	0.891800
H	3.638514	1.277076	1.074039
H	2.069332	-2.243120	-0.823127
H	4.243227	-3.395239	-0.476587
H	5.827517	0.151399	1.368746
H	6.135410	-2.195538	0.602013
Cl	-1.588080	1.728041	-2.026820
Cl	-0.143840	-1.666111	-2.126781
C	0.298809	2.166841	0.603961
N	1.465223	0.226186	-0.045718
C	1.301776	1.617331	-0.221064
C	2.171395	2.406199	-1.164766
H	1.550966	2.908745	-1.914990
H	2.865773	1.749431	-1.691986
H	2.757972	3.177430	-0.649517
C	-0.089117	3.615504	0.503309
H	0.791475	4.265120	0.544293
H	-0.761965	3.906928	1.311973
H	-0.603189	3.803047	-0.445711
C	0.046074	-0.103008	1.266952
C	-0.286763	1.249887	1.517992
C	0.211393	-1.216162	2.242157
H	1.049859	-1.849960	1.929773
H	-0.676909	-1.860383	2.255838
H	0.410946	-0.872200	3.264558
C	-1.138177	1.678293	2.693249
H	-0.655838	2.466632	3.282978
H	-1.328438	0.834938	3.362103
H	-2.114350	2.061777	2.374028

IM4
B3LYP/BS1 SCF energy: -2616.790187 a.u.
M06-L/BS2 SCF energy in solution: -2616.926310 a.u.
M06-L/BS2 Free energy in solution: -2616.615041 a.u.

Ti	-0.414903	0.125068	-0.755059
N	-2.449027	-0.574414	-0.066424
C	-4.935893	-1.505324	0.800298
C	-3.520782	0.240762	-0.041920
C	-2.612205	-1.853733	0.321512
C	-3.832528	-2.356294	0.763693
C	-4.775080	-0.184085	0.387186
H	-3.352543	1.248752	-0.402787
H	-1.733602	-2.484131	0.252325
H	-3.908888	-3.396014	1.062508
H	-5.605715	0.513219	0.386821
H	-5.903094	-1.867064	1.136122
C	2.760194	-0.287502	0.218633
C	5.396397	-1.187700	0.224397
C	3.694633	0.302972	1.076879
C	3.135133	-1.322830	-0.637590
C	4.457944	-1.771095	-0.627145
C	5.013193	-0.148477	1.075990
H	3.383362	1.108105	1.735509
H	2.394660	-1.760517	-1.298136

H	4.752781	-2.576566	-1.292879
H	5.739861	0.309842	1.740227
H	6.424010	-1.538664	0.224636
Cl	-1.458239	1.685559	-2.221848
Cl	-0.121973	-1.785869	-2.078684
C	0.036256	2.000024	0.838602
N	1.396329	0.191374	0.239047
C	1.028324	1.559527	-0.109340
C	1.903856	2.387582	-0.996881
H	1.310441	3.187247	-1.445665
H	2.318842	1.790473	-1.814168
H	2.746803	2.840786	-0.457088
C	-0.530667	3.387314	0.842906
H	0.229686	4.118003	1.144165
H	-1.371940	3.480415	1.533692
H	-0.880994	3.662382	-0.157330
C	0.408389	-0.254563	1.225492
C	-0.329857	0.917295	1.634305
C	0.648341	-1.508992	2.009332
H	0.957929	-2.333999	1.361916
H	-0.272503	-1.811115	2.515985
H	1.423422	-1.383755	2.779334
C	-1.352239	0.896465	2.730681
H	-0.864603	0.927332	3.713187
H	-1.954901	-0.016365	2.700084
H	-2.033104	1.748619	2.671345

TS4

B3LYP/BS1 SCF energy: -3189.552727 a.u.

M06-L/BS2 SCF energy in solution: -3189.751478 a.u.

M06-L/BS2 Free energy in solution: -3189.264929 a.u.

Ti	-0.346779	-0.320795	0.543890
N	-2.164644	-1.432639	0.164838
C	-4.579313	-2.800221	-0.372585
C	-3.036382	-1.765002	1.156065
C	-2.524050	-1.781604	-1.101472
C	-3.700608	-2.454327	-1.399395
C	-4.227752	-2.439076	0.928214
H	-2.746864	-1.471565	2.157077
H	-1.827608	-1.503741	-1.881883
H	-3.916090	-2.700064	-2.433909
H	-4.866660	-2.675465	1.772808
H	-5.504075	-3.329278	-0.578460
C	3.610421	-0.921198	-0.421103
C	5.983601	0.480156	-0.946205
C	4.818706	-1.611666	-0.601380
C	3.602675	0.477790	-0.493385
C	4.786853	1.169721	-0.746434
C	5.993577	-0.914116	-0.875515
H	4.831911	-2.692110	-0.503647
H	2.670411	1.016468	-0.375078
H	4.763139	2.253693	-0.799291
H	6.921315	-1.461640	-1.015049
H	6.901062	1.023522	-1.151726
Cl	-0.856943	0.246571	2.769422
Cl	0.124103	0.172339	-1.692526
C	0.595688	-2.484604	0.951698
N	2.423287	-1.648386	-0.139044
C	1.607916	-1.464181	1.019155
C	2.312220	-1.001968	2.280651
H	1.620910	-0.979778	3.122069
H	2.742364	-0.002011	2.176867
H	3.134103	-1.688887	2.524038
C	-0.066808	-3.137865	2.142353
H	0.542546	-3.981089	2.498066
H	-1.050408	-3.541919	1.883601
H	-0.201149	-2.449674	2.977989
C	1.901684	-2.675502	-0.922364
C	0.807960	-3.212527	-0.288005
C	2.432606	-2.960592	-2.291951
H	2.481280	-2.039681	-2.883367
H	1.773865	-3.659299	-2.810807
H	3.437315	-3.395724	-2.282929
C	-0.008539	-4.391529	-0.730655
H	0.163837	-5.263259	-0.084821
H	0.236126	-4.696446	-1.751680
H	-1.082496	-4.182141	-0.697018
N	-0.837856	2.412554	0.240033
N	-1.647997	2.971469	-0.547038

C	0.420032	3.089792	0.323193
C	2.885401	4.371468	0.611153
C	0.922929	3.881983	-0.721207
C	1.158542	2.930475	1.503604
C	2.384527	3.582038	1.646816
C	2.153035	4.513294	-0.573709
H	0.342499	3.979536	-1.630395
H	0.749963	2.327509	2.306546
H	2.945413	3.471872	2.569890
H	2.546689	5.117772	-1.385726
H	3.842044	4.873578	0.722299
C	-2.944024	2.417131	-0.622683
C	-5.578808	1.535478	-0.896658
C	-3.644384	2.652177	-1.815339
C	-3.576524	1.763658	0.448577
C	-4.893001	1.334466	0.305548
C	-4.950191	2.191395	-1.958043
H	-3.141989	3.186755	-2.614736
H	-3.040280	1.627010	1.381221
H	-5.390007	0.845635	1.137925
H	-5.483654	2.358518	-2.888926
H	-6.605567	1.196664	-0.998876

IM5

B3LYP/BS1 SCF energy: -2591.122419 a.u.
M06-L/BS2 SCF energy in solution: -2591.252257 a.u.
M06-L/BS2 Free energy in solution:
-2591.021971 a.u.

Ti	-0.199996	-1.026349	0.596295
N	-2.238986	-0.797886	-0.279429
C	-4.728938	-0.764892	-1.530460
C	-2.334277	-0.717066	-1.622386
C	-3.373016	-0.860451	0.446331
C	-4.633256	-0.841510	-0.142663
C	-3.556362	-0.702674	-2.283138
H	-1.394524	-0.654164	-2.159815
H	-3.246454	-0.926166	1.520851
H	-5.516748	-0.888456	0.483990
H	-3.580600	-0.642361	-3.365409
H	-5.698953	-0.753024	-2.017781
C	-0.008400	1.876735	-0.065513
C	-0.727467	4.537932	0.486037
C	-1.010356	2.515651	-0.815968
C	0.641800	2.591546	0.954098
C	0.278990	3.910219	1.221808
C	-1.368388	3.831370	-0.534615
H	-1.488678	1.977710	-1.627750
H	1.424535	2.106096	1.525617
H	0.790109	4.450117	2.013967
H	-2.144548	4.311206	-1.124369
H	-1.003669	5.565997	0.698954
Cl	0.257095	-3.055585	-0.252232
Cl	-0.873321	-1.033119	2.768902
N	0.334447	0.540481	-0.411452
C	2.661069	-0.179242	-0.160125
C	3.065614	0.500090	-1.320152
C	3.584648	-0.971627	0.543174
C	4.376685	0.368833	-1.773389
H	2.349057	1.117243	-1.849604
C	4.889463	-1.093359	0.077175
H	3.266497	-1.480647	1.447636
C	5.294134	-0.424509	-1.081789
H	4.683270	0.894736	-2.673201
H	5.595481	-1.711856	0.623591
H	6.314909	-0.518698	-1.439179
N	1.363741	-0.060078	0.355176

IM6

B3LYP/BS1 SCF energy: -3087.736519 a.u.
M06-L/BS2 SCF energy in solution:
-3087.939756 a.u.
M06-L/BS2 Free energy in solution:
-3087.541642 a.u.

Ti	0.243189	0.012560	-0.004905
N	0.713582	2.270928	0.500340
C	1.190994	4.953734	1.155776
C	1.148162	2.622004	1.727502
C	0.504910	3.255215	-0.395711
C	0.734270	4.598884	-0.110331

C	1.398067	3.941851	2.091363
H	1.286074	1.808349	2.429292
H	0.148461	2.942704	-1.369010
H	0.546457	5.344142	-0.875602
H	1.746652	4.160778	3.094924
H	1.377320	5.993083	1.409717
N	0.921383	-2.193975	-0.508243
C	1.646203	-4.821139	-1.164495
C	0.824956	-3.190055	0.393765
C	1.367074	-2.507039	-1.741857
C	1.737357	-3.798110	-2.106371
C	1.179196	-4.506378	0.108445
H	0.456273	-2.909411	1.372099
H	1.415868	-1.687394	-2.448540
H	2.088326	-3.986412	-3.115299
H	1.078069	-5.262712	0.879203
H	1.928556	-5.838550	-1.418651
N	2.775386	0.128161	0.000774
C	5.576838	0.256977	0.008745
C	3.506283	-0.618070	0.848901
C	3.439474	0.938334	-0.843395
C	4.828620	1.037059	-0.869975
C	4.898425	-0.588676	0.883387
H	2.943309	-1.243122	1.531916
H	2.824520	1.508681	-1.529226
H	5.304408	1.712312	-1.573213
H	5.430262	-1.217490	1.589525
H	6.661742	0.306917	0.011802
N	-1.567083	0.589444	0.214026
C	-2.477524	1.474116	-0.384839
C	-4.339609	3.287368	-1.460165
C	-3.264189	1.112473	-1.493277
C	-2.651611	2.746004	0.191033
C	-3.569669	3.642461	-0.349661
C	-4.180670	2.018121	-2.021784
H	-3.139723	0.126823	-1.925948
H	-2.077783	3.005145	1.074479
H	-3.694924	4.619575	0.109479
H	-4.778743	1.726854	-2.881186
H	-5.061174	3.985130	-1.874467
Cl	0.572989	-0.576287	2.294888
Cl	0.528294	0.635780	-2.302366
C	-2.319401	-1.698567	0.380477
C	-2.373403	-2.985653	-0.185218
C	-3.125427	-1.408325	1.495807
C	-3.192620	-3.964250	0.371801
H	-1.785621	-3.193821	-1.073016
C	-3.942404	-2.395810	2.040728
H	-3.094151	-0.412298	1.921410
C	-3.981643	-3.678614	1.488896
H	-3.225602	-4.952497	-0.079376
H	-4.556530	-2.158602	2.905413
H	-4.626053	-4.441147	1.916065
N	-1.506685	-0.732877	-0.236047

TS5

B3LYP/BS1 SCF energy: -5182.177192 a.u.
M06-L/BS2 SCF energy in solution:
-5182.524162 a.u.
M06-L/BS2 Free energy in solution:
-5182.0398 a.u.

Ti	-2.807638	-0.058705	1.214396
Ti	1.419426	0.114866	-0.258065
Cl	-5.049902	-0.377957	1.179201
Cl	1.069780	0.071524	-2.519577
Cl	0.599385	-0.243163	1.889986
N	3.282426	0.270842	0.048139
N	2.897829	-1.049923	-0.269693
N	-2.453517	0.960681	-0.253767
N	-1.655612	-0.177071	-0.483784
C	-2.532262	2.117760	-1.022969
C	-3.326526	3.175702	-0.536307
C	-1.866155	2.251496	-2.253817
C	-3.443401	4.348890	-1.273353
H	-3.842543	3.060443	0.411705
C	-1.994385	3.437256	-2.976250
H	-1.257476	1.437247	-2.627112
C	-2.778734	4.488588	-2.496655
H	-4.060449	5.157892	-0.892591
H	-1.477821	3.532772	-3.927187
H	-2.877188	5.404950	-3.070895

C	-2.167755	-1.093637	-1.456190
C	-3.367111	-0.905364	-2.164850
C	-1.416813	-2.258851	-1.693722
C	-3.793822	-1.862290	-3.087453
H	-3.964299	-0.018141	-1.991804
C	-1.851881	-3.208309	-2.613501
H	-0.485651	-2.404134	-1.155741
C	-3.044523	-3.016622	-3.318246
H	-4.721718	-1.698506	-3.628319
H	-1.252190	-4.097316	-2.787507
H	-3.379803	-3.754064	-4.041250
C	3.639018	-1.771514	-1.217261
C	4.877306	-1.324155	-1.706427
C	3.138015	-3.016216	-1.636581
C	5.585412	-2.106784	-2.616417
H	5.268294	-0.370625	-1.370181
C	3.854508	-3.786612	-2.546981
H	2.192263	-3.366761	-1.234080
C	5.082360	-3.337965	-3.042772
H	6.540911	-1.751302	-2.992495
H	3.455202	-4.744596	-2.868347
H	5.641820	-3.943503	-3.749432
C	4.060782	0.496405	1.204043
C	4.391747	1.819643	1.542580
C	4.560274	-0.559066	1.984133
C	5.189507	2.078175	2.654129
H	4.026991	2.633677	0.925194
C	5.360915	-0.285945	3.091633
H	4.309209	-1.578939	1.716108
C	5.680116	1.029047	3.436077
H	5.435181	3.106093	2.906662
H	5.739059	-1.111038	3.689253
H	6.306518	1.234226	4.298938
N	1.225966	2.335766	-0.068584
C	0.405512	2.944024	0.809730
C	1.927974	3.096941	-0.933880
C	0.257045	4.327815	0.850700
H	-0.133470	2.299480	1.493886
C	1.833927	4.482630	-0.952100
H	2.576620	2.561581	-1.618190
C	0.980609	5.111627	-0.044091
H	-0.418962	4.767739	1.575020
H	2.418273	5.050768	-1.667517
H	0.883932	6.193135	-0.035620
N	-2.421250	-2.172304	1.754663
C	-2.986661	-3.187825	1.069841
C	-1.718133	-2.455357	2.870891
C	-2.856865	-4.513532	1.466765
H	-3.558488	-2.918985	0.190020
C	-1.548222	-3.756489	3.331548
H	-1.278899	-1.613618	3.392225
C	-2.124339	-4.805210	2.616828
H	-3.327697	-5.294641	0.880518
H	-0.971776	-3.932112	4.232752
H	-2.007695	-5.831503	2.951693
Cl	-2.467087	1.246819	3.028729

IM7

B3LYP/BS1 SCF energy: -5182.191947 a.u.
M06-L/BS2 SCF energy in solution:
-5182.5448 a.u.
M06-L/BS2 Free energy in solution:
-5182.0558 a.u.

Ti	1.998274	-1.175982	-1.104278
Ti	-0.713274	0.829104	0.384472
Cl	3.629938	-2.654892	-0.476072
Cl	-0.623238	1.789125	2.465913
Cl	-0.107969	-0.016416	-1.917745
N	-2.509117	0.972942	-0.272573
N	-2.363381	-0.047571	0.702616
N	2.337976	0.182910	0.087312
N	1.185428	-0.351073	0.711937
C	3.127325	1.258018	0.515749
C	4.256765	1.584253	-0.261767
C	2.835217	2.009814	1.667008
C	5.066689	2.653146	0.107544
H	4.483045	0.994986	-1.143243
C	3.659658	3.078070	2.019277
H	1.973592	1.762916	2.273369
C	4.774701	3.408662	1.247586
H	5.936012	2.892864	-0.498121

H	3.423188	3.651396	2.911089
H	5.413144	4.239134	1.533336
C	1.413546	-1.164473	1.880124
C	2.612460	-1.172368	2.607161
C	0.367888	-2.004156	2.290440
C	2.739982	-1.986203	3.732616
H	3.448564	-0.563519	2.291662
C	0.504215	-2.815339	3.414952
H	-0.549091	-2.027041	1.713634
C	1.691310	-2.806936	4.148925
H	3.676016	-1.978460	4.283596
H	-0.318392	-3.460487	3.711026
H	1.800972	-3.437857	5.025552
C	-3.330601	-0.108973	1.730051
C	-4.560850	0.563658	1.621764
C	-3.080907	-0.898098	2.866312
C	-5.513644	0.444497	2.632179
H	-4.761393	1.173243	0.749439
C	-4.041205	-1.008604	3.867669
H	-2.126089	-1.396305	2.974178
C	-5.264908	-0.343054	3.757396
H	-6.458999	0.971152	2.534493
H	-3.827427	-1.613630	4.744321
H	-6.011911	-0.434163	4.540205
C	-3.226712	0.699401	-1.446147
C	-3.221146	1.665773	-2.469757
C	-3.994305	-0.466764	-1.612539
C	-3.952549	1.458334	-3.634129
H	-2.635320	2.569580	-2.339852
C	-4.723716	-0.661673	-2.784955
H	-4.018803	-1.204005	-0.818123
C	-4.709433	0.294191	-3.801646
H	-3.931989	2.210575	-4.417824
H	-5.313484	-1.567332	-2.899692
H	-5.282121	0.138090	-4.710766
N	-0.213884	2.852731	-0.506099
C	0.862338	3.097454	-1.277049
C	-1.028142	3.880259	-0.190620
C	1.166880	4.370820	-1.749876
H	1.482088	2.246049	-1.527678
C	-0.796311	5.177686	-0.631046
H	-1.883600	3.633241	0.427468
C	0.324440	5.430483	-1.422987
H	2.049121	4.513348	-2.363684
H	-1.483697	5.968952	-0.352720
H	0.534074	6.434524	-1.779560
N	0.631418	-3.002057	-1.349920
C	-0.662888	-3.057413	-0.977702
C	1.147760	-4.061772	-2.007765
C	-1.468585	-4.163375	-1.225553
H	-1.068645	-2.181349	-0.488118
C	0.400160	-5.193942	-2.313563
H	2.194341	-3.995215	-2.279410
C	-0.931938	-5.252372	-1.909243
H	-2.500732	-4.153761	-0.893397
H	0.866629	-6.010963	-2.852745
H	-1.539296	-6.126223	-2.125102
Cl	2.984809	-0.490029	-3.033448

TS6

B3LYP/BS1 SCF energy: -5182.162851 a.u.
M06-L/BS2 SCF energy in solution:
-5182.525362 a.u.
M06-L/BS2 Free energy in solution:
-5182.0390 a.u.

Ti	-1.827383	0.470995	-1.301545
Ti	0.594341	-0.831973	0.425925
Cl	-3.704312	1.816549	-1.054357
Cl	0.080014	-2.001668	2.366937
Cl	0.552927	-0.274553	-1.952801
N	2.606325	-0.884127	0.413421
N	2.040959	0.192444	1.071398
N	-2.382058	-0.830457	-0.211188
N	-0.754557	0.670642	0.501987
C	-3.166091	-1.457301	0.691946
C	-4.157020	-0.727013	1.410232
C	-3.047164	-2.856866	0.922729
C	-4.983646	-1.378659	2.314423
H	-4.256345	0.333666	1.213567
C	-3.894539	-3.491760	1.816660
H	-2.293473	-3.412412	0.378021

C	-4.859480	-2.757782	2.520185
H	-5.736686	-0.814909	2.857267
H	-3.803439	-4.561645	1.978685
H	-5.514557	-3.261971	3.224690
C	-1.181602	1.506783	1.514726
C	-1.232212	1.084658	2.871665
C	-1.556243	2.850401	1.227093
C	-1.636842	1.958151	3.873192
H	-0.954381	0.067364	3.111363
C	-1.919735	3.723632	2.242395
H	-1.532227	3.193295	0.201867
C	-1.971171	3.281600	3.569621
H	-1.678668	1.608215	4.900439
H	-2.179131	4.749718	1.999051
H	-2.269836	3.964467	4.359795
C	2.712583	0.917321	2.059443
C	3.926020	0.452623	2.603070
C	2.171258	2.133793	2.514338
C	4.569226	1.190440	3.592749
H	4.340537	-0.484864	2.251382
C	2.829542	2.860721	3.501301
H	1.246426	2.498974	2.084962
C	4.030073	2.396566	4.047426
H	5.499926	0.818410	4.011872
H	2.401822	3.798419	3.844473
H	4.539079	2.968459	4.817298
C	3.628742	-0.662057	-0.521376
C	4.159970	-1.791639	-1.175673
C	4.171952	0.606503	-0.805441
C	5.198816	-1.654105	-2.089308
H	3.746745	-2.769080	-0.952222
C	5.214908	0.729888	-1.719711
H	3.769640	1.485071	-0.315860
C	5.737249	-0.393551	-2.365559
H	5.593519	-2.535368	-2.587099
H	5.622557	1.714747	-1.931112
H	6.551602	-0.288423	-3.075921
N	0.581613	-2.971800	-0.508210
C	-0.213882	-3.354890	-1.525343
C	1.400742	-3.889396	0.040855
C	-0.222316	-4.657607	-2.019489
H	-0.854680	-2.595097	-1.956751
C	1.463228	-5.205397	-0.406141
H	2.015804	-3.541632	0.862525
C	0.633259	-5.600743	-1.454952
H	-0.888250	-4.910470	-2.837220
H	2.147056	-5.900842	0.068536
H	0.654069	-6.622059	-1.823642
N	-0.930307	2.445629	-2.294668
C	0.242872	2.981904	-1.912782
C	-1.583686	3.009334	-3.326645
C	0.796305	4.099366	-2.530345
H	0.747606	2.485084	-1.091309
C	-1.091784	4.118955	-4.009275
H	-2.525487	2.548507	-3.601648
C	0.118074	4.678353	-3.602246
H	1.742315	4.496127	-2.177868
H	-1.654045	4.529735	-4.840842
H	0.526007	5.546573	-4.111500
Cl	-2.442444	-0.465988	-3.337834

IM8

B3LYP/BS1 SCF energy: -5182.224803 a.u.
M06-L/BS2 SCF energy in solution:
-5182.575551 a.u.
M06-L/BS2 Free energy in solution:
-5182.0916 a.u.

Ti	2.547341	0.783534	0.017744
Ti	-0.275740	0.450624	0.028747
Cl	3.464573	1.558791	1.969017
Cl	-0.680302	0.198517	2.366536
Cl	-0.714641	0.207150	-2.285874
N	-2.255248	-0.836712	0.061595
N	-1.830401	-2.001293	-0.071328
N	0.932684	1.861259	-0.005153
N	1.359077	-0.636931	0.031351
C	0.985586	3.261716	-0.148951
C	1.206780	4.087133	0.967947
C	0.810613	3.845228	-1.417462
C	1.240742	5.472747	0.813509
H	1.361967	3.629001	1.938353

C	0.848270	5.230933	-1.556365
H	0.657094	3.198742	-2.274637
C	1.061777	6.051268	-0.444897
H	1.414784	6.101217	1.682669
H	0.714700	5.671390	-2.540684
H	1.094983	7.130779	-0.560465
C	1.428116	-2.038009	-0.063172
C	1.285942	-2.837276	1.085395
C	1.653870	-2.654472	-1.307297
C	1.385787	-4.224653	0.988033
H	1.088230	-2.354471	2.036829
C	1.753783	-4.043225	-1.390713
H	1.739287	-2.032430	-2.192254
C	1.623351	-4.835386	-0.246001
H	1.275413	-4.831276	1.882942
H	1.937881	-4.507380	-2.356247
H	1.703920	-5.916384	-0.315674
C	-2.626689	-3.179370	-0.056545
C	-3.719464	-3.407918	0.797685
C	-2.146521	-4.208930	-0.880676
C	-4.340910	-4.653778	0.788492
H	-4.059958	-2.635257	1.475053
C	-2.805589	-5.434852	-0.912504
H	-1.261592	-4.022947	-1.479552
C	-3.901702	-5.660713	-0.076579
H	-5.174630	-4.838600	1.459206
H	-2.448024	-6.221840	-1.569376
H	-4.402156	-6.624534	-0.083924
C	-3.655712	-0.487460	0.093052
C	-4.176893	0.158667	1.217854
C	-4.439786	-0.701807	-1.045797
C	-5.514902	0.552987	1.212053
H	-3.535746	0.328112	2.075775
C	-5.770122	-0.283621	-1.042895
H	-4.003498	-1.175955	-1.918360
C	-6.312645	0.335084	0.085731
H	-5.931818	1.038913	2.089235
H	-6.382152	-0.443072	-1.925668
H	-7.351094	0.652577	0.085036
N	-1.894606	2.231334	0.064930
C	-2.590823	2.603650	-1.025718
C	-2.035915	2.969884	1.182826
C	-3.453310	3.696546	-1.035300
H	-2.438537	1.997317	-1.910854
C	-2.873258	4.079330	1.255066
H	-1.461937	2.642618	2.041634
C	-3.600084	4.451320	0.126234
H	-3.990947	3.944919	-1.944175
H	-2.943010	4.636720	2.183070
H	-4.261524	5.312555	0.149867
N	4.278354	-0.863679	0.135858
C	5.047124	-1.224790	-0.907883
C	4.462710	-1.504427	1.306359
C	6.019426	-2.217797	-0.820918
H	4.875077	-0.685376	-1.831906
C	5.409730	-2.508901	1.476914
H	3.835687	-1.184383	2.130073
C	6.206585	-2.874045	0.393445
H	6.614032	-2.462280	-1.694587
H	5.514317	-2.987806	2.444405
H	6.957755	-3.651913	0.494398
Cl	3.457549	1.445623	-1.966107

TS7

B3LYP/BS1 SCF energy: -5182.217549 a.u.

M06-L/BS2 SCF energy in solution:
-5182.556156 a.u.

M06-L/BS2 Free energy in solution:
-5182.0762 a.u.

Ti	2.983779	-0.187333	0.142472
Ti	0.311027	0.707440	-0.081396
Cl	3.926882	0.121717	2.206039
Cl	-0.633461	0.504360	2.033299
Cl	-0.286333	0.537043	-2.292058
N	-3.092180	0.039750	-0.289252
N	-3.037011	-1.174241	-0.573861
N	1.952057	1.498037	0.020026
N	1.341477	-0.982139	0.019687
C	2.545987	2.758830	-0.195388
C	3.184551	3.432225	0.860887
C	2.496247	3.354575	-1.468836

C	3.748353	4.689521	0.643803
H	3.237531	2.957132	1.834504
C	3.066952	4.608971	-1.671179
H	2.018337	2.817896	-2.281636
C	3.692028	5.283712	-0.618454
H	4.239619	5.201863	1.466379
H	3.025482	5.060068	-2.658702
H	4.136894	6.260667	-0.783285
C	0.709067	-2.236032	-0.085410
C	0.160314	-2.857649	1.050696
C	0.631241	-2.882179	-1.332706
C	-0.439958	-4.110717	0.936562
H	0.199012	-2.340803	2.003151
C	0.030624	-4.135395	-1.431271
H	1.030589	-2.382394	-2.208668
C	-0.506369	-4.756103	-0.300197
H	-0.862346	-4.581660	1.819938
H	-0.022070	-4.626509	-2.399139
H	-0.978568	-5.730585	-0.383469
C	-4.118610	-2.093831	-0.373833
C	-4.832838	-2.190315	0.829866
C	-4.317866	-3.042395	-1.385960
C	-5.766459	-3.211749	0.994241
H	-4.643777	-1.484047	1.630336
C	-5.281888	-4.035400	-1.227289
H	-3.712742	-2.980544	-2.284929
C	-6.006558	-4.125195	-0.035761
H	-6.310697	-3.292797	1.930795
H	-5.452339	-4.753212	-2.024465
H	-6.742553	-4.912816	0.096360
C	-4.302602	0.717029	0.090129
C	-4.248027	1.563086	1.206098
C	-5.445570	0.693554	-0.722077
C	-5.360702	2.333046	1.541270
H	-3.340806	1.587812	1.801450
C	-6.539172	1.493571	-0.394857
H	-5.468297	0.062544	-1.604271
C	-6.506913	2.304871	0.742189
H	-5.326918	2.967460	2.422457
H	-7.420903	1.479475	-1.029249
H	-7.365654	2.918692	0.997595
N	-0.692334	2.822733	-0.059813
C	-1.563344	3.258410	-0.988582
C	-0.388394	3.646014	0.963169
C	-2.152958	4.518620	-0.931680
H	-1.782016	2.565658	-1.791971
C	-0.926204	4.921975	1.087416
H	0.303949	3.257839	1.700235
C	-1.827111	5.369373	0.121876
H	-2.853136	4.816352	-1.704565
H	-0.639186	5.544933	1.927473
H	-2.266807	6.360024	0.191150
N	3.831359	-2.382476	0.256691
C	4.379604	-3.026425	-0.791188
C	3.665814	-3.069882	1.404232
C	4.785390	-4.356521	-0.729690
H	4.501997	-2.442681	-1.695829
C	4.042997	-4.400960	1.547075
H	3.230225	-2.521865	2.231229
C	4.615452	-5.059834	0.460752
H	5.226741	-4.821475	-1.604596
H	3.887954	-4.901980	2.496346
H	4.922956	-6.098251	0.540963
Cl	4.244663	0.112080	-1.733693

IM9

B3LYP/BS1 SCF energy: -4609.522200 a.u.

M06-L/BS2 SCF energy in solution: -4609.735999 a.u.

M06-L/BS2 Free energy in solution: -4609.4276 a.u.

Ti	-0.822505	-1.149583	-0.142101
Ti	0.822586	1.149431	0.141668
Cl	-0.791423	-2.537399	1.664692
Cl	-0.970545	-1.984352	-2.261857
Cl	0.970839	1.983488	2.261683
Cl	0.791292	2.537371	-1.665009
N	-1.076042	0.674166	0.080782
N	1.076068	-0.674349	-0.081198
C	-2.114509	1.618007	-0.052706
C	-2.750794	1.811745	-1.291963

C	-2.525378	2.374530	1.058621
C	-3.785745	2.736120	-1.407161
H	-2.411284	1.244394	-2.152114
C	-3.565281	3.294790	0.930333
H	-2.018671	2.235585	2.007572
C	-4.200504	3.479420	-0.298724
H	-4.267286	2.879774	-2.370188
H	-3.873860	3.873856	1.796046
H	-5.006112	4.200939	-0.395247
C	2.114488	-1.618124	0.053422
C	2.525623	-2.375563	-1.057167
C	2.750491	-1.810778	1.292992
C	3.565531	-3.295692	-0.927858
H	2.019125	-2.237432	-2.006351
C	3.785426	-2.735032	1.409215
H	2.410799	-1.242670	2.152573
C	4.200468	-3.479260	0.301490
H	3.874320	-3.875477	-1.793016
H	4.266754	-2.877860	2.372471
H	5.006090	-4.200656	0.398809
N	-3.167683	-1.346924	0.034979
C	-3.718837	-0.973516	1.206492
C	-3.985009	-1.803379	-0.931293
C	-5.084739	-1.042569	1.453000
H	-3.033436	-0.617041	1.966201
C	-5.363121	-1.907402	-0.763767
H	-3.506417	-2.094725	-1.858802
C	-5.926109	-1.520011	0.449564
H	-5.472371	-0.727576	2.415376
H	-5.973339	-2.286719	-1.576038
H	-6.997525	-1.588921	0.611263
N	3.167723	1.346752	-0.035682
C	3.985088	1.804722	0.929838
C	3.718718	0.972413	-1.206975
C	5.363087	1.909367	0.761754
H	3.506654	2.096723	1.857218
C	5.084487	1.042068	-1.454024
H	3.033307	0.614595	-1.966036
C	5.925897	1.521074	-0.451375
H	5.973357	2.289832	1.573447
H	5.471995	0.726324	-2.416203
H	6.997216	1.590474	-0.613510

IM10

B3LYP/BS1 SCF energy: -5106.124883 a.u.

M06-L/BS2 SCF energy in solution:
-5106.417935 a.u.

M06-L/BS2 Free energy in solution:
-5105.9436 a.u.

Ti	-1.422427	-0.073010	0.037567
Ti	1.422322	0.073144	0.037612
Cl	-1.869566	0.010376	2.360056
Cl	-1.858817	-0.075911	-2.285240
Cl	1.869354	-0.010263	2.360138
Cl	1.858691	0.076210	-2.285239
N	-0.020039	1.267308	0.033956
N	0.019963	-1.267186	0.033871
C	-0.227696	2.653416	-0.061415
C	0.047609	3.331004	-1.265026
C	-0.711235	3.378481	1.044357
C	-0.158072	4.707186	-1.350995
H	0.418190	2.761578	-2.110607
C	-0.906012	4.755584	0.943139
H	-0.928462	2.844623	1.963600
C	-0.633550	5.426896	-0.251193
H	0.057571	5.220064	-2.284523
H	-1.276969	5.305289	1.804056
H	-0.789472	6.499249	-0.324607
C	0.227640	-2.653297	-0.061416
C	-0.047600	-3.330940	-1.265010
C	0.711128	-3.378316	1.044411
C	0.158090	-4.707127	-1.350914
H	-0.418148	-2.761558	-2.110635
C	0.905913	-4.755421	0.943258
H	0.928309	-2.844420	1.963643
C	0.633516	-5.426787	-0.251060
H	-0.057505	-5.220042	-2.284432
H	1.276830	-5.305089	1.804215
H	0.789448	-6.499143	-0.324415
N	-3.436099	1.334743	-0.022282
C	-4.454013	1.168787	0.842372

C	-3.543902	2.316547	-0.936811
C	-5.604251	1.952681	0.817852
H	-4.319546	0.391738	1.584975
C	-4.648220	3.160430	-1.014872
H	-2.716464	2.418286	-1.627797
C	-5.703877	2.972963	-0.125774
H	-6.396143	1.764769	1.534980
H	-4.670517	3.943289	-1.765025
H	-6.583440	3.608881	-0.165617
N	-2.969031	-1.924157	0.108710
C	-3.912412	-2.130008	-0.829597
C	-2.872035	-2.818479	1.111265
C	-4.788204	-3.211711	-0.795348
H	-3.942033	-1.404192	-1.633675
C	-3.696722	-3.935059	1.209487
H	-2.113775	-2.615383	1.857000
C	-4.677884	-4.135976	0.241300
H	-5.533251	-3.321952	-1.575956
H	-3.564783	-4.625982	2.034885
H	-5.340635	-4.994854	0.292438
N	3.435941	-1.334761	-0.022330
C	3.543871	-2.316343	-0.937080
C	4.453726	-1.169021	0.842518
C	4.648201	-3.160206	-1.015191
H	2.716522	-2.417907	-1.628198
C	5.603965	-1.952914	0.817976
H	4.319128	-0.392163	1.585298
C	5.703729	-2.972958	-0.125892
H	4.670612	-3.942879	-1.765534
H	6.395748	-1.765189	1.535274
H	6.583297	-3.608868	-0.165765
N	2.969198	1.923987	0.108855
C	3.912709	2.129510	-0.829398
C	2.872304	2.818446	1.111291
C	4.788734	3.211022	-0.795201
H	3.942198	1.403581	-1.633377
C	3.697228	3.934858	1.209450
H	2.113928	2.615600	1.856979
C	4.678520	4.135438	0.241325
H	5.533877	3.321010	-1.575754
H	3.565368	4.625913	2.034750
H	5.341454	4.994178	0.292416

TS8

B3LYP/BS1 SCF energy: -5106.077342 a.u.

M06-L/BS2 SCF energy in solution:
-5106.362600 a.u.

M06-L/BS2 Free energy in solution:
-5105.8966 a.u.

Ti	-2.061689	-0.416998	-0.005435
Ti	2.061667	0.417734	-0.004373
Cl	-1.989487	0.158892	2.255271
Cl	-2.037592	0.313076	-2.227483
Cl	1.991205	-0.155656	2.257247
Cl	2.036805	-0.312096	-2.226660
N	0.948050	1.689437	-0.064537
N	-0.947518	-1.688281	-0.065316
C	0.225603	2.855187	-0.158845
C	0.016145	3.470144	-1.413990
C	-0.312540	3.464581	0.997331
C	-0.692071	4.665633	-1.499547
H	0.406859	2.980803	-2.299725
C	-1.020632	4.659621	0.894975
H	-0.176067	2.971688	1.954097
C	-1.211003	5.268925	-0.348960
H	-0.841901	5.129833	-2.470914
H	-1.429191	5.116724	1.792292
H	-1.759072	6.204133	-0.421622
C	-0.225308	-2.854207	-0.159240
C	-0.016206	-3.469815	-1.414127
C	0.312830	-3.463244	0.997138
C	0.691611	-4.665573	-1.499225
H	-0.406901	-2.980764	-2.300032
C	1.020521	-4.658565	0.895242
H	0.176650	-2.969885	1.953713
C	1.210510	-5.268510	-0.348433
H	0.841149	-5.130276	-2.470397
H	1.429058	-5.115380	1.792716
H	1.758259	-6.203933	-0.420743
N	-3.985821	1.253548	0.113681
C	-4.941345	1.103291	1.048054

C	-4.007793	2.374549	-0.627086
C	-5.954016	2.033288	1.260405
H	-4.874114	0.208811	1.657614
C	-4.972356	3.368924	-0.468070
H	-3.223780	2.464880	-1.370378
C	-5.969228	3.194008	0.487393
H	-6.703048	1.851763	2.023981
H	-4.929967	4.258927	-1.086493
H	-6.738730	3.946696	0.632696
N	-3.728031	-1.835346	-0.023852
C	-4.604328	-1.882946	-1.046337
C	-3.835898	-2.738833	0.971196
C	-5.616747	-2.834829	-1.108849
H	-4.465284	-1.139900	-1.823640
C	-4.828876	-3.711330	0.982343
H	-3.099831	-2.660921	1.763270
C	-5.734848	-3.764002	-0.076793
H	-6.294554	-2.839623	-1.955218
H	-4.878764	-4.415172	1.805599
H	-6.515703	-4.517994	-0.098504
N	3.983919	-1.254908	0.114214
C	4.005296	-2.375484	-0.627213
C	4.939866	-1.105459	1.048297
C	4.969644	-3.370220	-0.469160
H	3.220967	-2.465175	-1.370244
C	5.952381	-2.035854	1.259674
H	4.873078	-0.211346	1.658429
C	5.966968	-3.196125	0.485981
H	4.926736	-4.259859	-1.088070
H	6.701780	-1.854967	2.023042
H	6.736327	-3.949106	0.630527
N	3.729072	1.834867	-0.024073
C	4.604238	1.882403	-1.047529
C	3.838758	2.737719	0.971365
C	5.617308	2.833559	-1.110643
H	4.463733	1.139920	-1.825106
C	4.832439	3.709503	0.981894
H	3.103618	2.659811	1.764301
C	5.737258	3.762089	-0.078229
H	6.294170	2.838299	-1.957769
H	4.883771	4.412854	1.805481
H	6.518641	4.515520	-0.100411

TS9

B3LYP/BS1 SCF energy: -2553.011459 a.u.

M06-L/BS2 SCF energy in solution:
-2553.166857 a.u.

M06-L/BS2 Free energy in solution:
-2552.9430 a.u.

Ti	-0.083153	-0.376342	-0.040593
N	-0.238220	1.804605	-0.073265
C	-0.330489	4.590076	-0.104584
C	-0.710688	2.464373	-1.151761
C	0.191809	2.518693	0.989555
C	0.159509	3.907736	1.009286
C	-0.772232	3.853316	-1.201986
H	-1.026427	1.844954	-1.984020
H	0.557252	1.941134	1.830951
H	0.516798	4.437327	1.885511
H	-1.158380	4.338265	-2.091742
H	-0.364666	5.675290	-0.116928
N	-2.493341	-1.001621	0.022492
C	-5.201579	-1.694063	0.188420
C	-3.014797	-1.962209	-0.761829
C	-3.317903	-0.386729	0.890745
C	-4.673147	-0.687317	0.995109
C	-4.352100	-2.347965	-0.701376
H	-2.332277	-2.417281	-1.470650
H	-2.860198	0.353547	1.537165
H	-5.291232	-0.148031	1.705080
H	-4.711834	-3.141043	-1.348230
H	-6.251660	-1.963752	0.253771
N	1.587028	-0.522678	-0.040499
C	2.940521	-0.772100	-0.007379
C	5.707799	-1.262704	0.067977
C	3.565771	-1.164467	1.196050
C	3.724461	-0.632479	-1.172652
C	5.094510	-0.875913	-1.127464
C	4.936476	-1.406123	1.225404
H	2.954734	-1.273462	2.086533
H	3.235814	-0.340349	-2.096659

H	5.687992	-0.766473	-2.031340
H	5.406943	-1.709239	2.157060
H	6.776807	-1.452961	0.096780
Cl	-0.466762	-0.769331	-2.345799
Cl	-0.414774	-0.637333	2.293893

TS10

B3LYP/BS1 SCF energy: -2772.763253 a.u.
M06-L/BS2 SCF energy in solution: -2772.914225 a.u.
M06-L/BS2 Free energy in solution: -2772.526055 a.u.

Ti	-0.685008	0.589067	0.267789
N	-2.472092	-0.490184	-0.084255
C	-4.893283	-1.840035	-0.644867
C	-3.494774	-0.534047	0.817709
C	-2.682508	-1.120844	-1.276857
C	-3.857538	-1.791994	-1.580708
C	-4.692860	-1.189067	0.574413
H	-3.319215	-0.017735	1.753998
H	-1.865909	-1.070264	-1.986953
H	-3.950391	-2.272610	-2.549150
H	-5.458090	-1.184220	1.343714
H	-5.820610	-2.360553	-0.858670
C	3.330775	-0.258034	-0.049196
C	5.794806	1.020404	-0.469365
C	4.524767	-0.994811	-0.040635
C	3.384003	1.126632	-0.260437
C	4.610300	1.758591	-0.459667
C	5.744972	-0.359302	-0.263208
H	4.491938	-2.059415	0.164280
H	2.461585	1.693135	-0.295029
H	4.636284	2.831927	-0.623671
H	6.660853	-0.943122	-0.255088
H	6.747152	1.515399	-0.632984
Cl	-1.199925	1.727371	2.225378
Cl	0.168895	0.769080	-1.892106
C	0.108158	-1.500187	1.156350
N	2.095110	-0.911675	0.194479
C	1.156932	-0.520514	1.196463
C	1.725157	0.104317	2.455731
H	0.942545	0.255901	3.196936
H	2.195945	1.073610	2.273803
H	2.489086	-0.557494	2.885474
C	-0.749232	-1.900637	2.333599
H	-0.243191	-2.676095	2.926272
H	-1.707522	-2.318605	2.010968
H	-0.961052	-1.063054	3.001223
C	1.618569	-2.030446	-0.482227
C	0.428650	-2.424712	0.084349
C	2.291834	-2.553097	-1.712268
H	2.547097	-1.731898	-2.390003
H	1.622829	-3.233018	-2.243489
H	3.215838	-3.101148	-1.498639
C	-0.374139	-3.644463	-0.267731
H	-0.191879	-4.464805	0.440196
H	-0.126863	-4.022789	-1.263873
H	-1.449160	-3.444522	-0.249285
C	-1.488454	3.366918	-0.645867
C	-2.558429	2.853827	-0.900504
C	-0.246727	4.093962	-0.387292
H	-0.360028	5.149930	-0.657209
H	0.023917	4.044569	0.672477
H	0.575641	3.682013	-0.981665
C	-3.881165	2.345215	-1.253879
H	-3.816506	1.533673	-1.986048
H	-4.413972	1.964670	-0.376511
H	-4.488654	3.144352	-1.693071

IM11

B3LYP/BS1 SCF energy: -2174.315285 a.u.
M06-L/BS2 SCF energy in solution: -2174.393315 a.u.
M06-L/BS2 Free energy in solution: -2174.262743 a.u.

Ti	-0.885601	-0.415979	0.000669
N	1.330195	-0.083966	0.002671
C	4.095421	0.310188	0.002675
C	2.013064	0.027352	1.160345
C	2.018353	-0.009612	-1.154846

C	3.394157	0.187868	-1.195718
C	3.388661	0.226328	1.201161
H	1.427798	-0.055141	2.069601
H	1.437197	-0.120504	-2.063740
H	3.898390	0.239197	-2.154332
H	3.888488	0.308479	2.159940
H	5.169889	0.464552	0.002667
Cl	-1.079072	-1.483085	1.999810
Cl	-1.085976	-1.516732	-1.978679
C	-3.717597	1.358397	0.010435
H	-4.208288	0.925802	0.889289
H	-3.889257	2.442244	0.016804
H	-4.218717	0.935555	-0.867459
C	-0.458821	2.932141	-0.046558
H	0.122399	3.075130	-0.965489
H	-1.202744	3.736800	0.009784
H	0.241980	3.059268	0.786946
C	-2.266964	1.011304	-0.001789
C	-1.081186	1.576834	-0.021813

TS11

B3LYP/BS1 SCF energy: -2330.289206 a.u.

M06-L/BS2 SCF energy in solution: -2330.390868 a.u.

M06-L/BS2 Free energy in solution: -2330.176019 a.u.

Ti	0.523793	-0.226811	0.011973
N	-1.865216	-0.172025	0.049844
C	-4.653680	0.074654	0.172741
C	-2.667398	-0.992399	-0.652572
C	-2.450697	0.772049	0.813979
C	-3.829104	0.933787	0.897791
C	-4.057607	-0.907283	-0.613222
H	-2.163711	-1.731053	-1.264439
H	-1.781464	1.403008	1.386680
H	-4.239423	1.715277	1.528067
H	-4.650885	-1.604117	-1.195369
H	-5.734351	0.168835	0.221701
Cl	0.274080	-1.828348	-1.680902
Cl	0.196864	-0.247644	2.331864

C	2.869946	-0.082109	-0.060874
C	2.410036	-1.175197	0.358044
C	2.607814	-2.576199	0.795710
H	2.109885	-3.257463	0.099842
H	2.169152	-2.726542	1.787149
H	3.673201	-2.830481	0.839126
C	4.059782	0.634705	-0.584899
H	4.444098	1.382620	0.112312
H	3.853283	1.127899	-1.538596
H	4.847679	-0.107969	-0.749114
C	0.348489	1.536834	-0.948172
C	1.416219	1.673909	-0.209906
C	-0.581880	2.414092	-1.715633
H	-1.629980	2.154633	-1.543408
H	-0.397754	2.272635	-2.787547
H	-0.440900	3.479238	-1.489381
C	2.228694	2.806071	0.330798
H	2.728855	2.554377	1.269580
H	1.552874	3.648412	0.522177
H	2.982782	3.159060	-0.380147

IM12

B3LYP/BS1 SCF energy: -2330.330785 a.u.

M06-L/BS2 SCF energy in solution: -2330.420565 a.u.

M06-L/BS2 Free energy in solution: -2330.205642 a.u.

Ti	-0.332719	-0.586018	-0.001180
N	2.098160	-0.072755	0.005030
C	4.810677	0.616586	0.025174
C	2.739735	0.192322	1.158119
C	2.805330	-0.000314	-1.137610
C	4.155314	0.338874	-1.172685
C	4.086443	0.540046	1.213395
H	2.146868	0.106693	2.062309
H	2.263461	-0.235875	-2.047164
H	4.674673	0.378044	-2.124089
H	4.550801	0.740326	2.172923
H	5.863378	0.882655	0.033183
Cl	-0.003720	-1.502134	2.055610

Cl	-0.001789	-1.604876	-2.007105
C	-2.947950	0.444677	-0.000577
C	-2.370380	-0.790137	0.016312
C	-3.069070	-2.117553	0.059154
H	-2.761175	-2.679994	0.949661
H	-2.781399	-2.726481	-0.807160
H	-4.162728	-2.048469	0.070033
C	-4.442188	0.687148	0.020578
H	-4.768978	1.258763	-0.856163
H	-4.742631	1.264135	0.903485
H	-5.003108	-0.247143	0.032126
C	-0.684491	1.432899	-0.060810
C	-2.040484	1.633153	-0.041284
C	0.351292	2.528332	-0.122058
H	1.027011	2.388426	-0.973153
H	0.982143	2.533028	0.774813
H	-0.082155	3.529070	-0.217560
C	-2.697742	2.997409	-0.060138
H	-3.338954	3.112202	-0.942053
H	-1.976371	3.813391	-0.065835
H	-3.343410	3.131421	0.815387

TS12

B3LYP/BS1 SCF energy: -2486.301533 a.u.

M06-L/BS2 SCF energy in solution: -2486.421423 a.u.

M06-L/BS2 Free energy in solution: -2486.123572 a.u.

Ti	-0.199215	0.438047	-0.476228
C	-0.827735	1.039699	1.820850
C	-2.216809	0.724607	-0.408200
C	-2.893688	-0.458788	-0.156956
C	-0.312247	-0.073062	2.030451
C	-2.103112	-1.639930	0.107906
C	-0.718033	-1.509600	0.165759
C	-2.911000	2.012036	-0.777965
H	-3.277672	1.954860	-1.811962
H	-3.777006	2.231973	-0.139124
H	-2.230667	2.864891	-0.731934
C	-4.404110	-0.570618	-0.181210
H	-4.880500	0.381219	-0.417352
H	-4.734883	-1.299693	-0.930567
H	-4.799535	-0.912813	0.783793
C	-2.818638	-2.969078	0.248273
H	-3.607115	-2.921219	1.008763
H	-3.308957	-3.252405	-0.691718
H	-2.143939	-3.780885	0.520103
C	0.166311	-2.726330	0.290086
H	1.206925	-2.467348	0.489152
H	-0.151800	-3.429582	1.070949
H	0.156971	-3.280396	-0.658684
C	0.337723	-1.071674	2.903972
H	1.320974	-1.370343	2.531803
H	0.474247	-0.634095	3.899869
H	-0.268309	-1.976206	3.005402
C	-1.371256	2.347185	2.237644
H	-2.457234	2.369687	2.104477
H	-1.149711	2.526468	3.296460
H	-0.939710	3.157234	1.646429
Cl	-0.073406	-0.085015	-2.708296
Cl	0.666579	2.627957	-0.437003
N	2.158581	-0.132450	-0.123187
C	2.858656	0.410465	0.888764
C	2.828398	-0.858650	-1.035738
C	4.231223	0.237860	1.037819
H	2.290598	1.013940	1.588563
C	4.202499	-1.076557	-0.966006
H	2.235322	-1.259571	-1.850652
C	4.918867	-0.520859	0.091432
H	4.744913	0.700260	1.873745
H	4.692218	-1.666514	-1.733144
H	5.991323	-0.669595	0.173751

IM13

B3LYP/BS1 SCF energy: -2486.440071 a.u.

M06-L/BS2 SCF energy in solution: -2486.564667 a.u.

M06-L/BS2 Free energy in solution: -2486.255592 a.u.

Ti	0.342239	-0.079758	-0.595857

C	2.518104	-0.445820	0.250163
C	2.115679	0.953480	0.094883
C	1.182970	1.499085	1.085911
C	1.699116	-1.314954	0.970941
C	0.349469	0.625750	1.772682
C	0.439671	-0.818747	1.503275
C	2.992325	1.898393	-0.698573
H	2.417772	2.751813	-1.057861
H	3.836004	2.274559	-0.101822
H	3.406745	1.410261	-1.581529
C	1.121320	3.002195	1.221374
H	2.118410	3.408126	1.419756
H	0.766372	3.453363	0.288616
H	0.464933	3.333016	2.025115
C	-0.672720	1.078513	2.790695
H	-0.500973	0.569194	3.745185
H	-0.636174	2.150189	2.979134
H	-1.693960	0.835144	2.481543
C	-0.469845	-1.769738	2.252713
H	-0.538072	-2.735268	1.747599
H	-0.117169	-1.963860	3.276575
H	-1.484996	-1.378021	2.332144
C	1.992503	-2.791941	1.106554
H	1.379382	-3.377678	0.412588
H	3.034913	-3.029278	0.901042
H	1.772020	-3.134611	2.121036
C	3.751142	-0.905780	-0.494696
H	4.606594	-0.266951	-0.254516
H	4.024255	-1.933693	-0.264755
H	3.596545	-0.850829	-1.578094
Cl	-0.120530	1.735654	-2.070099
Cl	0.317317	-1.897841	-2.043579
N	-1.911486	-0.058667	-0.288728
C	-2.602097	-1.213152	-0.355471
C	-2.604110	1.083378	-0.111604
C	-3.987727	-1.270889	-0.238963
H	-2.013993	-2.107003	-0.526906
C	-3.987901	1.109951	0.032388
H	-2.020445	1.995420	-0.114536
C	-4.696137	-0.088443	-0.034671
H	-4.492050	-2.228164	-0.311962
H	-4.493266	2.058510	0.177446
H	-5.777648	-0.099405	0.061063

TS13

B3LYP/BS1 SCF energy: -2642.398441 a.u.

M06-L/BS2 SCF energy in solution: -2642.536140 a.u.

M06-L/BS2 Free energy in solution: -2642.152447 a.u.

Ti	0.517957	-0.740820	0.178691
N	1.311627	1.175010	-0.017449
C	2.390707	3.770853	-0.350461
C	2.179031	1.716346	0.891589
C	1.005850	1.947519	-1.104874
C	1.520161	3.221542	-1.293750
C	2.719931	2.984590	0.758472
H	2.425133	1.087443	1.739249
H	0.330551	1.502524	-1.825143
H	1.230257	3.774712	-2.181267
H	3.396424	3.347416	1.525498
H	2.801809	4.766576	-0.475964
Cl	1.646089	-1.616651	2.007773
Cl	-0.129159	-1.228049	-1.998556
C	-1.320554	0.493850	1.435723
C	-1.693932	-0.872269	1.185508
C	-1.639245	-1.926078	2.295496
H	-1.057237	-1.600092	3.153050
H	-1.208705	-2.876941	1.972967
H	-2.658771	-2.134468	2.644219
C	-0.682598	0.902118	2.755610
H	-1.455210	1.223274	3.468345
H	0.002143	1.743790	2.631569
H	-0.116618	0.099057	3.221510
C	-2.697293	1.244297	-0.456961
C	-1.873948	1.547176	0.622699
C	-3.215488	2.312755	-1.400434
H	-3.163914	1.970527	-2.437975
H	-2.638839	3.235333	-1.344409
H	-4.264707	2.569602	-1.202816
C	-1.601778	2.977019	1.045462

H	-1.939262	3.142084	2.075138
H	-2.122182	3.704181	0.424872
H	-0.535769	3.224882	1.020988
C	2.755983	-2.442998	-0.979517
C	3.289075	-1.368453	-1.166080
C	2.211848	-3.790265	-0.817837
H	2.919074	-4.534543	-1.200717
H	2.024400	-4.013663	0.237207
H	1.274041	-3.900212	-1.372251
C	4.026122	-0.142530	-1.462515
H	3.448410	0.524888	-2.110340
H	4.276663	0.409433	-0.551027
H	4.963412	-0.381042	-1.977463
C	-3.085703	-0.113883	-0.700802
C	-4.038322	-0.423196	-1.836441
H	-3.493200	-0.605737	-2.772818
H	-4.635545	-1.313556	-1.633556
H	-4.737413	0.394835	-2.020601
C	-3.002672	-2.573771	-0.199365
H	-2.341914	-3.294123	0.282638
H	-4.021229	-2.786788	0.151476
H	-2.974215	-2.780404	-1.271796
C	-2.595812	-1.142306	0.089092

IM3NN

B3LYP/BS1 SCF energy: -2941.247623 a.u.

M06-L/BS2 SCF energy in solution: -2941.419257 a.u.

M06-L/BS2 Free energy in solution: -2941.018785 a.u.

Ti	0.723961	-0.784567	0.056023
C	3.707940	-0.330383	-0.182752
C	6.332636	-1.286594	-0.426449
C	4.761676	0.307339	0.492932
C	3.979220	-1.448581	-0.984852
C	5.285782	-1.921205	-1.097241
C	6.064567	-0.168562	0.366447
H	4.543924	1.164123	1.123341
H	3.169883	-1.926056	-1.525047
H	5.484722	-2.787304	-1.721496
H	6.870662	0.330305	0.896980
H	7.348187	-1.659272	-0.520115
Cl	0.468434	-0.961376	-2.329065
Cl	1.155729	-2.877013	0.737405
C	1.155860	2.127400	0.668402
N	2.381192	0.143028	-0.019169
C	2.084672	1.500147	-0.147255
C	2.770245	2.247699	-1.278331
H	2.053076	2.471377	-2.076700
H	3.565557	1.644856	-1.714968
H	3.202222	3.195877	-0.944822
C	0.835394	3.587339	0.405140
H	0.884971	3.830921	-0.657240
H	1.533909	4.257877	0.924252
H	-0.170299	3.839542	0.748285
C	0.320897	0.157916	1.864231
C	0.550098	1.503330	1.851141
C	-0.242674	-0.622996	3.021475
H	0.499168	-1.334638	3.402202
H	-1.097038	-1.232532	2.698210
H	-0.580364	-0.002895	3.860355
C	0.234326	2.436360	3.010579
H	0.079896	1.884144	3.938251
H	-0.673733	3.024503	2.825034
H	1.046352	3.150690	3.184319
N	-1.374102	-0.172765	-0.151806
N	-1.983331	-1.230407	0.141117
C	-2.009205	1.088788	-0.454312
C	-3.110654	3.587827	-0.978728
C	-1.650235	1.764567	-1.623917
C	-2.889608	1.662598	0.470408
C	-3.433508	2.917002	0.204190
C	-2.222969	3.009327	-1.887844
H	-0.956644	1.296285	-2.313614
H	-3.132548	1.134908	1.385995
H	-4.110527	3.370172	0.921791
H	-1.967122	3.529431	-2.805837
H	-3.544355	4.561294	-1.186006
C	-3.379660	-1.439065	0.010790
C	-6.067271	-2.168845	-0.185375
C	-3.940222	-2.322172	0.947135

C	-4.157588	-0.959437	-1.057858
C	-5.493674	-1.336324	-1.150964
C	-5.287553	-2.660034	0.864115
H	-3.305261	-2.726129	1.728542
H	-3.713920	-0.329374	-1.818422
H	-6.090273	-0.980212	-1.985114
H	-5.722497	-3.325174	1.603421
H	-7.113152	-2.449278	-0.263532

TS3NN

B3LYP/BS1 SCF energy: -2941.215994 a.u.
M06-L/BS2 SCF energy in solution: -2941.390429 a.u.
M06-L/BS2 Free energy in solution: -2940.990453 a.u.

Ti	0.679514	-0.087791	0.842008
C	3.592727	0.400735	-0.533686
C	5.776135	1.703671	-1.719088
C	4.708710	-0.335542	-0.971792
C	3.571554	1.793078	-0.713106
C	4.659813	2.432336	-1.305352
C	5.794825	0.317283	-1.547411
H	4.710269	-1.414945	-0.862412
H	2.718632	2.358604	-0.359075
H	4.636903	3.510726	-1.432171
H	6.654396	-0.260984	-1.873765
H	6.622714	2.209453	-2.173173
Cl	-0.156603	-1.168870	2.770432
Cl	0.975001	2.058375	1.578655
C	1.579488	-2.409238	0.295412
N	2.481102	-0.265628	0.021827
C	2.556353	-1.487961	0.708861
C	3.598309	-1.747738	1.764154
H	3.112771	-1.968221	2.721332
H	4.235217	-0.872855	1.905083
H	4.238797	-2.602831	1.513893
C	1.412048	-3.733865	0.985128
H	2.356460	-4.288841	1.015275
H	0.668137	-4.355472	0.484827
H	1.077121	-3.579956	2.016351
C	0.919146	-0.637664	-1.147400
C	0.821592	-2.001937	-0.847541
C	0.805111	0.039808	-2.468032
H	1.546805	0.845554	-2.529949
H	-0.179237	0.510391	-2.586624
H	0.974816	-0.636307	-3.315292
C	-0.009460	-2.971162	-1.655009
H	0.551101	-3.879875	-1.903187
H	-0.337877	-2.514230	-2.591977
H	-0.910798	-3.268827	-1.108588
N	-1.420328	0.255164	0.260197
N	-2.009617	-0.835057	-0.006665
C	-2.117723	1.521129	0.309736
C	-3.391602	3.988912	0.516553
C	-1.676864	2.594205	-0.468167
C	-3.172708	1.682133	1.214433
C	-3.802757	2.920388	1.316049
C	-2.329556	3.822343	-0.374173
H	-0.835776	2.464119	-1.139560
H	-3.474520	0.849871	1.840640
H	-4.613704	3.050384	2.026033
H	-1.996430	4.653716	-0.987788
H	-3.887764	4.951139	0.597247
C	-3.319169	-0.930361	-0.542277
C	-5.839342	-1.491588	-1.625629
C	-4.025581	-2.077846	-0.142746
C	-3.870540	-0.081621	-1.520458
C	-5.118612	-0.375950	-2.061371
C	-5.290199	-2.339453	-0.661128
H	-3.565749	-2.736790	0.586490
H	-3.323060	0.786362	-1.865532
H	-5.533409	0.273006	-2.826902
H	-5.838735	-3.214749	-0.327051
H	-6.817427	-1.704583	-2.046057

IM4NN

B3LYP/BS1 SCF energy: -2941.313500 a.u.
M06-L/BS2 SCF energy in solution: -2941.491188 a.u.
M06-L/BS2 Free energy in solution: -2941.087047 a.u.

Ti	0.007164	0.096042	-0.375129
C	-3.763500	-0.029171	0.053492
C	-6.237719	0.178872	-1.198164
C	-4.907552	-0.508124	0.698866
C	-3.844447	0.556712	-1.210599
C	-5.090544	0.659219	-1.830864
C	-6.145958	-0.405289	0.066764
H	-4.822952	-0.951444	1.686194
H	-2.948152	0.920615	-1.699205
H	-5.158212	1.112009	-2.814842
H	-7.036201	-0.777482	0.564036
H	-7.202661	0.259191	-1.689230
Cl	-0.746778	-1.261102	-2.112690
Cl	-0.398460	2.151371	-1.308018
C	-0.709665	-1.032452	1.772665
N	-2.495054	-0.134837	0.736430
C	-1.788951	-1.322021	0.931753
C	-2.289035	-2.638340	0.433040
H	-1.461681	-3.342923	0.340383
H	-2.759659	-2.543976	-0.545506
H	-3.018368	-3.059384	1.135912
C	0.265744	-2.015199	2.353821
H	1.274623	-1.597022	2.384476
H	0.309008	-2.937448	1.770820
H	-0.020460	-2.279379	3.379108
C	-1.911088	0.893967	1.481784
C	-0.801907	0.363220	2.132729
C	-2.503567	2.262801	1.518774
H	-2.440711	2.750302	0.541318
H	-1.963890	2.881158	2.237050
H	-3.556113	2.225409	1.817814
C	0.089165	1.047756	3.122284
H	-0.102408	2.122179	3.164584
H	1.136514	0.899248	2.846574
H	-0.067515	0.638422	4.127188
N	1.735427	-0.546876	-0.423602
N	1.774688	0.573419	0.439315
C	2.614641	-1.623029	-0.302271
C	4.365786	-3.807221	-0.100816
C	2.430223	-2.756775	-1.118196
C	3.691788	-1.586760	0.602674
C	4.552618	-2.679051	0.699050
C	3.302132	-3.834746	-1.009346
H	1.609419	-2.767743	-1.826512
H	3.841670	-0.702916	1.211256
H	5.380516	-2.641504	1.401717
H	3.152616	-4.704185	-1.643459
H	5.043679	-4.651870	-0.024931
C	2.633237	1.626836	0.034983
C	4.367286	3.756162	-0.583843
C	2.636528	2.798497	0.811966
C	3.513891	1.535037	-1.055713
C	4.369506	2.594012	-1.356054
C	3.491380	3.850916	0.501008
H	1.961679	2.871465	1.658376
H	3.519218	0.639611	-1.666077
H	5.043212	2.506612	-2.204146
H	3.475661	4.750112	1.110945
H	5.037263	4.576515	-0.822684

TS3CC

B3LYP/BS1 SCF energy: -2524.431788 a.u.

M06-L/BS2 SCF energy in solution: -2524.559694 a.u.

M06-L/BS2 Free energy in solution: -2524.256761 a.u.

Ti	0.765003	0.278551	-0.514260
C	-2.432472	-0.048520	-0.021109
C	-5.150663	0.571705	0.265650
C	-3.355046	-1.048538	0.334781
C	-2.878725	1.268048	-0.215501
C	-4.231772	1.568362	-0.068299
C	-4.705748	-0.737467	0.464598
H	-3.002984	-2.059356	0.512868
H	-2.165901	2.030615	-0.504620
H	-4.569695	2.588096	-0.227335
H	-5.411325	-1.518556	0.732237
H	-6.203993	0.812255	0.371954
Cl	2.269668	-0.718681	-2.062417
Cl	0.169781	2.166628	-1.689584

C	0.508708	-2.064258	0.331195
N	-1.058827	-0.355586	-0.118497
C	-0.551295	-1.614459	-0.483601
C	-1.109983	-2.379633	-1.654552
H	-0.335326	-2.511536	-2.418323
H	-1.941386	-1.840013	-2.111163
H	-1.464891	-3.376010	-1.365240
C	1.227238	-3.344341	0.002632
H	0.536181	-4.194779	0.008633
H	2.027703	-3.559464	0.711356
H	1.674871	-3.279377	-0.994050
C	0.178790	0.033930	1.417732
C	0.803296	-1.232174	1.448753
C	-0.326323	0.824475	2.573689
H	-1.140873	1.483011	2.253372
H	0.472342	1.471648	2.958080
H	-0.690418	0.201487	3.399647
C	1.593691	-1.689469	2.655768
H	0.951800	-2.219794	3.371967
H	2.044657	-0.844132	3.182545
H	2.399982	-2.372803	2.379159
C	3.174178	1.093280	0.607327
C	2.443085	2.066037	0.697302
C	4.221825	0.070962	0.578193
H	4.002844	-0.748829	1.268293
H	4.326276	-0.345135	-0.426213
H	5.173017	0.524390	0.879034
C	1.827116	3.381249	0.907942
H	0.832174	3.304410	1.353154
H	2.460082	3.973522	1.578157
H	1.720558	3.911109	-0.041304

IM4CC

B3LYP/BS1 SCF energy: -2524.512845 a.u.

M06-L/BS2 SCF energy in solution: -2524.637809 a.u.

M06-L/BS2 Free energy in solution: -2524.330655 a.u.

Ti	1.125609	0.564335	0.000045
C	-2.502746	-0.248493	-0.000027
C	-5.096706	0.747283	0.000435
C	-3.582904	-1.135871	0.000727
C	-2.703801	1.132241	-0.000568
C	-4.010603	1.623394	-0.000334
C	-4.883075	-0.632734	0.000965
H	-3.400690	-2.206275	0.001087
H	-1.854076	1.805038	-0.001125
H	-4.173773	2.696301	-0.000735
H	-5.725355	-1.317571	0.001547
H	-6.109573	1.138358	0.000623
Cl	0.507403	1.964058	-1.774772
Cl	0.506135	1.964142	1.774283
C	0.706021	-1.820864	-0.723554
N	-1.166480	-0.800008	-0.000292
C	-0.468260	-1.193191	-1.142715
C	-1.036331	-1.007379	-2.510796
H	-0.275473	-1.223896	-3.261554
H	-1.375553	0.018736	-2.664301
H	-1.882356	-1.685344	-2.672930
C	1.709946	-2.510612	-1.602019
H	1.478492	-3.578773	-1.694837
H	2.718098	-2.421320	-1.192091
H	1.724994	-2.082209	-2.606480
C	-0.467985	-1.193617	1.141876
C	0.706143	-1.821211	0.722193
C	-1.035922	-1.008573	2.510125
H	-1.375969	0.017225	2.663921
H	-0.274721	-1.224596	3.260683
H	-1.881339	-1.687284	2.672305
C	1.710069	-2.511597	1.600144
H	1.478739	-3.579872	1.691920
H	1.724907	-2.084141	2.605012
H	2.718269	-2.421757	1.190448
C	3.003722	0.464677	-0.657386
C	3.003088	0.464504	0.659252
C	3.942420	0.555674	-1.811518
H	3.847634	-0.318200	-2.467554
H	3.689932	1.428504	-2.423790
H	4.993011	0.635246	-1.502764
C	3.940646	0.555224	1.814326
H	3.845286	-0.318853	2.470004

H	4.991533	0.634966	1.506628
H	3.687486	1.427864	2.426600

TS3PhCF$_3$
B3LYP/BS1 SCF energy: -2937.744220 a.u.
M06-L/BS2 SCF energy in solution: -2937.931324 a.u.
M06-L/BS2 Free energy in solution: -2937.616418 a.u.

Ti	0.545579	0.380435	-0.732907
C	3.427217	-0.891887	0.000383
C	5.472164	-2.752847	0.419194
C	4.674427	-0.469808	0.489645
C	3.202890	-2.250859	-0.265487
C	4.226248	-3.172451	-0.051603
C	5.691920	-1.399492	0.687570
H	4.832865	0.579550	0.715057
H	2.238191	-2.561099	-0.650811
H	4.050002	-4.222594	-0.263869
H	6.656391	-1.067150	1.059861
H	6.267103	-3.475137	0.577472
Cl	-0.544464	1.958439	-2.087513
Cl	-0.105505	-1.642345	-1.602505
C	1.778424	2.279680	0.231300
N	2.379263	0.038628	-0.181900
C	2.560107	1.400090	-0.548311
C	3.562328	1.795265	-1.603001
H	3.082868	2.403097	-2.376690
H	3.976307	0.909716	-2.089383
H	4.398462	2.375219	-1.192870
C	1.716949	3.759166	-0.035426
H	2.444993	4.061384	-0.789386
H	1.929601	4.323055	0.878647
H	0.725147	4.050170	-0.395517
C	1.002249	0.240004	1.224421
C	1.012273	1.657534	1.251397
C	0.967442	-0.722829	2.355755
H	1.433928	-1.665697	2.051035
H	-0.073274	-0.963930	2.598863
H	1.471226	-0.353343	3.256555
C	0.247829	2.423704	2.308450
H	-0.127830	3.369655	1.910682
H	0.883581	2.653921	3.173412
H	-0.614218	1.856259	2.667439
C	-4.088995	-0.505206	0.392896
C	-4.025514	-1.090720	-0.877101
C	-5.206846	-1.351649	-1.566802
C	-6.440834	-1.032961	-0.993952
C	-6.496372	-0.450390	0.272393
C	-5.318694	-0.184226	0.970855
H	-3.063465	-1.332842	-1.318295
H	-5.163193	-1.801188	-2.553708
H	-7.358835	-1.237562	-1.536865
H	-7.454385	-0.200543	0.717631
H	-5.352176	0.270719	1.954154
C	-2.810382	-0.248787	1.132039
F	-2.987689	0.418738	2.292171
F	-2.150048	-1.391110	1.430044
F	-1.942305	0.508683	0.385170

IM4PhCF$_3$
B3LYP/BS1 SCF energy: -2937.768748 a.u.
M06-L/BS2 SCF energy in solution: -2937.955274 a.u.
M06-L/BS2 Free energy in solution: -2937.637467 a.u.

Ti	0.459608	-0.039012	-0.719381
C	3.602808	-0.162732	0.341714
C	6.235044	-1.027637	0.619439
C	4.506647	0.593708	1.096577
C	4.005251	-1.347145	-0.274351
C	5.326826	-1.775546	-0.129913
C	5.823372	0.158284	1.232847
H	4.172681	1.512662	1.568584
H	3.287528	-1.915901	-0.854283
H	5.643333	-2.697098	-0.608709
H	6.526457	0.743856	1.817507
H	7.261404	-1.365639	0.726077
Cl	-0.953134	1.062252	-2.274211
Cl	0.812953	-2.107078	-1.672609

C	0.889197	2.210849	0.291463
N	2.243087	0.310666	0.218676
C	1.881803	1.530318	-0.507274
C	2.798800	2.116950	-1.535587
H	2.224153	2.761963	-2.204743
H	3.262011	1.337041	-2.145933
H	3.606774	2.714825	-1.091047
C	0.324609	3.546125	-0.086949
H	1.112689	4.307705	-0.102332
H	-0.445592	3.874394	0.613775
H	-0.124212	3.502212	-1.084808
C	1.229202	0.129100	1.266780
C	0.498570	1.379422	1.337033
C	1.448166	-0.863527	2.367790
H	1.795054	-1.823168	1.976779
H	0.505156	-1.041364	2.891241
H	2.183058	-0.514734	3.107293
C	-0.575206	1.628805	2.354119
H	-0.143366	1.725987	3.357421
H	-1.288747	0.799869	2.385946
H	-1.135213	2.540473	2.138066
C	-3.977466	-0.653151	0.257369
C	-6.304168	0.837291	0.565600
C	-4.257495	0.382095	-0.642793
C	-4.851280	-0.945647	1.307266
C	-6.018054	-0.196427	1.458131
C	-5.424435	1.125380	-0.481248
H	-3.560909	0.603653	-1.446113
H	-4.623378	-1.754836	1.991781
H	-6.702340	-0.422838	2.269887
H	-5.647534	1.929578	-1.175323
H	-7.213674	1.418828	0.684399
C	-2.717413	-1.444726	0.078612
F	-2.624029	-2.494052	0.910162
F	-1.595274	-0.646948	0.379417
F	-2.535574	-1.872621	-1.169980

TS4a

B3LYP/BS1 SCF energy: -2616.768071a.u.

M06-L/BS2 SCF energy in solution: -2616.892823 a.u.

M06-L/BS2 Free energy in solution: -2616.587169 a.u.

Ti	-0.104924	0.706118	-0.768560
N	-3.073904	-0.834691	0.416048
C	-5.635802	-1.954829	0.213452
C	-4.131079	-0.092574	0.053972
C	-3.290649	-2.133087	0.669235
C	-4.543754	-2.739084	0.582947
C	-5.424859	-0.604297	-0.057860
H	-3.921025	0.950168	-0.168880
H	-2.417604	-2.717931	0.951340
H	-4.655061	-3.797066	0.798248
H	-6.241627	0.045117	-0.356134
H	-6.628414	-2.388786	0.134057
C	2.701166	-0.824780	0.156873
C	4.832274	-2.621499	0.076295
C	3.561846	-0.937058	1.255838
C	2.903681	-1.604336	-0.982406
C	3.974540	-2.500152	-1.016816
C	4.623334	-1.838946	1.214380
H	3.395659	-0.316423	2.130785
H	2.223426	-1.512656	-1.820554
H	4.131498	-3.107117	-1.903202
H	5.288785	-1.926163	2.067918
H	5.660757	-3.322581	0.043457
Cl	-1.710230	2.312516	-1.219733
Cl	-0.147618	-0.747354	-2.532837
C	0.960911	2.264025	0.805526
N	1.622230	0.130090	0.232384
C	1.774923	1.542336	-0.140854
C	3.016290	2.022919	-0.828431
H	2.849753	3.032835	-1.210844
H	3.279800	1.386224	-1.675933
H	3.880070	2.054705	-0.149087
C	0.863304	3.759897	0.799133
H	1.765992	4.206587	1.232789
H	0.002904	4.114097	1.368736
H	0.758777	4.137486	-0.222492
C	0.507623	0.014833	1.172317
C	0.209900	1.367014	1.569091

C	0.265533	-1.243320	1.946792
H	0.402830	-2.126544	1.318099
H	-0.765281	-1.245846	2.307553
H	0.933703	-1.341670	2.814112
C	-0.893097	1.686742	2.533802
H	-0.594583	1.440872	3.559868
H	-1.786469	1.099154	2.298136
H	-1.166948	2.742556	2.505313

IM5a

B3LYP/BS1 SCF energy: -2368.471332 a.u.

M06-L/BS2 SCF energy in solution: -2368.572322 a.u.

M06-L/BS2 Free energy in solution: -2368.345534 a.u.

Ti	1.190556	0.824958	0.000371
C	-1.984360	-0.180325	-0.000013
C	-4.764743	0.005275	-0.000139
C	-2.760967	-1.345564	0.000053
C	-2.590091	1.075802	-0.000141
C	-3.984280	1.161083	-0.000217
C	-4.150972	-1.249695	-0.000007
H	-2.270961	-2.314341	0.000133
H	-1.974458	1.967172	-0.000163
H	-4.456979	2.138370	-0.000325
H	-4.752984	-2.153223	0.000043
H	-5.847933	0.079887	-0.000190
Cl	3.473151	0.972440	0.000062
Cl	0.392011	2.956724	0.000689
C	1.285078	-1.526546	-0.700151
N	-0.547391	-0.329710	-0.000015
C	0.217154	-0.697701	-1.190892
C	-0.426526	-0.732546	-2.543352
H	0.348209	-0.773104	-3.312862
H	-1.027850	0.161436	-2.722958
H	-1.077789	-1.608759	-2.670724
C	2.314087	-2.126346	-1.611112
H	1.884469	-2.952785	-2.189028
H	3.173975	-2.508705	-1.059786
H	2.684668	-1.380087	-2.321766
C	0.217153	-0.698348	1.190721
C	1.285086	-1.526918	0.699458
C	-0.426731	-0.734535	2.543053
H	-1.029017	0.158736	2.722983
H	0.347887	-0.774645	3.312707
H	-1.077103	-1.611489	2.669873
C	2.314153	-2.127148	1.610077
H	1.884485	-2.953595	2.187944
H	2.685063	-1.381097	2.320784
H	3.173824	-2.509605	1.058481

TS5a

B3LYP/BS1 SCF energy: -2941.242185 a.u.

M06-L/BS2 SCF energy in solution: -2941.420018 a.u.

M06-L/BS2 Free energy in solution: -2941.016476 a.u.

Ti	0.142575	-0.783451	0.887135
C	-2.755935	-0.795852	-0.731038
C	-5.339384	0.067291	-1.324386
C	-3.590243	-1.588148	-1.527815
C	-3.205697	0.428392	-0.233712
C	-4.498300	0.856574	-0.539717
C	-4.884042	-1.157649	-1.816255
H	-3.220784	-2.530045	-1.920541
H	-2.563282	1.028936	0.397764
H	-4.844722	1.809710	-0.153046
H	-5.531525	-1.775709	-2.430723
H	-6.346167	0.403984	-1.552236
Cl	1.956348	-1.630851	2.044676
Cl	-1.305363	-0.246496	2.626446
C	0.675987	-1.898924	-1.170084
N	-1.412213	-1.256014	-0.481277
C	-0.260445	-0.812087	-1.260784
C	-0.409762	0.167470	-2.381675
H	0.568910	0.578730	-2.640424
H	-1.055827	1.002904	-2.107472
H	-0.828362	-0.305343	-3.281018
C	2.025396	-1.915820	-1.826297
H	2.008931	-2.534782	-2.731745

H	2.787987	-2.321459	-1.156068
H	2.347216	-0.913409	-2.114218
C	-1.099496	-2.510550	0.166317
C	0.156100	-2.914355	-0.342338
C	-2.126807	-3.289518	0.921537
H	-2.748780	-2.629848	1.529784
H	-1.627684	-3.988151	1.596437
H	-2.781724	-3.869023	0.257180
C	0.852375	-4.184605	0.041951
H	0.594718	-4.982146	-0.665639
H	0.560319	-4.511611	1.041969
H	1.936676	-4.062501	0.038067
N	1.978754	2.038043	-0.515914
N	1.139683	1.734384	0.397356
C	3.210186	1.369624	-0.525969
C	5.745299	0.203016	-0.742203
C	3.922618	1.432300	-1.739731
C	3.796603	0.748831	0.591884
C	5.057420	0.169965	0.474072
C	5.174886	0.838147	-1.850560
H	3.468912	1.954735	-2.576324
H	3.272245	0.745790	1.537809
H	5.506509	-0.306319	1.340189
H	5.713658	0.880726	-2.792502
H	6.729541	-0.249070	-0.821838
C	0.021519	2.639230	0.392498
C	-2.121313	4.434246	0.545806
C	-0.634109	2.849498	1.611269
C	-0.388844	3.347671	-0.750201
C	-1.461208	4.230424	-0.671021
C	-1.698265	3.749024	1.685424
H	-0.302823	2.313098	2.491307
H	0.151483	3.204278	-1.677512
H	-1.781013	4.767651	-1.559387
H	-2.194703	3.910343	2.637286
H	-2.953896	5.129295	0.603866

TS4b

B3LYP/BS1 SCF energy: -3189.545657 a.u.

M06-L/BS2 SCF energy in solution: -3189.748341 a.u.

M06-L/BS2 Free energy in solution: -3189.260088 a.u.

Ti	1.293574	-0.147226	0.210430
N	3.308642	-1.145925	-0.369362
C	5.793111	-2.225616	-1.080101
C	4.094637	-1.743747	0.546820
C	3.752635	-1.091922	-1.640256
C	4.983442	-1.612690	-2.033629
C	5.335518	-2.292694	0.234388
H	3.688074	-1.794711	1.549834
H	3.075679	-0.639231	-2.353990
H	5.286956	-1.541278	-3.072609
H	5.922487	-2.765542	1.014468
H	6.755626	-2.646382	-1.355928
C	-0.212745	2.781628	-0.474427
C	-2.029403	4.590256	-1.575404
C	-0.381681	4.042398	0.107740
C	-0.946103	2.420792	-1.606428
C	-1.846929	3.334038	-2.157410
C	-1.296461	4.941188	-0.439995
H	0.204575	4.313362	0.979725
H	-0.801324	1.436082	-2.037640
H	-2.410185	3.058094	-3.044273
H	-1.429206	5.917911	0.015876
H	-2.736025	5.293723	-2.005819
Cl	1.208908	-1.762645	2.009931
Cl	0.547135	-0.835805	-1.974506
C	2.213915	1.470469	1.866535
N	0.755128	1.870272	0.085448
C	0.823029	1.515032	1.499241
C	-0.336996	1.695390	2.427759
H	-0.252904	0.977103	3.247798
H	-1.282397	1.510374	1.915990
H	-0.369626	2.704107	2.864016
C	2.682172	1.225281	3.268602
H	2.440488	2.079047	3.913689
H	3.762716	1.070023	3.314327
H	2.195612	0.339289	3.687763
C	2.125193	1.779916	-0.419304
C	2.987935	1.649382	0.722353

C	2.466829	2.320086	-1.773230
H	1.822353	1.901030	-2.549820
H	3.499093	2.059498	-2.021508
H	2.382914	3.415234	-1.815500
C	4.484567	1.683099	0.639972
H	4.839870	2.720934	0.612565
H	4.852702	1.191668	-0.264696
H	4.951200	1.195001	1.498455
N	-1.593346	-0.849830	0.316029
N	-2.398866	0.087654	0.499936
C	-2.025221	-2.153646	-0.128314
C	-2.690679	-4.704360	-1.034377
C	-1.579290	-3.276181	0.575737
C	-2.769815	-2.301686	-1.305553
C	-3.093657	-3.578150	-1.756372
C	-1.936426	-4.548522	0.129576
H	-0.957597	-3.137627	1.452327
H	-3.070382	-1.425421	-1.868322
H	-3.659129	-3.693336	-2.676332
H	-1.606683	-5.420439	0.686704
H	-2.950732	-5.698022	-1.387144
C	-3.827456	-0.047990	0.483034
C	-6.617699	0.018420	0.618335
C	-4.535873	0.994481	-0.129537
C	-4.516129	-1.035344	1.203731
C	-5.906586	-0.989850	1.273633
C	-5.928058	1.007913	-0.085892
H	-3.980583	1.777264	-0.636525
H	-3.966989	-1.819935	1.710745
H	-6.437165	-1.749245	1.840393
H	-6.474232	1.803890	-0.583471
H	-7.702101	0.040725	0.669504

IM5b

B3LYP/BS1 SCF energy: -3189.551630 a.u.

M06-L/BS2 SCF energy in solution: -3189.770653 a.u.

M06-L/BS2 Free energy in solution: -3189.281855 a.u.

Ti	0.945657	-0.052927	0.177370
N	3.250872	-0.855659	-0.120178
C	5.949905	-1.601750	-0.387945
C	3.993369	-1.210118	0.950302
C	3.860599	-0.873820	-1.323695
C	5.194129	-1.237258	-1.498478
C	5.331503	-1.584986	0.860085
H	3.471242	-1.212110	1.899636
H	3.236366	-0.602488	-2.166517
H	5.618719	-1.235634	-2.496844
H	5.867158	-1.864064	1.761312
H	6.990643	-1.894572	-0.491907
C	-1.065882	2.675537	-0.547908
C	-3.344892	3.800818	-1.684726
C	-1.625396	3.825435	0.015179
C	-1.635987	2.081955	-1.675291
C	-2.771434	2.657766	-2.244962
C	-2.771324	4.381859	-0.552469
H	-1.162978	4.285994	0.881475
H	-1.186860	1.187413	-2.091597
H	-3.214594	2.201193	-3.124624
H	-3.206655	5.274985	-0.114859
H	-4.233529	4.238634	-2.129296
Cl	1.160352	-1.277339	2.335403
Cl	0.896900	-0.433318	-2.256249
C	1.744092	1.852901	1.629065
N	0.147797	2.140650	0.023163
C	0.350340	1.853740	1.405714
C	-0.734870	1.855734	2.430746
H	-0.437685	1.207231	3.257499
H	-1.662197	1.464944	2.011383
H	-0.906755	2.863731	2.829284
C	2.400348	1.736181	2.971078
H	2.283545	2.670753	3.533810
H	3.470507	1.539038	2.874766
H	1.962935	0.922154	3.552856
C	1.409605	2.301136	-0.589027
C	2.390570	2.126568	0.391727
C	1.552687	2.816278	-1.982894
H	1.116657	2.136829	-2.717424
H	2.610686	2.939153	-2.220682
H	1.067285	3.794753	-2.075348

C	3.866488	2.296764	0.180797
H	4.161826	3.335057	0.374060
H	4.161619	2.050765	-0.841192
H	4.443442	1.659076	0.853268
N	-0.950604	-0.811323	0.199004
N	-2.019131	-0.129755	0.504184
C	-1.075975	-2.121568	-0.415750
C	-1.239709	-4.573396	-1.758208
C	-0.317355	-3.211637	0.023180
C	-1.911094	-2.260240	-1.536755
C	-1.991072	-3.478339	-2.198397
C	-0.408649	-4.433835	-0.650007
H	0.306797	-3.111009	0.901995
H	-2.480524	-1.406837	-1.884856
H	-2.635652	-3.573713	-3.067361
H	0.172948	-5.279281	-0.293768
H	-1.304948	-5.524352	-2.278715
C	-3.272718	-0.719990	0.719457
C	-5.884769	-1.601867	1.325237
C	-4.395196	0.087209	0.437886
C	-3.485523	-1.970421	1.342064
C	-4.777057	-2.394475	1.639024
C	-5.681907	-0.356177	0.725150
H	-4.229389	1.059319	-0.015329
H	-2.638394	-2.587725	1.614509
H	-4.918830	-3.354113	2.128982
H	-6.531966	0.277401	0.486018
H	-6.888665	-1.944381	1.557421

TS5b

B3LYP/BS1 SCF energy: -3189.550703 a.u.

M06-L/BS2 SCF energy in solution: -3189.765451 a.u.

M06-L/BS2 Free energy in solution: -3189.275980 a.u.

Ti	-0.876063	0.122896	0.265003
N	-3.389061	0.701810	-0.490196
C	-6.000238	1.236042	-1.385969
C	-4.192824	1.560690	0.161903
C	-3.887343	0.117565	-1.596594
C	-5.177262	0.343884	-2.070342
C	-5.491206	1.859337	-0.250548
H	-3.766977	2.016798	1.047857
H	-3.207335	-0.528791	-2.135768
H	-5.515930	-0.164919	-2.966848
H	-6.081722	2.570436	0.317638
H	-7.008206	1.443894	-1.733091
C	0.983267	-2.855931	-0.381144
C	2.838566	-4.411324	-1.755663
C	1.379120	-4.090773	0.142280
C	1.501264	-2.393213	-1.589755
C	2.425911	-3.183402	-2.274935
C	2.313860	-4.864846	-0.544501
H	0.949088	-4.446317	1.073551
H	1.180436	-1.436041	-1.979376
H	2.828152	-2.829015	-3.219103
H	2.621766	-5.822767	-0.136744
H	3.563388	-5.014738	-2.293804
Cl	-1.681775	1.491185	2.080073
Cl	-0.789836	0.217828	-2.159457
C	-1.177859	-1.608289	2.229726
N	-0.026900	-2.108455	0.343334
C	0.105100	-1.750490	1.708379
C	1.422727	-1.684204	2.395409
H	1.313403	-1.176044	3.354857
H	2.134614	-1.133392	1.772177
H	1.829782	-2.683892	2.582222
C	-1.526378	-1.319679	3.657269
H	-0.705933	-0.827305	4.181010
H	-1.752340	-2.257131	4.181361
H	-2.395860	-0.665015	3.727104
C	-1.406590	-2.216444	0.026415
C	-2.119097	-1.886377	1.187560
C	-1.866347	-2.893291	-1.224176
H	-1.441216	-2.429559	-2.116526
H	-2.954081	-2.840600	-1.292729
H	-1.580145	-3.951930	-1.211528
C	-3.606322	-1.924314	1.376614
H	-3.884309	-2.793466	1.985118
H	-4.138345	-1.993905	0.427166
H	-3.960513	-1.029355	1.894301

N	0.957135	0.883329	0.217252
N	2.067746	0.180054	0.097551
C	0.984266	2.290003	-0.176059
C	1.074258	4.954416	-1.029021
C	0.586843	3.315288	0.687412
C	1.431794	2.603049	-1.469680
C	1.472682	3.928053	-1.889849
C	0.636723	4.642936	0.257320
H	0.249125	3.076131	1.686451
H	1.732257	1.802069	-2.132525
H	1.814297	4.159605	-2.894431
H	0.334389	5.433339	0.938208
H	1.106731	5.988313	-1.360330
C	3.336750	0.764410	0.203558
C	6.022459	1.624829	0.427253
C	4.376532	0.102710	-0.486487
C	3.683207	1.852568	1.036475
C	5.007905	2.269099	1.138953
C	5.693955	0.533962	-0.383108
H	4.117713	-0.749941	-1.105978
H	2.918409	2.353692	1.616158
H	5.249863	3.104811	1.790577
H	6.471539	0.013677	-0.936352
H	7.051937	1.959699	0.511091

1cat^{T}

B3LYP/BS1 SCF energy: -2801.320795 a.u.
M06-L/BS2 SCF energy in solution: -2801.465724 a.u.
M06-L/BS2 Free energy in solution: -2801.162960 a.u.

Ti	0.032942	-0.000003	-0.000014
N	0.328808	-2.279005	-0.037254
C	0.483427	-5.070456	-0.109278
C	0.884233	-2.915399	-1.086058
C	-0.153253	-3.026114	0.975744
C	-0.099056	-4.416950	0.975439
C	0.986100	-4.302187	-1.157290
H	1.229407	-2.278114	-1.892328
H	-0.575508	-2.471945	1.806380
H	-0.505506	-4.968663	1.816378
H	1.444483	-4.761529	-2.026515
H	0.542806	-6.154575	-0.137485
N	0.328917	2.278987	0.037236
C	0.483672	5.070431	0.109280
C	-0.153000	3.026122	-0.975811
C	0.884265	2.915352	1.086100
C	0.986193	4.302135	1.157344
C	-0.098730	4.416955	-0.975500
H	-0.575199	2.471976	-1.806491
H	1.229323	2.278046	1.892404
H	1.444508	4.761453	2.026619
H	-0.505063	4.968690	-1.816481
H	0.543103	6.154547	0.137494
N	2.459907	-0.000045	0.000012
C	5.258012	-0.000069	0.000051
C	3.154044	0.504798	-1.037778
C	3.154006	-0.504901	1.037820
C	4.545848	-0.528467	1.074924
C	4.545887	0.528340	-1.074842
H	2.556551	0.878286	-1.862524
H	2.556483	-0.878378	1.862550
H	5.052230	-0.950790	1.936299
H	5.052301	0.950654	-1.936203
H	6.344163	-0.000079	0.000066
N	-1.838032	0.000049	-0.000032
C	-3.159742	0.000072	0.000006
C	-5.992175	0.000125	0.000013
C	-3.908809	1.225572	0.072358
C	-3.908855	-1.225397	-0.072382
C	-5.291905	-1.215127	-0.073796
C	-5.291860	1.215351	0.073798
H	-3.349501	2.153479	0.130337
H	-3.349580	-2.153323	-0.130388
H	-5.842030	-2.150202	-0.132101
H	-5.841949	2.150447	0.132105
H	-7.078184	0.000145	0.000021
Cl	0.179307	0.051333	-2.456163
Cl	0.179247	-0.051396	2.456138

2cat^{T}

B3LYP/BS1 SCF energy: -2553.010989 a.u.
M06-L/BS2 SCF energy in solution: -2553.119026 a.u.
M06-L/BS2 Free energy in solution: -2552.900053 a.u.

Ti	-0.000029	-1.002079	-0.000003
N	2.273192	-0.905461	-0.000010
C	5.037965	-0.525629	-0.000045
C	2.956898	-0.815704	1.156718
C	2.956895	-0.815896	-1.156756
C	4.334551	-0.625238	-1.199176
C	4.334552	-0.625034	1.199105
H	2.367810	-0.921813	2.060866
H	2.367808	-0.922162	-2.060887
H	4.838702	-0.563251	-2.157431
H	4.838706	-0.562884	2.157348
H	6.114064	-0.379522	-0.000055
N	-2.273245	-0.905350	0.000018
C	-5.038007	-0.525440	-0.000020
C	-2.957002	-0.815968	1.156745
C	-2.956890	-0.815370	-1.156729
C	-4.334537	-0.624653	-1.199152
C	-4.334655	-0.625280	1.199129
H	-2.367961	-0.922403	2.060886
H	-2.367763	-0.921340	-2.060867
H	-4.838641	-0.562320	-2.157409
H	-4.838852	-0.563447	2.157370
H	-6.114102	-0.379305	-0.000036
N	0.000019	0.879481	0.000000
C	0.000059	2.199931	0.000031
C	0.000152	5.032760	0.000091
C	0.000053	2.950888	-1.229176
C	0.000117	2.950835	1.229270
C	0.000158	4.333346	1.218962
C	0.000102	4.333397	-1.218810
H	0.000007	2.390196	-2.158504
H	0.000127	2.390107	2.158576
H	0.000196	4.884335	2.155131
H	0.000100	4.884426	-2.154956
H	0.000188	6.118685	0.000114
Cl	0.000014	-2.215526	2.035110
Cl	-0.000129	-2.215411	-2.035185

TS1^{T}

B3LYP/BS1 SCF energy: -2708.966910 a.u.
M06-L/BS2 SCF energy in solution: -2709.104078 a.u.
M06-L/BS2 Free energy in solution: -2708.804481 a.u.

Ti	0.393947	0.274664	0.105170
N	-0.863423	2.155842	0.020255
C	-2.565315	4.368157	-0.138943
C	-1.914271	2.164187	-0.825782
C	-0.656971	3.245293	0.787285
C	-1.475184	4.369530	0.728133
C	-2.790057	3.239404	-0.925090
H	-2.035135	1.282326	-1.441734
H	0.179416	3.184018	1.473630
H	-1.257331	5.221706	1.362649
H	-3.624764	3.186574	-1.615356
H	-3.226158	5.227680	-0.201200
N	2.753205	0.007461	-0.171794
C	5.535740	-0.260376	-0.351778
C	3.430548	0.478094	-1.237039
C	3.461326	-0.591458	0.807053
C	4.841024	-0.756466	0.750486
C	4.814409	0.373675	-1.359628
H	2.826429	0.933297	-2.013194
H	2.893162	-0.925878	1.665261
H	5.354395	-1.254730	1.565683
H	5.304646	0.780693	-2.237397
H	6.614620	-0.363923	-0.420718
C	-2.292589	-1.374858	0.145089
C	-4.869757	-2.399402	-0.369366
C	-3.233736	-1.538595	1.192882
C	-2.683078	-1.734810	-1.170139
C	-3.954133	-2.240921	-1.416319
C	-4.504562	-2.040890	0.932296
H	-2.946782	-1.239643	2.195990
H	-1.966139	-1.594896	-1.973145

H	-4.238253	-2.512162	-2.429503
H	-5.216909	-2.151373	1.745265
H	-5.861942	-2.793911	-0.567816
Cl	0.402005	0.405082	-2.339459
Cl	1.123544	1.003603	2.325387
C	0.881373	-2.600929	-0.090364
N	-1.047863	-0.856376	0.395073
C	0.262994	-2.220440	0.932298
C	0.018078	-2.452083	2.377224
H	0.659571	-3.260420	2.741460
H	-1.025975	-2.741854	2.533114
H	0.211926	-1.542879	2.951478
C	1.400717	-2.940092	-1.407770
H	2.496313	-2.915821	-1.419632
H	1.044409	-2.230382	-2.166257
H	1.080573	-3.949279	-1.691011

IM1^{T}

B3LYP/BS1 SCF energy: -2709.034285 a.u.

M06-L/BS2 SCF energy in solution: -2709.164288 a.u.

M06-L/BS2 Free energy in solution: -2708.860626 a.u.

Ti	-0.452447	-0.493238	0.010617
N	-0.215871	1.900840	-0.022243
C	0.140469	4.679311	-0.034980
C	-0.723586	2.673204	0.958251
C	0.470707	2.510853	-1.008225
C	0.674798	3.887398	-1.049227
C	-0.574943	4.056939	0.986484
H	-1.246747	2.146212	1.748450
H	0.849352	1.862109	-1.790076
H	1.241998	4.320412	-1.866156
H	-1.010727	4.626563	1.800381
H	0.279130	5.756421	-0.039517
N	-2.754665	-0.310857	-0.058963
C	-5.550790	-0.216908	-0.158297
C	-3.505477	-0.924702	0.877675
C	-3.394121	0.349104	-1.044998
C	-4.781196	0.423593	-1.127348
C	-4.896458	-0.905139	0.861923
H	-2.956581	-1.428326	1.664962
H	-2.759104	0.808252	-1.793584
H	-5.239949	0.971521	-1.943296
H	-5.447696	-1.421221	1.640521
H	-6.635387	-0.181831	-0.197703
C	2.889062	-0.594413	0.088182
C	5.391826	0.691042	0.118352
C	3.943228	-1.059898	-0.722918
C	3.104509	0.540191	0.895969
C	4.347464	1.164200	0.918107
C	5.178752	-0.415933	-0.705492
H	3.777960	-1.896893	-1.391339
H	2.288226	0.892989	1.517539
H	4.502083	2.025919	1.561167
H	5.977716	-0.779206	-1.345613
H	6.358803	1.184779	0.131225
Cl	-0.597590	-0.338733	2.379489
Cl	-0.433611	-0.381226	-2.363512
C	-0.108510	-2.610562	-0.005633
N	1.610451	-1.155457	0.086489
C	1.292899	-2.470365	0.058004
C	2.308882	-3.586669	0.145875
H	1.884731	-4.434954	0.688663
H	3.220032	-3.264884	0.655537
H	2.591123	-3.948382	-0.850125
C	-0.806723	-3.922896	-0.100839
H	-1.233166	-4.032096	-1.109280
H	-1.661862	-3.962813	0.585704
H	-0.179844	-4.805829	0.085185

IM2^{T}

B3LYP/BS1 SCF energy: -2460.716452 a.u.

M06-L/BS2 SCF energy in solution: -2460.815237 a.u.

M06-L/BS2 Free energy in solution: -2460.595162 a.u.

Ti	-0.477593	-1.358312	0.070413
N	1.839338	-0.875696	0.064022
C	4.547565	-0.170553	0.132308

C	2.642209	-1.096150	-0.996063
C	2.384673	-0.309098	1.159044
C	3.724393	0.057183	1.233888
C	3.993020	-0.760179	-1.002029
H	2.170043	-1.561982	-1.854627
H	1.718207	-0.166099	2.002737
H	4.107162	0.507991	2.143012
H	4.591840	-0.965350	-1.882862
H	5.598183	0.102320	0.158997
C	-0.712199	1.947439	-0.117754
C	0.395309	4.533321	-0.137558
C	-1.305932	2.986988	0.625054
C	0.457259	2.221763	-0.852121
C	0.995164	3.504830	-0.868891
C	-0.750855	4.264397	0.613316
H	-2.177216	2.780462	1.236143
H	0.918369	1.421078	-1.419917
H	1.887893	3.702563	-1.454845
H	-1.214468	5.052111	1.199898
H	0.820887	5.531932	-0.146800
Cl	-0.290937	-2.430265	-1.968609
Cl	-0.313780	-2.082142	2.245718
C	-2.605949	-1.127739	0.017029
N	-1.201587	0.635176	-0.104893
C	-2.512730	0.264157	-0.162504
C	-3.643893	1.219643	-0.464091
H	-4.404832	0.710887	-1.060691
H	-3.298161	2.100852	-1.009020
H	-4.132057	1.563561	0.455382
C	-3.881618	-1.888362	0.057396
H	-3.962150	-2.412140	1.019881
H	-3.864169	-2.681846	-0.701567
H	-4.795049	-1.294722	-0.081717

$TS2^T$

B3LYP/BS1 SCF energy: -2616.677319 a.u.

M06-L/BS2 SCF energy in solution: -2616.804095a.u.

M06-L/BS2 Free energy in solution: -2616.502843 a.u.

Ti	0.942213	-0.442786	-0.737379
N	1.236418	1.694351	-0.023609
C	1.588640	4.383543	0.647172
C	2.430826	2.147555	0.408675
C	0.221158	2.574477	-0.130163
C	0.355474	3.921420	0.189948
C	2.641955	3.477639	0.759721
H	3.225116	1.409908	0.445996
H	-0.725575	2.178510	-0.481122
H	-0.493868	4.586639	0.079862
H	3.621910	3.790515	1.103256
H	1.726438	5.428848	0.906734
C	-2.453742	-0.186544	-0.035292
C	-4.927984	1.150677	0.073914
C	-3.314943	-0.361508	1.063137
C	-2.847050	0.673880	-1.076185
C	-4.075795	1.329328	-1.018622
C	-4.540686	0.302719	1.112554
H	-3.010379	-1.009866	1.879092
H	-2.190191	0.788805	-1.932785
H	-4.371242	1.978751	-1.837818
H	-5.192522	0.157415	1.969418
H	-5.882629	1.666016	0.114909
Cl	0.363540	0.010971	-2.915852
Cl	3.294338	-0.801237	-0.876909
C	0.433797	-2.392957	-0.015185
N	-1.185890	-0.797703	-0.090105
C	-0.972362	-2.103322	0.018741
C	-2.055745	-3.154324	0.066409
H	-1.807584	-3.970731	-0.618263
H	-3.035269	-2.754868	-0.199392
H	-2.119609	-3.592441	1.070053
C	0.931185	-3.797856	-0.170694
H	0.541844	-4.206009	-1.115142
H	0.607964	-4.486471	0.624246
H	2.018822	-3.837712	-0.246534
C	0.695467	-0.522693	2.200871
C	1.276273	-1.542755	1.775143
C	-0.057736	0.583209	2.776869
H	-0.993301	0.772887	2.237561
H	0.526899	1.509330	2.766681

H	-0.316586	0.361352	3.819554
C	2.417122	-2.454235	2.086075
H	2.918046	-2.108108	2.993996
H	3.137468	-2.454314	1.265363
H	2.072823	-3.478689	2.251065

IM3^{T}

B3LYP/BS1 SCF energy: -2616.761993 a.u.
M06-L/BS2 SCF energy in solution: -2616.881209 a.u.
M06-L/BS2 Free energy in solution:
-2616.574806 a.u.

Ti	-0.428416	-0.262243	0.024901
N	-2.704513	-0.758143	-0.172620
C	-5.442097	-1.242939	-0.489436
C	-3.430506	-0.082876	-1.086069
C	-3.338268	-1.676524	0.581881
C	-4.697873	-1.949226	0.452204
C	-4.792483	-0.289783	-1.272930
H	-2.884586	0.629623	-1.694175
H	-2.719859	-2.193629	1.307417
H	-5.154287	-2.703963	1.083400
H	-5.324775	0.284288	-2.023529
H	-6.504279	-1.431720	-0.613038
C	2.560821	-0.763846	0.004660
C	4.310081	-2.946997	-0.047290
C	3.069870	-1.283813	1.201512
C	2.918093	-1.346867	-1.217278
C	3.790143	-2.436301	-1.237002
C	3.946527	-2.366959	1.169854
H	2.763189	-0.843183	2.143489
H	2.497107	-0.954357	-2.136269
H	4.060531	-2.885344	-2.188282
H	4.337865	-2.763990	2.101881
H	4.987640	-3.795336	-0.066667
Cl	-0.360720	-0.412536	-2.310538
Cl	-0.273868	-1.730499	1.846485
C	1.202263	2.725070	-0.084983
N	1.636836	0.337833	0.042099
C	2.079502	1.566569	-0.123807
C	3.560873	1.821517	-0.342353
H	3.790233	1.888415	-1.412601
H	4.157863	1.007507	0.065913
H	3.878553	2.756180	0.122868
C	1.777783	4.019086	-0.622373
H	2.510768	3.842824	-1.411192
H	2.277107	4.616571	0.154800
H	0.992777	4.647962	-1.046002
C	-0.808033	1.619382	0.894312
C	-0.085333	2.759993	0.530327
C	-2.030824	1.747363	1.776115
H	-2.347747	0.774663	2.159997
H	-2.887563	2.169806	1.229225
H	-1.855552	2.397484	2.643685
C	-0.662414	4.150816	0.791831
H	-1.499157	4.113026	1.487579
H	-1.037842	4.604010	-0.134474
H	0.080170	4.840282	1.205650

TS3^{T}

B3LYP/BS1 SCF energy: -2616.742733 a.u.
M06-L/BS2 SCF energy in solution: -2616.867268 a.u.
M06-L/BS2 Free energy in solution:
-2616.562698 a.u.

Ti	-0.372391	-0.358741	-0.618895
N	-2.621073	-0.503185	-0.125939
C	-5.320065	-0.616564	0.585240
C	-3.471240	0.480592	-0.477621
C	-3.110241	-1.550162	0.565038
C	-4.447356	-1.643045	0.940825
C	-4.821133	0.464230	-0.140599
H	-3.045306	1.285828	-1.066324
H	-2.399633	-2.336823	0.792731
H	-4.790625	-2.510943	1.493264
H	-5.463209	1.280697	-0.452730
H	-6.369555	-0.661545	0.860151
C	2.856074	-0.122647	-0.008935
C	5.477886	-1.046053	0.428427
C	3.821751	0.712468	0.589767

C	3.218610	-1.436336	-0.363730
C	4.516727	-1.885876	-0.141841
C	5.121168	0.254142	0.791909
H	3.542077	1.714818	0.900218
H	2.471574	-2.084366	-0.809034
H	4.781358	-2.900898	-0.423934
H	5.854969	0.913547	1.247148
H	6.490194	-1.402441	0.592517
Cl	-0.875268	0.582033	-2.713342
Cl	-0.218766	-2.686462	-0.784741
C	0.080452	2.060638	0.410495
N	1.545116	0.311664	-0.222330
C	1.182781	1.645190	-0.332600
C	1.853856	2.512972	-1.366108
H	1.247735	2.521440	-2.279819
H	2.836693	2.114533	-1.620701
H	1.964730	3.544265	-1.018624
C	-0.578487	3.397847	0.164244
H	-0.085817	4.210591	0.714412
H	-1.626789	3.390928	0.475588
H	-0.557061	3.658103	-0.896579
C	0.043341	-0.097634	1.509257
C	-0.304220	1.222523	1.548651
C	0.346890	-1.101281	2.559467
H	1.389077	-1.430011	2.445811
H	-0.269161	-2.001292	2.468324
H	0.242767	-0.692526	3.574066
C	-1.116251	1.823239	2.676226
H	-0.687309	2.776684	3.005325
H	-1.161063	1.147100	3.532488
H	-2.146012	2.025108	2.358006

IM4^{T}

B3LYP/BS1 SCF energy: -2616.802191 a.u.
M06-L/BS2 SCF energy in solution: -2616.929168 a.u.
M06-L/BS2 Free energy in solution: -2616.620250 a.u.

Ti	-0.590635	-0.075273	-0.584168
N	-2.761627	-0.230722	-0.338631
C	-5.539735	-0.455270	0.028364
C	-3.598465	0.791372	-0.644217
C	-3.321618	-1.373492	0.129327
C	-4.688929	-1.520763	0.326185
C	-4.974894	0.719081	-0.471362
H	-3.125821	1.673763	-1.060485
H	-2.632200	-2.187866	0.323054
H	-5.074319	-2.463232	0.700671
H	-5.590012	1.572543	-0.736464
H	-6.612169	-0.540842	0.172522
C	3.003704	-0.007902	0.173713
C	5.575250	-0.552054	-0.743931
C	4.093265	0.684054	0.712387
C	3.190921	-0.975565	-0.816388
C	4.483268	-1.244964	-1.268770
C	5.379018	0.412744	0.246191
H	3.929682	1.422798	1.490508
H	2.334294	-1.508438	-1.218557
H	4.631261	-1.995120	-2.039191
H	6.225753	0.951405	0.660436
H	6.577328	-0.762886	-1.104801
Cl	-0.775415	1.374445	-2.471521
Cl	-0.238335	-2.403281	-1.057294
C	-0.161594	1.451161	1.269998
N	1.681074	0.282471	0.664267
C	0.995339	1.495137	0.479858
C	1.582187	2.625879	-0.304380
H	0.789350	3.294018	-0.640977
H	2.103740	2.269057	-1.194444
H	2.291084	3.203747	0.301648
C	-1.125363	2.582336	1.495882
H	-0.860202	3.155487	2.393670
H	-2.146905	2.216737	1.630726
H	-1.130914	3.275163	0.650948
C	0.958507	-0.526451	1.551013
C	-0.180309	0.172500	1.940285
C	1.478794	-1.856825	1.988127
H	1.502123	-2.569965	1.159497
H	0.838194	-2.268743	2.769396
H	2.491821	-1.761759	2.393595
C	-1.203422	-0.264639	2.949733

H	-1.067037	0.263158	3.901465
H	-1.138811	-1.336069	3.152138
H	-2.220202	-0.057025	2.604669

TS4^{T}

B3LYP/BS1 SCF energy: -3189.575081 a.u.
M06-L/BS2 SCF energy in solution: -3189.758653 a.u.
M06-L/BS2 Free energy in solution:
-3189.276973 a.u.

Ti	-0.437585	-0.562180	0.414105
N	-2.387065	-1.562578	0.087943
C	-4.846637	-2.818808	-0.396276
C	-3.165093	-1.994799	1.108124
C	-2.845219	-1.742279	-1.173477
C	-4.057925	-2.363041	-1.451808
C	-4.387385	-2.624499	0.906863
H	-2.777317	-1.805740	2.103180
H	-2.207918	-1.361438	-1.964625
H	-4.372629	-2.478288	-2.483383
H	-4.965538	-2.952309	1.764287
H	-5.797334	-3.308552	-0.583451
C	3.689801	-0.879165	-0.436946
C	5.955235	0.680171	-0.889243
C	4.955427	-1.378084	-0.111074
C	3.551455	0.394808	-0.992624
C	4.690226	1.171115	-1.215188
C	6.087495	-0.595955	-0.337778
H	5.042453	-2.373193	0.314075
H	2.563429	0.759371	-1.253253
H	4.580883	2.163836	-1.640057
H	7.069875	-0.983450	-0.084905
H	6.837187	1.289069	-1.065248
Cl	-0.871738	-0.070595	2.769750
Cl	-0.033312	0.201035	-1.844929
C	0.857590	-2.769027	0.856479
N	2.535759	-1.701876	-0.196730
C	1.865209	-1.805175	1.022058
C	2.441232	-1.217974	2.277092
H	1.684668	-1.184181	3.061137
H	2.801706	-0.197785	2.118136
H	3.291327	-1.813159	2.635599
C	0.100192	-3.461212	1.961017
H	0.642008	-4.357766	2.291130
H	-0.887861	-3.789528	1.625717
H	-0.041396	-2.812470	2.827172
C	1.960437	-2.552706	-1.128197
C	0.925564	-3.228978	-0.508599
C	2.412314	-2.564385	-2.551429
H	2.064802	-1.665861	-3.074344
H	2.006801	-3.434530	-3.071366
H	3.503301	-2.600822	-2.630281
C	0.051975	-4.291721	-1.110252
H	0.179541	-5.253224	-0.597453
H	0.282805	-4.451089	-2.166276
H	-1.011184	-4.035028	-1.045316
N	-0.789796	2.539557	0.340784
N	-1.605156	3.210779	-0.341532
C	0.505944	3.124218	0.435496
C	3.067449	4.179235	0.772515
C	1.011806	4.039429	-0.500634
C	1.286315	2.729042	1.530497
C	2.561335	3.269012	1.701557
C	2.291187	4.557542	-0.329722
H	0.393779	4.319744	-1.345258
H	0.865639	2.028686	2.245928
H	3.158014	2.978720	2.561222
H	2.688255	5.260504	-1.056738
H	4.063708	4.592082	0.902127
C	-2.921505	2.692202	-0.401196
C	-5.579881	1.871303	-0.629034
C	-3.710204	3.149842	-1.465565
C	-3.473136	1.839283	0.569969
C	-4.801316	1.439473	0.450164
C	-5.030900	2.725237	-1.588465
H	-3.263645	3.828062	-2.185337
H	-2.866165	1.523883	1.412478
H	-5.235505	0.793407	1.207607
H	-5.635164	3.070190	-2.422197
H	-6.615948	1.555548	-0.711759

IM5^{T}

B3LYP/BS1 SCF energy: -2591.102435 a.u.

M06-L/BS2 SCF energy in solution: -2591.217325 a.u.

M06-L/BS2 Free energy in solution: -2590.990341 a.u.

Ti	0.638303	-1.245417	-0.473522
N	2.568519	-0.404909	0.250438
C	4.977568	0.720364	1.088156
C	3.263287	0.419739	-0.559470
C	3.067719	-0.680705	1.472008
C	4.263543	-0.133603	1.926759
C	4.468348	0.999028	-0.180114
H	2.833412	0.602163	-1.538675
H	2.486257	-1.370705	2.073900
H	4.624601	-0.384880	2.917854
H	4.991535	1.651776	-0.869889
H	5.916262	1.157775	1.414164
C	-0.603311	1.838189	-0.177305
C	-0.864643	4.628359	-0.336470
C	0.341231	2.678985	0.440763
C	-1.677552	2.410279	-0.886892
C	-1.796943	3.794786	-0.960676
C	0.204621	4.061309	0.362069
H	1.163562	2.231230	0.988840
H	-2.396450	1.769303	-1.383459
H	-2.624576	4.226272	-1.516049
H	0.934820	4.699459	0.851199
H	-0.968941	5.707054	-0.396807
Cl	1.253045	-1.800761	-2.598591
Cl	0.606423	-3.004954	1.046531
N	-0.387417	0.459432	-0.112101
C	-2.588205	-0.597074	0.105156
C	-3.161477	0.289976	1.039745
C	-3.354454	-1.673284	-0.387213
C	-4.475800	0.097465	1.454291
H	-2.576141	1.104180	1.448283
C	-4.667665	-1.845818	0.031284
H	-2.897242	-2.356937	-1.094982
C	-5.238355	-0.960300	0.951185
H	-4.906901	0.778353	2.182508
H	-5.248019	-2.677247	-0.357109
H	-6.263933	-1.098061	1.279112
N	-1.276722	-0.515891	-0.352061

TS10^{T}

B3LYP/BS1 SCF energy: -2772.783520 a.u.

M06-L/BS2 SCF energy in solution: -2772.921291 a.u.

M06-L/BS2 Free energy in solution: -2772.537447 a.u.

Ti	-0.796170	0.499453	0.271721
N	-2.678866	-0.517746	-0.121532
C	-5.115323	-1.802823	-0.648650
C	-3.612918	-0.673734	0.849183
C	-2.968516	-0.989706	-1.359416
C	-4.163440	-1.633815	-1.655807
C	-4.828927	-1.306803	0.624579
H	-3.352533	-0.262605	1.819203
H	-2.206303	-0.825784	-2.114641
H	-4.339671	-1.991851	-2.664498
H	-5.537442	-1.403276	1.440301
H	-6.056889	-2.303005	-0.851792
C	3.520035	-0.228803	-0.084761
C	5.923397	1.112328	-0.535168
C	4.686686	-0.653177	0.559369
C	3.550868	0.860595	-0.959599
C	4.757561	1.526668	-1.181237
C	5.886729	0.021418	0.335390
H	4.646484	-1.508629	1.226335
H	2.635125	1.166329	-1.455658
H	4.783030	2.372847	-1.861331
H	6.791541	-0.307982	0.837318
H	6.858506	1.635963	-0.710531
Cl	-1.217430	1.452216	2.445648
Cl	0.069734	0.896483	-1.950295
C	0.424893	-1.687350	1.154493
N	2.292658	-0.936245	0.155699
C	1.494436	-0.789650	1.283893
C	1.966973	-0.022418	2.482671

H	1.127545	0.214591	3.135856
H	2.444302	0.920674	2.201826
H	2.703274	-0.601796	3.055085
C	-0.498381	-2.133487	2.261564
H	-0.070744	-2.994928	2.791990
H	-1.470185	-2.450717	1.871702
H	-0.676128	-1.343118	2.993615
C	1.746965	-1.891248	-0.691706
C	0.599909	-2.381286	-0.098799
C	2.351742	-2.182884	-2.025923
H	2.145186	-1.373255	-2.735324
H	1.934820	-3.104281	-2.437730
H	3.437746	-2.304192	-1.961316
C	-0.280120	-3.485183	-0.610206
H	-0.250820	-4.360248	0.051406
H	0.028088	-3.817127	-1.604682
H	-1.329083	-3.177700	-0.681365
C	-1.675532	3.410833	-0.347861
C	-2.756047	2.940390	-0.633100
C	-0.408462	4.063911	-0.024671
H	-0.487953	5.147352	-0.168647
H	-0.133568	3.878432	1.018842
H	0.393073	3.694092	-0.672667
C	-4.090353	2.475217	-1.002433
H	-4.052862	1.771361	-1.840646
H	-4.589273	1.976871	-0.164565
H	-4.718267	3.320125	-1.306943

IM11^{T}

B3LYP/BS1 SCF energy: -2174.278826 a.u.
M06-L/BS2 SCF energy in solution: -2174.355110 a.u.
M06-L/BS2 Free energy in solution: -2174.226920 a.u.

Ti	-0.884521	-0.360790	-0.000821
N	1.308902	-0.069856	0.001130
C	4.092015	0.168918	0.006691
C	1.996850	-0.010777	1.161285
C	2.002972	-0.028965	-1.156111
C	3.387576	0.093283	-1.194360
C	3.381205	0.112407	1.205065
H	1.404247	-0.084381	2.067219
H	1.415042	-0.116351	-2.063838
H	3.897207	0.119994	-2.151210
H	3.885697	0.154351	2.164089
H	5.173608	0.262299	0.008833
Cl	-1.136123	-1.506753	2.012385
Cl	-1.130207	-1.518003	-2.008378
C	-3.755749	1.176064	0.007054
H	-4.069919	0.611693	0.890077
H	-4.265165	2.147586	0.016421
H	-4.081268	0.622799	-0.878921
C	-0.143535	2.967511	-0.023167
H	0.460415	2.931300	-0.934909
H	-0.634677	3.946628	0.022678
H	0.538473	2.886794	0.828368
C	-2.288917	1.361784	-0.001736
C	-1.155566	1.887192	-0.009182

TS11^{T}

B3LYP/BS1 SCF energy: -2330.240304 a.u.
M06-L/BS2 SCF energy in solution: -2330.333945 a.u.
M06-L/BS2 Free energy in solution: -2330.124812 a.u.

Ti	0.395158	-0.261621	-0.035904
N	-1.911289	-0.205251	-0.018176
C	-4.697973	0.051632	-0.007174
C	-2.604353	-0.105696	-1.169168
C	-2.602311	-0.183793	1.138442
C	-3.987691	-0.057506	1.186706
C	-3.989307	0.026264	-1.206649
H	-2.017596	-0.157287	-2.078956
H	-2.012481	-0.291264	2.041749
H	-4.491328	-0.051850	2.147325
H	-4.494211	0.099260	-2.163819
H	-5.779500	0.148917	-0.003016
Cl	0.253875	-1.506438	-2.043191
Cl	0.352359	-1.389784	2.037828
C	2.608874	0.177181	-0.593303

C	2.964177	-0.743246	0.197163
C	3.514284	-1.773809	1.065048
H	3.096581	-2.754165	0.814976
H	3.280149	-1.581993	2.118053
H	4.605288	-1.817305	0.949667
C	2.990639	0.688995	-1.955863
H	2.792125	1.758303	-2.058618
H	2.398420	0.160262	-2.708243
H	4.051166	0.502013	-2.148293
C	0.248293	1.813972	0.093254
C	1.529960	1.746730	0.267281
C	-0.773195	2.897761	0.268343
H	-1.547536	2.593539	0.979426
H	-1.280334	3.098495	-0.681930
H	-0.329526	3.836930	0.621906
C	2.668766	2.556476	0.783484
H	3.166155	2.044096	1.612861
H	2.295009	3.522722	1.143285
H	3.426212	2.747744	0.016523

IM12^{T}

B3LYP/BS1 SCF energy: -2330.295970 a.u.

M06-L/BS2 SCF energy in solution: -2330.385657 a.u.

M06-L/BS2 Free energy in solution: -2330.174852 a.u.

Ti	-0.243932	-0.702445	-0.023351
N	2.020950	-0.065559	0.178047
C	4.667827	0.836752	0.292782
C	2.477865	0.612410	1.249343
C	2.882172	-0.302995	-0.831825
C	4.205040	0.126998	-0.814165
C	3.785224	1.080916	1.343108
H	1.764324	0.757300	2.053274
H	2.487423	-0.870569	-1.667873
H	4.853759	-0.098619	-1.653601
H	4.098386	1.619724	2.230853
H	5.694752	1.186562	0.337723
Cl	-0.375931	-0.745483	2.320069
Cl	0.271840	-2.446463	-1.409602

C	-2.828407	0.591367	-0.006700
C	-2.359334	-0.735025	-0.109955
C	-3.249684	-1.931136	-0.034521
H	-3.211419	-2.334008	0.989247
H	-2.872146	-2.729039	-0.682569
H	-4.300943	-1.740732	-0.284048
C	-4.175033	0.972052	0.557899
H	-4.734084	1.616077	-0.132131
H	-4.057164	1.537259	1.491010
H	-4.788415	0.097322	0.777065
C	-0.683967	1.131934	-0.954064
C	-1.923051	1.595417	-0.470933
C	0.201420	1.994256	-1.806906
H	0.899275	1.392814	-2.397276
H	0.821802	2.648230	-1.174095
H	-0.353671	2.643673	-2.495619
C	-2.328710	3.051570	-0.456562
H	-3.187107	3.232380	-1.116821
H	-1.517817	3.706421	-0.778836
H	-2.635730	3.363879	0.548138

TS12^{T}

B3LYP/BS1 SCF energy: -2486.256627 a.u.

M06-L/BS2 SCF energy in solution: -2486.401280 a.u.

M06-L/BS2 Free energy in solution: -2486.108603 a.u.

Ti	0.015214	0.519912	-0.214789
C	-0.791896	0.656102	1.792013
C	-2.205752	0.827539	-0.427343
C	-3.059606	-0.258303	-0.234799
C	-0.376494	-0.551066	1.698979
C	-2.398140	-1.506841	-0.143755
C	-0.984127	-1.436335	-0.130926
C	-2.628200	2.174172	-0.913839
H	-2.137480	2.364646	-1.876945
H	-3.710181	2.282997	-1.059372
H	-2.267662	2.964438	-0.246103
C	-4.564961	-0.165325	-0.129025
H	-4.919246	0.865329	-0.169542

H	-5.051547	-0.717681	-0.943096
H	-4.925341	-0.605569	0.808542
C	-3.189650	-2.792408	-0.084984
H	-3.872069	-2.800989	0.774778
H	-3.816104	-2.910172	-0.978381
H	-2.554672	-3.676338	-0.014411
C	-0.161242	-2.669122	-0.391588
H	0.904900	-2.505645	-0.222563
H	-0.464548	-3.536352	0.211143
H	-0.285445	-2.950479	-1.444497
C	0.070908	-1.714380	2.525150
H	1.019087	-2.130195	2.172210
H	0.206583	-1.392615	3.563521
H	-0.665685	-2.524499	2.516766
C	-1.260702	1.753779	2.668294
H	-2.319134	1.965324	2.476856
H	-1.156645	1.489946	3.728667
H	-0.702572	2.672147	2.468311
Cl	0.043130	0.271395	-2.587454
Cl	0.731439	2.761755	0.027241
N	2.247012	-0.158528	-0.111420
C	2.924202	0.068993	1.030943
C	2.928212	-0.638552	-1.169431
C	4.284881	-0.182161	1.164136
H	2.347160	0.476182	1.854246
C	4.290750	-0.921038	-1.116708
H	2.348273	-0.774187	-2.076172
C	4.983608	-0.690325	0.069274
H	4.781028	0.024876	2.105987
H	4.791529	-1.305909	-1.998341
H	6.047561	-0.895562	0.138965

IM13^{T}

B3LYP/BS1 SCF energy: -2486.447949 a.u.

M06-L/BS2 SCF energy in solution: -2486.566728 a.u.

M06-L/BS2 Free energy in solution: -2486.262005 a.u.

Ti	0.191522	0.007165	-0.637050
C	2.447188	-0.747167	0.087745
C	2.480040	0.679826	0.077145
C	1.623007	1.404619	0.955662
C	1.559094	-1.418204	0.978870
C	0.603750	0.740624	1.682557
C	0.578953	-0.698855	1.701436
C	3.450029	1.453009	-0.788845
H	2.964074	2.329683	-1.220970
H	4.317497	1.791855	-0.207585
H	3.826802	0.858353	-1.618570
C	1.784643	2.906944	1.054224
H	2.832048	3.189840	0.937770
H	1.216095	3.415255	0.267506
H	1.454994	3.284616	2.022283
C	-0.379440	1.524278	2.528538
H	-0.093610	1.513672	3.588405
H	-0.446121	2.564279	2.212690
H	-1.387602	1.111757	2.460961
C	-0.403526	-1.439480	2.586143
H	-0.764523	-2.353604	2.111532
H	0.063819	-1.724434	3.537853
H	-1.277000	-0.835718	2.826178
C	1.647346	-2.924361	1.092261
H	1.179392	-3.407175	0.227312
H	2.691984	-3.243285	1.122426
H	1.166280	-3.295035	1.996565
C	3.388021	-1.583317	-0.750089
H	4.207713	-1.982973	-0.139036
H	2.855520	-2.424540	-1.198396
H	3.835738	-1.017329	-1.564107
Cl	0.003861	2.027868	-1.904422
Cl	-0.011539	-1.996865	-1.931027
N	-2.062072	0.005909	-0.369865
C	-2.751713	-1.149654	-0.252654
C	-2.756528	1.159420	-0.259215
C	-4.119879	-1.194160	-0.005334
H	-2.175120	-2.056553	-0.394155
C	-4.125052	1.199242	-0.012325
H	-2.182806	2.067630	-0.404052
C	-4.825281	0.001361	0.122860
H	-4.616987	-2.155134	0.073684
H	-4.626383	2.158456	0.061083

H	-5.894167	-0.000410	0.313540

TS13^{T}

B3LYP/BS1 SCF energy: -2642.422037 a.u.
M06-L/BS2 SCF energy in solution: -2642.552745 a.u.
M06-L/BS2 Free energy in solution: -2642.173410 a.u.

Ti	0.823991	-0.664041	0.023163
N	0.677003	1.498334	-0.022450
C	0.514299	4.295694	-0.072926
C	0.736174	2.222328	1.121675
C	0.545669	2.169580	-1.191974
C	0.460193	3.555018	-1.255035
C	0.657490	3.609572	1.134730
H	0.860180	1.648800	2.034793
H	0.520620	1.555044	-2.086296
H	0.357529	4.039377	-2.220140
H	0.713634	4.137795	2.080530
H	0.451107	5.379026	-0.092434
Cl	1.432768	-1.167610	2.291333
Cl	1.204308	-1.277775	-2.269107
C	-2.169489	-0.353800	1.463133
C	-1.780895	-1.614482	0.933507
C	-1.531741	-2.799061	1.852611
H	-1.608746	-2.527841	2.902733
H	-0.542188	-3.242034	1.719179
H	-2.276029	-3.579805	1.656467
C	-2.081637	-0.108825	2.957621
H	-2.947292	-0.519411	3.493880
H	-2.031315	0.955325	3.191697
H	-1.181397	-0.566401	3.371981
C	-2.673622	0.438656	-0.798644
C	-2.658933	0.645177	0.600758
C	-3.124783	1.563872	-1.712518
H	-2.651982	1.503534	-2.693494
H	-2.870072	2.541825	-1.299636
H	-4.210951	1.554335	-1.875147
C	-3.205847	1.943328	1.166342
H	-3.613964	1.806423	2.168474
H	-4.014565	2.336969	0.547601
H	-2.437942	2.726006	1.230616
C	3.804304	-0.988611	-0.125219
C	3.833234	0.222941	-0.187905
C	3.893803	-2.446901	-0.063527
H	4.941934	-2.763344	-0.108566
H	3.463699	-2.822250	0.870284
H	3.364706	-2.902111	-0.906934
C	4.012955	1.669805	-0.281724
H	3.498095	2.083873	-1.154882
H	3.636958	2.183789	0.608988
H	5.077299	1.911409	-0.379202
C	-2.274255	-0.800414	-1.331642
C	-2.395143	-1.109131	-2.808911
H	-1.406616	-1.197088	-3.273632
H	-2.908309	-2.063925	-2.961465
H	-2.963521	-0.354094	-3.350310
C	-1.512103	-3.197095	-1.040258
H	-0.919307	-3.809556	-0.360191
H	-2.442664	-3.746816	-1.237821
H	-0.960514	-3.121884	-1.977949
C	-1.815590	-1.826795	-0.464991

IM4NN^{T}

B3LYP/BS1 SCF energy: -2941.301661 a.u.
M06-L/BS2 SCF energy in solution: -2941.461546 a.u.
M06-L/BS2 Free energy in solution: -2941.060196 a.u.

Ti	0.112813	-0.258821	-0.448160
C	3.972130	0.062172	0.116507
C	6.427583	0.107635	-1.191842
C	5.086859	0.613126	0.754855
C	4.075737	-0.470298	-1.170891
C	5.310891	-0.447068	-1.819140
C	6.314740	0.638709	0.094408
H	4.987985	1.010595	1.760062
H	3.200162	-0.885227	-1.658075
H	5.394031	-0.857160	-2.820574
H	7.181747	1.067561	0.587257

H	7.384588	0.126531	-1.704271
Cl	0.851691	1.207852	-2.103563
Cl	0.480105	-2.369643	-1.335266
C	0.791812	0.707249	1.785484
N	2.713131	0.036684	0.813430
C	1.902648	1.144649	1.058849
C	2.317045	2.529751	0.682592
H	1.449408	3.191097	0.692123
H	2.747780	2.558748	-0.319881
H	3.054419	2.927854	1.389953
C	-0.234875	1.581606	2.449745
H	-1.203734	1.082610	2.513153
H	-0.387077	2.516560	1.906304
H	0.077711	1.834501	3.470436
C	2.120009	-1.103169	1.341393
C	0.925245	-0.719503	1.952978
C	2.785654	-2.438461	1.279709
H	2.771815	-2.849772	0.266349
H	2.266358	-3.143004	1.930860
H	3.826869	-2.367630	1.610063
C	0.039507	-1.596862	2.792744
H	0.099406	-2.642095	2.480148
H	-1.005113	-1.289567	2.715183
H	0.326956	-1.545729	3.850182
N	-1.728577	0.642706	-0.257653
N	-1.870247	-0.621332	0.133311
C	-2.633263	1.688911	-0.162289
C	-4.310410	3.936064	0.031988
C	-2.321072	2.877348	-0.858387
C	-3.792174	1.646265	0.644420
C	-4.613991	2.765404	0.733428
C	-3.158851	3.982082	-0.761186
H	-1.428725	2.896763	-1.475210
H	-4.033025	0.745372	1.196143
H	-5.500126	2.723277	1.360609
H	-2.913054	4.886616	-1.310139
H	-4.961252	4.801803	0.105106
C	-2.989650	-1.431803	-0.104832
C	-5.130775	-3.203408	-0.525335
C	-3.026136	-2.679020	0.546397
C	-4.032045	-1.090796	-0.989434
C	-5.087746	-1.975701	-1.190739
C	-4.089699	-3.550584	0.339805
H	-2.208658	-2.948897	1.205592
H	-4.002121	-0.150151	-1.525326
H	-5.881031	-1.704577	-1.881542
H	-4.103374	-4.508297	0.851967
H	-5.959556	-3.885470	-0.687842

IM4CC^{T}

B3LYP/BS1 SCF energy: -2524.479179 a.u.

M06-L/BS2 SCF energy in solution: -2524.593798 a.u.

M06-L/BS2 Free energy in solution: -2524.291001 a.u.

Ti	1.135883	-0.099688	-0.571634
C	-2.596782	0.043576	0.225053
C	-5.142591	-0.229238	-0.869182
C	-3.692286	0.636704	0.860131
C	-2.765782	-0.691604	-0.951119
C	-4.045258	-0.826374	-1.491394
C	-4.965009	0.502706	0.306367
H	-3.544232	1.190857	1.781628
H	-1.907910	-1.152358	-1.431102
H	-4.178490	-1.395653	-2.405877
H	-5.816117	0.964879	0.796990
H	-6.134432	-0.334294	-1.298222
Cl	0.634915	-2.284059	-1.363317
Cl	0.730683	1.456445	-2.292320
C	0.601079	-0.265845	1.948157
N	-1.289696	0.191628	0.808600
C	-0.583733	-0.817233	1.463936
C	-1.155429	-2.186344	1.639079
H	-0.545013	-2.757222	2.340648
H	-1.180588	-2.734394	0.692875
H	-2.173727	-2.132440	2.037773
C	1.600281	-0.936715	2.848908
H	1.411909	-0.686448	3.900415
H	2.620166	-0.619359	2.616603
H	1.561937	-2.024552	2.755758
C	-0.547249	1.376873	0.842886

C	0.622370	1.124777	1.563289
C	-1.083570	2.661749	0.299045
H	-1.577086	2.512652	-0.662691
H	-0.267274	3.367295	0.140307
H	-1.800419	3.116624	0.993295
C	1.630350	2.145021	2.015398
H	1.417901	2.476773	3.039596
H	1.620591	3.031563	1.376521
H	2.644505	1.737296	2.006916
C	3.385139	-0.743749	-0.269023
C	3.421511	0.453714	-0.587704
C	3.822871	-2.119858	0.035684
H	3.524356	-2.425853	1.043038
H	3.377150	-2.827107	-0.667972
H	4.915682	-2.188245	-0.030013
C	3.935166	1.776872	-0.991412
H	3.617704	2.561371	-0.297824
H	5.031806	1.764263	-1.014335
H	3.558383	2.045359	-1.981340

IM4PhCF$_3$^{T}

B3LYP/BS1 SCF energy: -2937.782985 a.u.

M06-L/BS2 SCF energy in solution: -2937.965750 a.u.

M06-L/BS2 Free energy in solution: -2937.651175 a.u.

Ti	0.447283	-0.115173	-0.477560
C	3.857716	0.089349	0.040371
C	6.455397	-0.538374	-0.727158
C	4.926100	0.841437	0.538397
C	4.074552	-0.976721	-0.833091
C	5.382182	-1.287363	-1.211057
C	6.226662	0.526126	0.147737
H	4.735073	1.661575	1.223629
H	3.230634	-1.555530	-1.195925
H	5.556417	-2.116544	-1.889447
H	7.059056	1.109664	0.528764
H	7.469054	-0.782954	-1.029604
Cl	-0.682516	1.072045	-2.184361
Cl	0.734793	-2.440612	-0.834655
C	0.768628	1.778525	0.997970
N	2.515932	0.431237	0.458457
C	1.861082	1.640161	0.123881
C	2.397060	2.585915	-0.902904
H	1.571206	3.132284	-1.362012
H	2.926011	2.055853	-1.697929
H	3.091011	3.312473	-0.461168
C	-0.184338	2.938807	1.028829
H	0.227867	3.773265	1.610036
H	-1.135068	2.655561	1.486628
H	-0.400403	3.298385	0.020030
C	1.853513	-0.161463	1.558128
C	0.771853	0.651538	1.893233
C	2.377700	-1.406769	2.195818
H	2.472096	-2.218598	1.470583
H	1.695895	-1.736665	2.980981
H	3.359557	-1.228432	2.649417
C	-0.202411	0.422379	3.013076
H	0.017752	1.080219	3.862089
H	-0.168345	-0.609013	3.369755
H	-1.227346	0.623770	2.692950
C	-4.275632	-0.423497	-0.038105
C	-6.490248	1.243530	-0.280451
C	-4.270218	0.597998	-0.995762
C	-5.379598	-0.614149	0.795419
C	-6.488736	0.222965	0.670842
C	-5.381792	1.429821	-1.110789
H	-3.402335	0.739468	-1.633689
H	-5.371157	-1.412677	1.528547
H	-7.350064	0.075197	1.315080
H	-5.382554	2.223264	-1.851703
H	-7.355019	1.893638	-0.376372
C	-3.070308	-1.311921	0.076380
F	-3.225606	-2.296921	0.979450
F	-1.965197	-0.582558	0.500738
F	-2.721953	-1.863097	-1.091836

Pyr^{2-}

B3LYP/BS1 SCF energy: -598.244917 a.u.

M06-L/BS2 SCF energy in solution: not available

M06-L/BS2 Free energy in solution: not available

C	-1.243768	-0.000221	-0.000173
C	-4.157909	0.000191	-0.000115
C	-2.025099	-1.047683	0.645823
C	-2.024855	1.047427	-0.646202
C	-3.404131	1.018061	-0.654981
C	-3.404366	-1.017953	0.654651
C	2.307634	-0.687948	0.073936
N	0.117970	-0.000066	0.000179
C	0.944245	-1.178902	0.164729
C	0.526591	-2.399931	-0.609554
C	3.499376	-1.596790	0.074299
C	0.944034	1.178987	-0.164276
C	2.307533	0.688237	-0.073645
C	0.526258	2.399720	0.610442
C	3.499337	1.596968	-0.074992
H	-1.485816	-1.799049	1.217297
H	-1.485285	1.798375	-1.217984
H	-3.935328	1.807997	-1.198602
H	-3.935751	-1.807858	1.198126
H	-5.247283	0.000415	0.000041
H	1.329166	-3.153692	-0.580066
H	0.309082	-2.206672	-1.693739
H	-0.386831	-2.893307	-0.233909
H	4.448843	-1.042737	0.084721
H	3.547390	-2.279015	-0.801165
H	3.500013	-2.268675	0.954463
H	-0.388121	2.892005	0.235786
H	1.328040	3.154291	0.579751
H	0.310407	2.206613	1.695002
H	3.545182	2.282551	0.797928
H	3.502561	2.265593	-0.957700
H	4.448663	1.042647	-0.080842

Pyr

B3LYP/BS1 SCF energy: -598.501707 a.u.

M06-L/BS2 SCF energy in solution: not available

M06-L/BS2 Free energy in solution: not available

C	-1.260361	0.000012	-0.000080
C	-4.059940	-0.000058	0.000281
C	-1.964001	-0.541488	1.082532
C	-1.964314	0.541489	-1.082518
C	-3.359451	0.546713	-1.076909
C	-3.359155	-0.546784	1.077283
C	2.284877	-0.714596	-0.073256
N	0.165237	0.000043	-0.000280
C	0.970445	-1.133098	-0.112388
C	0.385935	-2.499358	-0.289676
C	3.515497	-1.568680	-0.167783
C	0.970445	1.133137	0.112122
C	2.284879	0.714610	0.073310
C	0.385948	2.499437	0.289121
C	3.515515	1.568627	0.168208
H	-1.409996	-0.944952	1.924181
H	-1.410523	0.944984	-1.924292
H	-3.898892	0.969556	-1.919549
H	-3.898334	-0.969660	1.920074
H	-5.145979	-0.000084	0.000404
H	1.178375	-3.215579	-0.518183
H	-0.337309	-2.531682	-1.113393
H	-0.134531	-2.862773	0.605331
H	4.131642	-1.496889	0.738130
H	4.155663	-1.268502	-1.007381
H	3.270668	-2.625379	-0.307762
H	-0.134454	2.862673	-0.605998
H	1.178387	3.215696	0.517513
H	-0.337347	2.531966	1.112786
H	4.155030	1.268907	1.008471
H	3.270700	2.625440	0.307344
H	4.132324	1.496204	-0.737198

HMB^{2-}

B3LYP/BS1 SCF energy: -467.858265 a.u.

M06-L/BS2 SCF energy in solution: -468.194210 a.u.

M06-L/BS2 Free energy in solution:
-467.982014 a.u.

C	-1.246682	-0.690007	-0.111332
C	0.001081	-1.483887	-0.087206
C	1.247252	-0.688892	-0.110389
C	-1.247258	0.688922	-0.110438
C	1.246715	0.690032	-0.111322
C	-0.001085	1.483995	-0.087324
C	0.000960	-2.733842	0.769805
H	0.878286	-3.375427	0.579832
H	-0.000850	-2.608938	1.914058
H	-0.874853	-3.376871	0.577491
C	2.512616	-1.510046	-0.302621
H	2.798645	-2.082865	0.599572
H	2.337271	-2.275590	-1.087899
H	3.401622	-0.927676	-0.582275
C	2.509986	1.512683	-0.309220
H	2.797569	2.089440	0.590092
H	3.399537	0.931314	-0.589413
H	2.331017	2.275463	-1.096527
C	-0.001006	2.733760	0.770085
H	-0.878108	3.375622	0.579945
H	0.000377	2.608508	1.914226
H	0.875012	3.376625	0.578172
C	-2.512594	1.510038	-0.302806
H	-2.337577	2.274978	-1.088785
H	-3.401915	0.927613	-0.581349
H	-2.797958	2.083718	0.599054
C	-2.509980	-1.512705	-0.309043
H	-2.797712	-2.089141	0.590423
H	-3.399412	-0.931366	-0.589628
H	-2.330974	-2.275701	-1.096122

HMB

B3LYP/BS1 SCF energy: -468.161307 a.u.
M06-L/BS2 SCF energy in solution: -468.190387 a.u.
M06-L/BS2 Free energy in solution:
-467.964689 a.u

C	-1.073411	-0.911782	-0.010255
C	-1.326207	0.473672	0.010342
C	-0.252868	1.385475	-0.010094
C	1.073427	0.911776	0.010235
C	1.326232	-0.473693	-0.010377
C	0.252878	-1.385497	0.010103
C	-0.525208	2.877327	-0.076677
H	-0.603533	3.333165	0.920266
H	0.266207	3.407685	-0.609951
H	-1.457585	3.093593	-0.601820
C	0.525130	-2.877361	0.076788
H	0.603004	-3.333332	-0.920139
H	1.457681	-3.093663	0.601586
H	-0.266096	-3.407588	0.610481
C	-2.229512	-1.893317	-0.076829
H	-1.950858	-2.809195	-0.601596
H	-2.585455	-2.188580	0.920138
H	-3.084312	-1.473029	-0.610424
C	-2.754342	0.983978	0.076889
H	-3.187996	1.144479	-0.920108
H	-3.408097	0.284627	0.601664
H	-2.817968	1.934371	0.610501
C	2.229514	1.893323	0.076842
H	2.585197	2.188884	-0.920128
H	1.950957	2.809038	0.601953
H	3.084449	1.472910	0.610118
C	2.754390	-0.983925	-0.076986
H	3.407943	-0.284821	-0.602345
H	3.188323	-1.143792	0.919991
H	2.818000	-1.934602	-0.610080

TS1a

B3LYP/BS1 SCF energy: -2709.011926 a.u.
M06-L/BS2 SCF energy in solution: -2709.167641 a.u.
M06-L/BS2 Free energy in solution:
-2708.861596 a.u.

Ti	-0.358748	-0.585523	-0.111067
N	-2.525376	0.128504	-0.191029
C	-5.146714	1.056797	-0.436008

C	-2.867006	1.356911	0.237828
C	-3.478430	-0.643239	-0.747089
C	-4.796521	-0.214832	-0.882499
C	-4.159408	1.858897	0.135303
H	-2.066779	1.945084	0.671249
H	-3.154125	-1.622760	-1.078810
H	-5.526502	-0.875727	-1.336711
H	-4.379194	2.858174	0.495351
H	-6.166273	1.417852	-0.532414
N	1.676457	-1.595079	-0.198535
C	4.198947	-2.773777	-0.356362
C	2.567005	-1.191311	-1.123311
C	2.025043	-2.587474	0.639297
C	3.276966	-3.193808	0.600393
C	3.832913	-1.757612	-1.237093
H	2.232261	-0.407153	-1.791993
H	1.258948	-2.912911	1.332160
H	3.509256	-3.989242	1.299931
H	4.509549	-1.404438	-2.007451
H	5.179894	-3.235165	-0.420013
C	1.223586	2.096871	0.001846
C	2.823963	4.397642	-0.308476
C	2.598181	2.072402	0.329759
C	0.673008	3.300681	-0.490544
C	1.468391	4.432560	-0.645485
C	3.382782	3.212189	0.176510
H	3.033576	1.147713	0.694526
H	-0.375800	3.315255	-0.767964
H	1.028850	5.346815	-1.034206
H	4.437999	3.174648	0.432662
H	3.440443	5.283160	-0.429027
Cl	-0.509991	-0.548956	-2.479992
Cl	-1.147329	-2.951972	-0.097408
C	-0.732186	-0.690320	2.019766
N	0.430724	0.973105	0.096566
C	-0.062808	0.387922	2.046603
C	0.514930	1.496194	2.832936
H	0.250382	1.328883	3.882956
H	0.123672	2.469827	2.523780
H	1.603508	1.543752	2.752663
C	-1.484350	-1.612027	2.913696
H	-1.249144	-2.651948	2.677062
H	-2.557994	-1.496153	2.730343
H	-1.288022	-1.412086	3.973743

TS1b

B3LYP/BS1 SCF energy: -2709.012483 a.u.

M06-L/BS2 SCF energy in solution: -2709.166618 a.u.

M06-L/BS2 Free energy in solution: -2708.862161 a.u.

Ti	0.043247	-0.600602	-0.102993
N	0.572283	1.748082	-0.088595
C	0.962350	4.521357	-0.197753
C	1.281128	2.312257	-1.085016
C	0.056144	2.563653	0.849498
C	0.222909	3.944974	0.833955
C	1.501656	3.685274	-1.171666
H	1.655483	1.628765	-1.837975
H	-0.502088	2.078076	1.640878
H	-0.221671	4.548746	1.617915
H	2.081138	4.080465	-1.998974
H	1.111797	5.596050	-0.241204
N	2.565528	-0.690091	-0.084595
C	5.351684	-0.947954	-0.218088
C	3.137378	-1.699142	-0.766445
C	3.378070	0.188862	0.523871
C	4.767591	0.103473	0.486351
C	4.518274	-1.863981	-0.855561
H	2.453909	-2.389068	-1.246800
H	2.891071	0.998507	1.057006
H	5.369957	0.846195	0.998947
H	4.922038	-2.698396	-1.418804
H	6.431764	-1.049091	-0.269562
C	-3.027312	-0.257559	-0.252015
C	-5.790638	-0.149994	-0.743316
C	-3.705092	0.979748	-0.264882
C	-3.757642	-1.441205	-0.492553
C	-5.126226	-1.380117	-0.738428
C	-5.075126	1.027284	-0.507982
H	-3.136760	1.889063	-0.095251

H	-3.221833	-2.384850	-0.494999
H	-5.678140	-2.295807	-0.930388
H	-5.586814	1.985574	-0.520994
H	-6.858470	-0.108237	-0.936318
Cl	0.142686	-2.946862	0.027152
Cl	0.439919	-0.487646	-2.447852
C	0.210397	-0.490206	2.059209
N	-1.667362	-0.323738	-0.044149
C	-1.053355	-0.404342	2.002903
C	-2.375321	-0.430680	2.651670
H	-2.214699	-0.474361	3.735062
H	-2.949211	-1.310505	2.347348
H	-2.977994	0.452414	2.422259
C	1.283770	-0.702120	3.072885
H	2.013913	0.112755	3.059913
H	1.826580	-1.623972	2.843327
H	0.877339	-0.779703	4.087912

TS2a

B3LYP/BS1 SCF energy: -2616.692560 a.u.
M06-L/BS2 SCF energy in solution: -2616.830823 a.u.
M06-L/BS2 Free energy in solution: -2616.527940 a.u.

Ti	0.625982	-0.991254	-0.320948
C	-2.252584	0.591209	-0.088131
C	-3.936699	2.809915	-0.535744
C	-3.590530	0.583208	0.351440
C	-1.771152	1.741244	-0.737544
C	-2.606751	2.834523	-0.958761
C	-4.422174	1.677373	0.119643
H	-3.975544	-0.276565	0.887764
H	-0.749103	1.753043	-1.096361
H	-2.215387	3.706531	-1.474873
H	-5.452339	1.645412	0.462981
H	-4.585942	3.661945	-0.712526
Cl	0.444609	-0.397718	-2.576643
Cl	2.661713	-2.075913	-0.430076
C	-0.471875	-2.586540	-0.046142
N	-1.363483	-0.472467	0.193890
C	-1.610371	-1.814359	-0.124435
C	-2.959117	-2.291347	-0.610006
H	-2.851483	-3.249900	-1.119787
H	-3.408170	-1.575292	-1.302694
C	-0.345071	-4.066877	-0.204444
H	-0.367010	-4.559711	0.776948
H	-1.153475	-4.506601	-0.803916
C	-0.414866	-0.345852	2.120189
C	0.667873	-0.973372	1.959329
C	-1.413304	0.442191	2.851829
H	-1.030864	0.592498	3.869902
H	-1.582145	1.426149	2.404854
C	1.820106	-1.452470	2.781527
H	2.736719	-0.937041	2.481253
H	1.994306	-2.516137	2.599762
H	0.606590	-4.329712	-0.672978
H	-3.657088	-2.434710	0.223295
N	1.833415	1.126078	0.005734
C	2.788344	1.375041	-0.912155
C	1.635102	2.043189	0.967493
C	3.564968	2.530137	-0.897527
H	2.921916	0.619661	-1.676967
C	2.360845	3.228130	1.051903
H	0.871788	1.803920	1.698447
C	3.347820	3.477773	0.100476
H	4.321969	2.675119	-1.660429
H	2.152387	3.932340	1.850184
H	3.935389	4.390211	0.137057
H	-2.381331	-0.062942	2.918772
H	1.656844	-1.292870	3.853392

TS2b

B3LYP/BS1 SCF energy: -2616.706689 a.u.
M06-L/BS2 SCF energy in solution: -2616.847931 a.u.
M06-L/BS2 Free energy in solution: -2616.544834 a.u.

Ti	-0.310584	-0.108631	-0.347529
N	-2.621830	-0.352940	0.032504
C	-5.399941	-0.608654	0.194344

C	-3.383671	0.645583	0.511297
C	-3.236345	-1.481974	-0.369969
C	-4.616322	-1.649071	-0.299530
C	-4.768897	0.563832	0.606494
H	-2.855522	1.535405	0.831568
H	-2.587684	-2.261981	-0.751045
H	-5.058779	-2.581259	-0.632937
H	-5.333028	1.403716	0.997198
H	-6.479334	-0.708169	0.256786
C	2.890099	-0.144604	-0.067654
C	5.494341	-1.085661	0.425297
C	3.831092	0.664220	0.598119
C	3.268741	-1.434960	-0.474926
C	4.559197	-1.894685	-0.225158
C	5.121955	0.195469	0.835072
H	3.537067	1.652117	0.940119
H	2.541673	-2.061199	-0.977236
H	4.837016	-2.893874	-0.548275
H	5.835086	0.832669	1.350792
H	6.499528	-1.450623	0.613258
Cl	-0.893999	-0.295493	-2.588322
Cl	-0.021476	-2.441907	0.034950
C	-0.067572	1.912411	-0.431358
N	1.585585	0.315186	-0.291642
C	1.247768	1.602483	-0.701976
C	2.137360	2.373138	-1.651068
H	1.871804	2.102260	-2.680404
H	3.192114	2.138425	-1.502316
H	1.989394	3.450116	-1.538903
C	-0.802886	3.086578	-0.998901
H	-1.051866	2.902280	-2.053456
H	-0.225478	4.019783	-0.950778
H	-1.753636	3.266832	-0.485504
C	-0.386361	0.030832	2.091360
C	-0.443896	1.258112	1.940595
C	-0.363680	-1.184214	2.934773
H	0.567153	-1.735985	2.787678
H	-1.174788	-1.864083	2.665047
H	-0.459605	-0.908418	3.991001
C	-0.518709	2.682299	2.301393
H	-0.681916	2.751076	3.384514
H	-1.340307	3.204994	1.804215
H	0.407093	3.209081	2.056355

TS2c

B3LYP/BS1 SCF energy: -2616.690190 a.u.

M06-L/BS2 SCF energy in solution: -2616.829158 a.u.

M06-L/BS2 Free energy in solution: -2616.523629 a.u.

Ti	0.383517	-0.212817	0.020194
C	-2.781887	-0.005756	0.196891
C	-5.335328	-1.188784	0.207291
C	-3.940377	0.789569	0.262093
C	-2.924126	-1.400979	0.109966
C	-4.190719	-1.982333	0.117809
C	-5.202679	0.198686	0.277708
H	-3.854674	1.869429	0.286219
H	-2.039565	-2.025330	0.052440
H	-4.278358	-3.062984	0.058416
H	-6.085218	0.829324	0.334735
H	-6.320269	-1.645483	0.216379
Cl	0.262865	-1.669668	1.844829
Cl	0.752325	-1.927809	-1.583970
C	0.415067	1.560960	0.982738
N	-1.481414	0.558474	0.124516
C	-0.951418	1.503760	1.045439
C	-1.849377	2.246050	2.004959
H	-1.256633	2.685941	2.807863
H	-2.591115	1.578687	2.451395
C	1.286868	2.463486	1.798261
H	1.695050	3.279815	1.187025
H	0.758391	2.935359	2.635937
C	-0.928848	1.096636	-1.841999
C	0.337402	1.097393	-1.820349
C	-2.214339	1.093358	-2.551561
H	-2.005620	1.370585	-3.592643
H	-2.669546	0.098429	-2.557475
C	1.419443	1.401913	-2.805219
H	2.024356	0.505637	-2.971586
H	2.085134	2.188448	-2.434206

H	2.141636	1.921859	2.216323
H	-2.389128	3.060384	1.506782
N	2.696156	-0.197242	0.195244
C	3.438735	0.890109	-0.076893
C	3.338745	-1.339111	0.512007
C	4.829884	0.884233	-0.049852
H	2.888368	1.789400	-0.320216
C	4.726050	-1.426839	0.570182
H	2.709335	-2.194660	0.721892
C	5.489267	-0.297391	0.282069
H	5.375740	1.792113	-0.282479
H	5.189517	-2.370965	0.834079
H	6.573815	-0.336779	0.315699
H	-2.941367	1.800091	-2.142967
H	1.017182	1.731384	-3.770035

$TiCl_2Py$

B3LYP/BS1 SCF energy: -2018.229356 a.u.

M06-L/BS2 SCF energy in solution: -2018.295213 a.u.

M06-L/BS2 Free energy in solution: -2018.241903 a.u.

Ti	1.136183	0.000011	0.117956
N	-0.878634	0.000221	-0.239679
C	-3.660467	-0.000035	0.115684
C	-1.574796	1.170746	-0.125105
C	-1.574328	-1.170376	-0.123022
C	-2.948916	-1.201118	0.042097
C	-2.949350	1.201239	0.039945
H	-0.991158	2.081285	-0.209053
H	-0.990385	-2.080857	-0.205476
H	-3.452829	-2.159177	0.107036
H	-3.453672	2.159194	0.103180
H	-4.738548	-0.000115	0.234540
Cl	2.089438	2.030072	-0.018299
Cl	2.088688	-2.030358	-0.019648

$TiCl_2Py^T$

B3LYP/BS1 SCF energy: -2018.260745 a.u.

M06-L/BS2 SCF energy in solution: -2018.323004 a.u.

M06-L/BS2 Free energy in solution: -2018.270759 a.u.

Ti	1.262323	0.000005	-0.000023
N	-0.866786	0.000000	-0.000378
C	-3.666413	-0.000044	0.000086
C	-1.565859	-1.163736	-0.000215
C	-1.565894	1.163713	-0.000097
C	-2.952774	1.199509	0.000172
C	-2.952735	-1.199576	0.000054
H	-0.974297	-2.073427	-0.000276
H	-0.974362	2.073422	-0.000070
H	-3.459455	2.158213	0.000492
H	-3.459388	-2.158295	0.000280
H	-4.751464	-0.000062	0.000271
Cl	2.004096	-2.164083	0.000107
Cl	2.003987	2.164133	0.000037

$TiCl_2(Py)_4$

B3LYP/BS1 SCF energy: -2763.220983 a.u.

M06-L/BS2 SCF energy in solution: -2763.372299 a.u.

M06-L/BS2 Free energy in solution: -2763.067944 a.u.

Ti	0.000002	0.000000	-0.000488
Cl	-0.000025	-0.000069	-2.403828
Cl	0.000043	0.000063	2.403242
N	-1.602874	-1.531009	-0.000132
N	-1.531010	1.602871	-0.000220
N	1.602875	1.531013	-0.000262
N	1.531013	-1.602876	-0.000174
C	-1.672807	-2.491465	-0.958242
H	-0.901413	-2.453116	-1.717390
C	-2.666026	-3.460521	-0.995681
H	-2.660075	-4.193113	-1.796137
C	-3.643452	-3.481267	0.000751
H	-4.427998	-4.231241	0.001187
C	-3.578269	-2.505092	0.996525

H	-4.309616	-2.465515	1.797155
C	-2.565294	-1.556711	0.958192
H	-2.491887	-0.786991	1.716488
C	-1.556738	2.565325	0.958070
H	-0.787034	2.491948	1.716385
C	-2.505125	3.578296	0.996347
H	-2.465568	4.309671	1.796953
C	-3.481281	3.643439	0.000552
H	-4.231259	4.427981	0.000945
C	-3.460508	2.665978	-0.995845
H	-4.193084	2.659993	-1.796316
C	-2.491449	1.672765	-0.958352
H	-2.453080	0.901345	-1.717472
C	2.565327	1.556768	0.958028
H	2.491949	0.787085	1.716366
C	3.578297	2.505157	0.996281
H	4.309671	2.465624	1.796889
C	3.643440	3.481284	0.000457
H	4.427982	4.231263	0.000830
C	2.665981	3.460482	-0.995941
H	2.659998	4.193035	-1.796433
C	1.672769	2.491422	-0.958421
H	0.901350	2.453031	-1.717541
C	2.491440	-1.672809	-0.958312
H	2.453067	-0.901417	-1.717461
C	3.460496	-2.666026	-0.995777
H	4.193065	-2.660075	-1.796255
C	3.481274	-3.643450	0.000656
H	4.231250	-4.427995	0.001072
C	2.505128	-3.578267	0.996459
H	2.465576	-4.309612	1.797092
C	1.556744	-2.565293	0.958151
H	0.787046	-2.491885	1.716470

$TiCl_2(Py)_4^T$

B3LYP/BS1 SCF energy: -2763.240076 a.u.

M06-L/BS2 SCF energy in solution: -2763.383410 a.u.

M06-L/BS2 Free energy in solution: -2763.082325 a.u.

Ti	-0.000064	0.000163	0.000375
Cl	0.000431	-0.000119	2.501110
Cl	0.000589	0.000274	-2.502379
N	-2.280794	0.111904	0.001391
N	-0.111705	-2.267432	-0.000028
N	2.281038	-0.111986	0.001412
N	0.111626	2.267829	0.000156
C	-2.958705	0.621807	1.055150
H	-2.348685	0.948401	1.889732
C	-4.345948	0.707262	1.091775
H	-4.830458	1.126535	1.967232
C	-5.085053	0.251677	0.000407
H	-6.169532	0.305852	-0.000021
C	-4.394182	-0.275392	-1.090357
H	-4.917369	-0.644490	-1.966144
C	-3.005335	-0.328424	-1.052681
H	-2.430229	-0.714632	-1.886524
C	0.376819	-2.996567	-1.029353
H	0.796746	-2.424700	-1.849109
C	0.328244	-4.385842	-1.065780
H	0.739610	-4.911550	-1.921203
C	-0.254823	-5.074359	-0.002155
H	-0.310332	-6.158810	-0.003114
C	-0.764532	-4.331766	1.062796
H	-1.227171	-4.814202	1.917528
C	-0.671372	-2.944734	1.028524
H	-1.030352	-2.334485	1.849620
C	3.005750	0.329701	-1.051906
H	2.430853	0.717344	-1.885217
C	4.394598	0.276251	-1.089558
H	4.918024	0.646421	-1.964748
C	5.085144	-0.252713	0.000484
H	6.169605	-0.307284	0.000073
C	4.345815	-0.709693	1.091108
H	4.830136	-1.130385	1.965987
C	2.958595	-0.623718	1.054449
H	2.348351	-0.951387	1.888442
C	0.671252	2.945139	1.028718
H	1.030626	2.334847	1.849612
C	0.763928	4.332207	1.063199
H	1.226534	4.814659	1.917941

C	0.253803	5.074798	-0.001545
H	0.308931	6.159268	-0.002331
C	-0.329158	4.386257	-1.065212
H	-0.740806	4.911961	-1.920504
C	-0.377257	2.996965	-1.029006
H	-0.797073	2.425069	-1.848801

C_2H_4-$TiCl_2Py^S$

B3LYP/BS1 SCF energy: -2096.897929 a.u.
M06-L/BS2 SCF energy in solution: -2096.968597 a.u.
M06-L/BS2 Free energy in solution: -2096.864771 a.u.

Ti	-1.167710	0.000056	0.009964
N	1.053539	-0.000695	0.037425
C	3.852860	-0.000017	0.000911
C	1.750542	-1.157677	0.022899
C	1.750079	1.156639	0.027736
C	3.139216	1.197292	0.011099
C	3.139746	-1.197626	0.006327
H	1.163107	-2.068980	0.019351
H	1.162296	2.067730	0.028126
H	3.643867	2.157076	0.003471
H	3.644804	-2.157159	-0.005137
H	4.938126	0.000251	-0.012296
Cl	-1.506871	-2.022197	-0.956020
Cl	-1.505070	2.017343	-0.966682
C	-2.354370	0.005219	1.735687
C	-0.967543	0.006351	2.112799
H	-0.510107	-0.903839	2.501984
H	-0.510084	0.918975	2.496270
H	-2.938643	-0.908795	1.836930
H	-2.938705	0.919800	1.831310

C_2H_4-$TiCl_2Py^T$

B3LYP/BS1 SCF energy: -2096.882492 a.u.
M06-L/BS2 SCF energy in solution: -2096.945677 a.u.
M06-L/BS2 Free energy in solution: -2096.845452 a.u.

Ti	-1.095594	0.000871	-0.019704
N	1.103503	0.000050	0.072850
C	3.895608	-0.001437	0.022984
C	1.795468	-1.159957	0.051108
C	1.796719	1.159319	0.052295
C	3.185816	1.198995	0.031141
C	3.184526	-1.201104	0.030012
H	1.200738	-2.067479	0.034346
H	1.202981	2.067506	0.036730
H	3.694276	2.156572	0.013979
H	3.691949	-2.159214	0.011893
H	4.980945	-0.002016	0.004327
Cl	-1.542335	-2.033005	-1.048715
Cl	-1.541350	2.035699	-1.046376
C	-2.469289	-0.007730	1.914973
C	-1.155323	0.003214	2.325691
H	-0.646910	-0.915230	2.611827
H	-0.660677	0.931008	2.605892
H	-3.024765	-0.937683	1.828623
H	-3.038483	0.913428	1.823230

IM3a

B3LYP/BS1 SCF energy: -1410.316383a.u.
M06-L/BS2 SCF energy in solution: -1410.517123 a.u.
M06-L/BS2 Free energy in solution: -1410.064831 a.u.

Pd	-0.342791	0.180514	0.201139
C	0.715041	1.905574	-0.029026
N	1.364014	2.873082	-0.122039
C	2.244928	4.020054	-0.154371
C	2.327946	4.516828	-1.609013
C	3.624345	3.564342	0.356208
C	1.645384	5.100830	0.763945
H	0.652863	5.400976	0.416551
H	2.294685	5.981069	0.761571
H	1.560055	4.733351	1.789963
H	2.725618	3.737112	-2.264085
H	2.991788	5.384641	-1.659931

H	1.341686	4.812439	-1.977502
H	3.547613	3.166211	1.371018
H	4.310637	4.416319	0.361092
H	4.038456	2.784925	-0.288553
C	1.356999	-0.903715	0.464759
N	1.647378	-1.895141	-0.531794
N	2.211203	-0.816593	1.414049
O	1.893509	0.237128	2.310694
C	2.690291	0.080335	3.475750
H	3.760763	0.089522	3.234877
H	2.448755	0.932037	4.117205
H	2.452039	-0.851358	4.003111
C	0.750497	-2.933850	-0.690456
O	0.986282	-3.918774	-1.392433
C	2.936998	-1.882627	-1.328227
C	3.844713	-3.032946	-0.854793
H	4.780905	-3.027936	-1.424815
H	4.084538	-2.909959	0.205230
H	3.351573	-3.994675	-1.000838
C	3.672748	-0.544004	-1.140169
H	3.030512	0.303678	-1.401116
H	4.027129	-0.408132	-0.118825
H	4.534243	-0.536339	-1.815420
C	2.606453	-2.014405	-2.831177
H	3.536301	-1.960817	-3.407490
H	2.110173	-2.956936	-3.052133
H	1.960897	-1.189011	-3.151364
C	-0.553795	-2.865848	0.070906
C	-1.152608	-1.681919	0.532820
C	-1.194385	-4.092659	0.292558
C	-2.361351	-1.839560	1.237833
C	-2.395275	-4.110542	0.992688
H	-0.741020	-5.003390	-0.082998
H	-2.866511	-0.964637	1.643989
H	-2.910638	-5.050984	1.181968
C	-2.195512	0.963360	-0.147911
C	-4.694992	1.551664	-0.636529
C	-4.782712	2.640566	-1.720432
H	-5.833565	2.851974	-1.937859
H	-4.305336	3.565474	-1.384754
H	-4.297284	2.311021	-2.643034
C	-5.332247	0.234608	-1.117710
H	-4.864685	-0.105638	-2.045513
H	-5.218716	-0.550131	-0.365481
H	-6.398646	0.393887	-1.302357
C	-5.336302	2.029026	0.679446
H	-4.857142	2.944481	1.037455
H	-6.397300	2.234764	0.511596
H	-5.249624	1.262576	1.453977
N	-3.294833	1.288966	-0.375750
N	-2.981146	-3.004265	1.471732

TS3a

B3LYP/BS1 SCF energy: -1410.292751a.u.

M06-L/BS2 SCF energy in solution:
-1410.497861 a.u.

M06-L/BS2 Free energy in solution:
-1410.047671 a.u.

Pd	-0.181121	0.346490	0.186852
C	1.258071	1.774291	0.046108
N	2.071057	2.619726	0.013129
C	3.083395	3.649845	-0.001873
C	4.308574	3.104868	-0.759485
C	3.444215	3.980543	1.458480
C	2.500946	4.883387	-0.716409
H	2.219404	4.639292	-1.744399
H	3.249473	5.680840	-0.740870
H	1.615955	5.253119	-0.191317
H	4.704017	2.212509	-0.267158
H	5.093193	3.866913	-0.782701
H	4.045373	2.845746	-1.788612
H	2.567659	4.343397	2.002043
H	4.213227	4.758470	1.477278
H	3.829306	3.095758	1.972152
C	0.879225	-1.415811	0.515077
N	1.151017	-2.155538	-0.695915
N	1.628173	-1.691939	1.536308
O	1.264705	-0.894627	2.638555
C	1.939031	-1.382360	3.791111
H	3.026811	-1.349230	3.655838
H	1.647450	-0.716897	4.607218

H	1.637545	-2.409542	4.027570
C	0.045548	-2.802184	-1.223232
O	0.026792	-3.487542	-2.242155
C	2.472495	-2.161097	-1.421255
C	2.991802	-3.610930	-1.481352
H	3.951246	-3.638389	-2.009732
H	3.144839	-3.999447	-0.470004
H	2.285194	-4.254767	-2.006721
C	3.507818	-1.286020	-0.698421
H	3.163781	-0.253442	-0.599213
H	3.738951	-1.665696	0.296867
H	4.422670	-1.286312	-1.300149
C	2.262720	-1.593000	-2.841314
H	3.217958	-1.589068	-3.377123
H	1.548526	-2.193132	-3.404471
H	1.896354	-0.562264	-2.787050
C	-1.116623	-2.618700	-0.307203
C	-0.968014	-1.595681	0.640851
C	-2.169101	-3.524168	-0.218304
C	-1.862555	-1.618828	1.734144
C	-3.044177	-3.391789	0.859634
H	-2.263906	-4.323581	-0.946480
H	-1.761077	-0.899287	2.542152
H	-3.892458	-4.064583	0.970651
C	-1.950173	1.357881	-0.184995
C	-4.374455	2.247328	-0.607379
C	-4.381840	3.128115	-1.870051
H	-5.399298	3.479817	-2.064105
H	-3.731902	3.998044	-1.740949
H	-4.037863	2.562247	-2.740018
C	-5.263511	1.003593	-0.796823
H	-4.936154	0.421007	-1.662156
H	-5.225948	0.361196	0.086875
H	-6.299483	1.316114	-0.958916
C	-4.814034	3.054176	0.628416
H	-4.164361	3.920953	0.777947
H	-5.839365	3.408179	0.487132
H	-4.780913	2.433798	1.527950
N	-3.020222	1.793950	-0.383954
N	-2.884706	-2.476729	1.831824

IM3b

B3LYP/BS1 SCF energy: -1227.805339 a.u.

M06-L/BS2 SCF energy in solution:
-1227.962115 a.u.

M06-L/BS2 Free energy in solution:
-1227.49623 a.u.

Pd	-0.528885	-0.148128	-0.108154
C	-2.415907	0.420602	-0.229258
N	-3.537098	0.757619	-0.254782
C	-4.934594	1.125541	-0.282389
C	-1.045664	-2.101481	-0.504229
C	0.408672	-2.847996	1.382624
C	1.370155	-1.893730	1.241737
C	0.304506	1.724757	0.325744
C	1.966142	3.668913	0.845369
N	0.968312	2.648980	0.594233
N	1.333450	-0.990657	0.165194
C	-0.577323	-3.083871	0.298857
C	-1.840044	-2.296651	-1.771639
H	-1.977292	-3.348367	-2.046703
H	-2.834802	-1.834598	-1.708805
H	-1.330796	-1.810939	-2.614106
C	-0.979511	-4.542916	0.120614
H	-0.103046	-5.169935	-0.089131
H	-1.438954	-4.940800	1.032958
H	-1.695894	-4.690210	-0.688342
C	0.324684	-3.718346	2.618412
H	-0.695906	-3.731200	3.025092
H	0.591652	-4.764427	2.417146
H	0.983113	-3.366836	3.414756
C	2.426294	-1.627965	2.309228
H	1.966797	-1.173173	3.197339
H	2.942028	-2.535513	2.640438
H	3.181271	-0.933268	1.938463
C	2.416423	-0.803298	-0.664691
C	2.393732	0.185268	-1.686300
C	3.598383	-1.589795	-0.580138
C	3.497461	0.418867	-2.501271
H	1.477450	0.743936	-1.850724
C	4.696990	-1.337907	-1.397622

H	3.634710	-2.418139	0.118366
C	4.674169	-0.323447	-2.357784
H	3.428130	1.181085	-3.275086
H	5.581566	-1.962363	-1.291204
H	5.531720	-0.140460	-2.998012
C	3.077677	3.028453	1.697661
H	2.681499	2.678452	2.654786
H	3.523199	2.180444	1.171861
H	3.856880	3.771091	1.893556
C	1.291615	4.832528	1.590827
H	2.030447	5.614338	1.788472
H	0.483584	5.263105	0.992650
H	0.878439	4.496639	2.546017
C	2.516617	4.121590	-0.520060
H	1.724945	4.564039	-1.131634
H	3.296931	4.872738	-0.366277
H	2.944895	3.272218	-1.058487
C	-5.184382	2.132203	0.855776
H	-4.574714	3.029967	0.721464
H	-6.238238	2.425366	0.859491
H	-4.943221	1.688779	1.825604
C	-5.231923	1.760996	-1.653252
H	-5.026542	1.054698	-2.462044
H	-6.286387	2.047924	-1.701053
H	-4.621083	2.654608	-1.808086
C	-5.765675	-0.154581	-0.076375
H	-5.567199	-0.878871	-0.870846
H	-5.529545	-0.620021	0.884207
H	-6.830543	0.095598	-0.090599

TS3b

B3LYP/BS1 SCF energy: -1227.785839a.u.

M06-L/BS2 SCF energy in solution: -1227.939163a.u.

M06-L/BS2 Free energy in solution: -1227.475874 a.u.

Pd	-0.345553	-0.077252	-0.069806
C	-2.330039	-0.203666	-0.356163
N	-3.495537	-0.293684	-0.479052
C	-4.924014	-0.447861	-0.610693
C	0.103515	-2.147003	-0.202817
C	1.109614	-2.346629	1.960156
C	1.891731	-1.305181	1.521081
C	-0.073434	1.987254	0.210220
C	0.380211	4.521999	0.679432
N	0.105023	3.128998	0.415632
N	1.611736	-0.855862	0.223133
C	0.278832	-2.913991	0.921064
C	-0.073079	-2.638356	-1.610367
H	0.151963	-3.708198	-1.709397
H	-1.083133	-2.447395	-1.996897
H	0.618836	-2.101346	-2.271538
C	-0.219996	-4.340042	1.020731
H	0.594716	-5.057479	1.193420
H	-0.925875	-4.461131	1.853795
H	-0.744717	-4.648427	0.112671
C	1.132102	-2.917887	3.353203
H	0.175708	-2.759169	3.871262
H	1.302128	-4.001643	3.341715
H	1.913733	-2.473291	3.973622
C	2.859850	-0.494094	2.345366
H	2.378367	0.416659	2.732963
H	3.241626	-1.054712	3.202531
H	3.717540	-0.169601	1.749662
C	2.613363	-0.669039	-0.728628
C	2.390411	0.141255	-1.862900
C	3.859050	-1.333397	-0.631038
C	3.380742	0.309561	-2.829721
H	1.427154	0.633084	-1.968359
C	4.846752	-1.141390	-1.592346
H	4.027588	-2.008306	0.201800
C	4.621504	-0.317814	-2.699998
H	3.178329	0.940099	-3.692311
H	5.797765	-1.657667	-1.485066
H	5.391947	-0.181627	-3.453183
C	1.672654	4.601519	1.513499
H	1.555784	4.066959	2.460052
H	2.512533	4.162272	0.968897
H	1.905543	5.648278	1.730711
C	-0.812610	5.106154	1.458845
H	-0.627909	6.162607	1.675056

H	-1.733861	5.026752	0.875053
H	-0.953775	4.577071	2.405238
C	0.554962	5.237074	-0.673245
H	-0.354267	5.155950	-1.275270
H	0.766632	6.296815	-0.502824
H	1.384894	4.802001	-1.236306
C	-5.603737	0.518779	0.377282
H	-5.330889	1.554368	0.155983
H	-6.690667	0.421178	0.299161
H	-5.307070	0.293372	1.405241
C	-5.309907	-0.106675	-2.062313
H	-4.805160	-0.775864	-2.764331
H	-6.390709	-0.217441	-2.191212
H	-5.033918	0.923116	-2.305278
C	-5.280899	-1.909157	-0.278996
H	-4.779943	-2.595346	-0.967071
H	-4.977589	-2.159497	0.741079
H	-6.361899	-2.053485	-0.367696

Ti(OMe)$_2$-C$_6$H$_6$

B3LYP/BS1 SCF energy: -1312.027499a.u.
M06-L/BS2 SCF energy in solution:
-1312.107474 a.u.
M06-L/BS2 Free energy in solution:
-1311.965672 a.u.

Ti	-0.185917	0.047209	0.000796
O	-1.220072	1.523628	0.002532
O	-1.338059	-1.323211	-0.000506
C	1.820216	1.184914	0.688812
C	1.461942	-0.024724	1.426019
C	1.753229	-1.251770	0.683495
C	1.752221	-1.249371	-0.689040
C	1.459820	-0.019713	-1.426836
C	1.819255	1.187323	-0.685887
H	1.821844	-2.190566	1.224375
H	1.527015	-0.019236	-2.507749
H	1.932548	2.122528	-1.225161
H	1.934269	2.118254	1.231165
H	1.530587	-0.028052	2.506842
H	1.819961	-2.186294	-1.233282
C	-2.508568	2.065876	-0.001163
H	-2.665231	2.696186	0.885418
H	-2.661397	2.693307	-0.890456
H	-3.275399	1.277479	-0.001518
C	-2.427049	-2.193530	0.000390
H	-3.057949	-2.035305	-0.885741
H	-2.088771	-3.239312	-0.007070
H	-3.048637	-2.044950	0.894725

Cr-Pyr

B3LYP/BS1 SCF energy: -1791.208705 a.u.
M06-L/BS2 SCF energy in solution:
-1791.379784 a.u.
M06-L/BS2 Free energy in solution:
-1791.176923 a.u.

Cr	0.540415	0.350115	-0.001925
O	0.334642	3.331515	-0.015164
O	2.583478	0.165872	-2.184312
O	2.586823	0.186894	2.179029
N	-0.330051	-1.609403	0.006464
C	0.488693	2.173256	-0.010101
C	1.813067	0.283544	-1.314516
C	1.815035	0.295985	1.309342
C	-0.796774	-0.938364	-1.149662
C	-1.579681	0.147867	-0.719436
C	-1.578067	0.153275	0.722072
C	-0.794017	-0.929638	1.158567
C	0.306195	-2.923755	0.010753
H	0.938586	-3.017782	0.892780
H	0.936061	-3.024807	-0.872300
H	-0.447170	-3.720531	0.014960
C	-0.619176	-1.511447	-2.520233
H	-0.937931	-0.780221	-3.264807
H	-1.227021	-2.414816	-2.656410
H	0.423526	-1.757358	-2.736637
C	-2.341576	1.100759	-1.594135
H	-1.957349	1.099722	-2.616649
H	-2.264817	2.124797	-1.220102
H	-3.405376	0.834933	-1.632630
C	-2.337854	1.112870	1.591281

H	-1.952466	1.118237	2.613346
H	-3.401899	0.848503	1.632800
H	-2.260446	2.134308	1.210314
C	-0.612980	-1.492476	2.532935
H	-0.930507	-0.756035	3.272876
H	0.430385	-1.736168	2.748677
H	-1.219917	-2.395213	2.677091

Fe-Pyr^{+}

B3LYP/BS1 SCF energy: -1863.739515a.u.
M06-L/BS2 SCF energy in solution:
-1863.912209 a.u.
M06-L/BS2 Free energy in solution:
-1863.645639 a.u.

Fe	0.532847	0.018311	-0.009958
C	-1.139201	-0.994937	0.743698
C	-1.076454	0.357012	1.167915
N	-1.076348	1.159512	-0.000459
C	-1.111487	0.329015	-1.148683
C	-1.161193	-1.012206	-0.690024
C	-1.223096	-2.190810	1.645704
C	-1.197605	0.939028	2.539635
C	-1.247178	2.617350	-0.014986
C	-1.281695	0.876455	-2.529508
C	-1.272712	-2.229795	-1.559360
C	2.217247	-0.286038	1.157527
C	2.235124	1.020412	0.592917
C	2.214772	0.887291	-0.830471
C	2.185636	-0.502918	-1.139245
C	2.180044	-1.228818	0.087774
H	-0.747251	-2.007064	2.611554
H	-0.742052	-3.063585	1.198804
H	-2.270975	-2.450570	1.833889
H	-0.497166	1.760405	2.710658
H	-0.992572	0.170473	3.286484
H	-2.213590	1.310520	2.717243
H	-0.764611	3.050025	0.859839
H	-2.313010	2.862326	-0.004429
H	-0.786762	3.030584	-0.910934
H	-0.604016	1.707485	-2.739865
H	-2.309845	1.223159	-2.686393
H	-1.081541	0.095122	-3.264379
H	-0.847254	-2.062330	-2.551225
H	-2.325016	-2.506374	-1.690049
H	-0.758920	-3.086378	-1.117382
H	2.211263	-0.522429	2.213086
H	2.261022	1.950533	1.145442
H	2.230188	1.697873	-1.546634
H	2.152577	-0.933734	-2.130930
H	2.146749	-2.304945	0.190002

Ti-Pyr

B3LYP/BS1 SCF energy: -2982.626279 a.u.
M06-L/BS2 SCF energy in solution:
-2982.884483 a.u.
M06-L/BS2 Free energy in solution:
-2982.250563 a.u.

C	-1.062453	5.633235	1.170255
H	-0.647897	5.510784	2.176751
H	-2.099035	5.280916	1.192588
H	-1.075981	6.706061	0.943857
Ti	-0.042676	1.316788	0.584986
Si	-0.041810	4.676160	-0.103645
N	-1.737343	0.838930	1.644987
N	-0.028952	-1.128062	0.094788
N	1.643554	0.870164	1.663193
N	-0.067053	2.972199	0.286634
C	-2.191414	1.516044	2.758947
H	-1.599508	2.307075	3.198760
C	-3.415927	1.018404	3.152646
H	-4.002377	1.347707	4.000199
C	-3.755626	-0.009818	2.230956
H	-4.686835	-0.558953	2.202593
C	-2.705525	-0.114727	1.331216
C	1.884231	1.298816	2.952775
H	1.330451	2.129940	3.368837
C	2.858001	0.514196	3.536939
H	3.243846	0.609791	4.543207
C	3.257381	-0.442510	2.561602
H	4.007459	-1.207921	2.692081

C	2.501586	-0.198716	1.424811
C	1.108426	-0.596831	-0.527079
C	0.659223	0.257787	-1.535174
H	1.295446	0.827640	-2.192885
C	-0.763072	0.242592	-1.529165
H	-1.419813	0.798967	-2.178692
C	-1.180149	-0.616264	-0.513744
C	-0.017460	-2.022740	1.267802
H	-0.119300	-3.058562	0.944370
H	-0.843830	-1.752351	1.921456
H	0.922509	-1.882358	1.797740
C	1.741246	5.310393	-0.087179
H	2.185387	5.202023	0.908067
H	1.791590	6.370302	-0.363936
H	2.364106	4.748108	-0.791116
C	-2.596945	-0.883768	0.008428
C	-3.624771	-0.266451	-1.001159
C	-3.833500	1.124276	-0.989988
H	-3.308094	1.738587	-0.267058
C	-4.718509	1.726738	-1.882207
H	-4.857369	2.803755	-1.850307
C	-5.435069	0.952242	-2.797425
H	-6.132814	1.419754	-3.486226
C	-5.253134	-0.428374	-2.805188
H	-5.812788	-1.049518	-3.499072
C	-4.355448	-1.031718	-1.918681
H	-4.246429	-2.108490	-1.943786
C	-2.788518	-2.420017	0.108815
C	-3.412522	-3.042022	1.195611
H	-3.747137	-2.446720	2.034725
C	-3.573288	-4.429684	1.233500
H	-4.058027	-4.884906	2.092552
C	-3.109217	-5.227630	0.188957
H	-3.231862	-6.306179	0.223246
C	-2.478297	-4.622961	-0.899765
H	-2.106387	-5.227438	-1.722386
C	-2.322507	-3.237191	-0.936594
H	-1.832459	-2.784745	-1.794742
C	2.537575	-0.770506	0.001903
C	3.025634	-2.236302	-0.056258
C	2.271500	-3.271591	-0.621897
H	1.285310	-3.072604	-1.028470
C	2.772243	-4.575619	-0.691112
H	2.162622	-5.358676	-1.133097
C	4.043383	-4.867375	-0.203114
H	4.432910	-5.879846	-0.253754
C	4.819447	-3.837593	0.334731
H	5.822644	-4.043163	0.697155
C	4.321264	-2.538398	0.395381
H	4.953005	-1.742911	0.774156
C	3.483963	0.063921	-0.917094
C	3.759051	-0.378946	-2.221012
H	3.326766	-1.310497	-2.574420
C	4.590444	0.354644	-3.066050
H	4.790095	-0.007202	-4.070863
C	5.172584	1.543059	-2.618160
H	5.825554	2.113339	-3.272649
C	4.913170	1.985767	-1.321987
H	5.364363	2.904837	-0.958968
C	4.073502	1.254042	-0.476473
H	3.880659	1.609608	0.528716
C	-0.782626	4.934810	-1.828461
H	-0.202104	4.405248	-2.591987
H	-1.812365	4.564131	-1.874306
H	-0.796811	5.996995	-2.100077

IM3Ti-F

B3LYP/BS1 SCF energy: -2856.406803 a.u.
M06-L/BS2 SCF energy in solution:
-2856.650992 a.u.
M06-L/BS2 Free energy in solution:
-2856.483548 a.u.

Ti	0.385742	-0.312165	0.046028
N	2.659112	-0.803388	0.050326
C	5.424122	-1.206171	0.187441
C	3.485227	0.172784	0.479349
C	3.207912	-1.979125	-0.311167
C	4.577589	-2.219827	-0.255170
C	4.863196	0.014293	0.561441
H	3.014133	1.104885	0.768691
H	2.519276	-2.740036	-0.658097

H	4.962323	-3.187768	-0.557267
H	5.476816	0.836616	0.912385
H	6.497374	-1.362347	0.239339
C	-2.679946	-0.585681	-0.004926
C	-4.833173	-2.353638	-0.182654
C	-3.647685	-0.408809	-1.000579
C	-2.789368	-1.643628	0.902571
C	-3.866746	-2.524666	0.809561
C	-4.720981	-1.293354	-1.085439
H	-3.546958	0.409858	-1.706537
H	-2.042634	-1.758499	1.681783
H	-3.950032	-3.343769	1.517597
H	-5.468091	-1.156231	-1.861599
H	-5.669864	-3.042149	-0.253129
Cl	0.590000	-0.076480	2.305451
Cl	0.074162	-2.152700	-1.219386
C	-0.926970	2.640612	0.083076
N	-1.551106	0.302077	0.076418
C	-1.800930	1.589861	0.327005
C	0.884180	1.332090	-0.958395
C	0.289428	2.533065	-0.627250
F	-2.983117	1.926696	0.858614
F	-1.299888	3.867627	0.488105
F	0.826680	3.698007	-1.027878
F	1.887368	1.408277	-1.873873

TS3Ti-F

B3LYP/BS1 SCF energy: -2856.341774 a.u.
M06-L/BS2 SCF energy in solution:
-2856.584209 a.u.
M06-L/BS2 Free energy in solution:
-2856.41695 a.u.

Ti	0.466655	0.124850	0.816640
N	2.487988	-0.602444	0.052326
C	4.864227	-1.594421	-1.028593
C	3.521458	0.220164	-0.211784
C	2.631049	-1.915721	-0.217070
C	3.798800	-2.450076	-0.748878
C	4.718689	-0.235953	-0.756548
H	3.375054	1.262280	0.047021

H	1.781013	-2.547116	0.016126
H	3.863552	-3.516151	-0.936894
H	5.518465	0.469725	-0.952813
H	5.788710	-1.980150	-1.447543
C	-2.650586	-0.445268	-0.026759
C	-5.089341	-1.715298	-0.495161
C	-3.774931	0.315731	-0.381855
C	-2.745263	-1.837803	0.097591
C	-3.965023	-2.465752	-0.143953
C	-4.990358	-0.326167	-0.606354
H	-3.700013	1.394533	-0.452542
H	-1.872359	-2.406068	0.396834
H	-4.038074	-3.544816	-0.047954
H	-5.863240	0.263201	-0.870529
H	-6.039534	-2.208895	-0.674836
Cl	1.660586	1.799788	1.929689
Cl	0.223052	-1.614900	2.245875
C	-0.495155	2.146580	-0.713728
N	-1.389019	0.170244	0.168126
C	-1.157262	1.538947	0.398232
C	-0.057606	-0.124148	-1.173499
C	0.012259	1.238857	-1.625875
F	-2.033755	2.254331	1.140673
F	-0.225450	3.442641	-0.756238
F	0.679620	1.596856	-2.732268
F	-0.256012	-1.088157	-2.070534

IM4Ti-F

B3LYP/BS1 SCF energy: -2856.358576 a.u.
M06-L/BS2 SCF energy in solution:
-2856.599551 a.u.
M06-L/BS2 Free energy in solution:
-2856.43131 a.u.

Ti	0.495441	-0.081228	0.932612
N	2.427219	-0.575736	-0.025723
C	4.806685	-1.251010	-1.314036
C	3.369017	0.358282	-0.265164
C	2.663448	-1.845027	-0.416718
C	3.839839	-2.222105	-1.053253
C	4.562576	0.061621	-0.915201

H	3.153997	1.356771	0.096765
H	1.882910	-2.565799	-0.199634
H	3.985659	-3.257965	-1.338759
H	5.285462	0.850656	-1.090421
H	5.733013	-1.513794	-1.815620
C	-2.652560	-0.292638	-0.220061
C	-5.247580	-1.215957	-0.602977
C	-3.672755	0.620577	-0.502747
C	-2.916650	-1.659812	-0.120809
C	-4.218853	-2.116570	-0.321401
C	-4.972399	0.150196	-0.687531
H	-3.457872	1.680740	-0.561110
H	-2.114693	-2.345192	0.125103
H	-4.428540	-3.179179	-0.246575
H	-5.769473	0.856116	-0.900220
H	-6.261343	-1.576117	-0.749740
Cl	1.664245	1.269855	2.412949
Cl	0.032252	-2.025972	2.013874
C	-0.269863	2.201136	-0.607131
N	-1.292875	0.168956	-0.053052
C	-0.907126	1.467027	0.496885
C	-0.248396	-0.048163	-1.073788
C	0.114220	1.299280	-1.546349
F	-0.527346	-1.019371	-1.975543
F	0.980126	1.495303	-2.540480
F	0.131207	3.461959	-0.495769
F	-1.827019	2.077800	1.274000

IM3Ti-SiH$_3$
B3LYP/BS1 SCF energy: -3622.263172 a.u.
M06-L/BS2 SCF energy in solution:
-3622.445920 a.u.
M06-L/BS2 Free energy in solution:
-3622.193183 a.u.

Ti	-0.410306	-0.628068	-0.161116
N	-2.741673	-1.075864	-0.385420
C	-5.469353	-1.424607	-0.897638
C	-3.527344	-0.031302	-0.715360
C	-3.310853	-2.292928	-0.308777
C	-4.664173	-2.508856	-0.556911
C	-4.886611	-0.160070	-0.976736
H	-3.038723	0.935027	-0.772639
H	-2.650430	-3.108407	-0.037804
H	-5.068068	-3.512689	-0.481989
H	-5.468812	0.717127	-1.237049
H	-6.528072	-1.560411	-1.097152
C	2.598662	-1.056227	0.157377
C	4.672308	-2.904933	0.449297
C	3.154059	-1.289610	1.420731
C	3.069849	-1.758104	-0.958059
C	4.110812	-2.676522	-0.807646
C	4.189029	-2.212168	1.562299
H	2.767705	-0.747139	2.277732
H	2.618145	-1.581269	-1.929175
H	4.476980	-3.216656	-1.675730
H	4.617987	-2.390626	2.543995
H	5.478842	-3.623070	0.563237
Cl	-0.552141	-0.071114	-2.389191
Cl	-0.184077	-2.747194	0.617517
C	0.809368	2.189598	-0.118398
N	1.525751	-0.110763	0.012305
C	1.804680	1.183585	-0.255305
H	3.956814	1.157584	-2.081176
H	4.562006	1.140537	0.267792
H	3.698540	3.143912	-0.728777
H	1.677580	3.583149	-2.354582
H	1.740109	4.894800	-0.332174
H	-0.366956	4.332057	-1.346275
C	-0.807676	0.741341	1.162153
C	-0.355385	1.986776	0.712108
H	-2.000937	-1.134447	2.767010
H	-3.223528	0.920006	2.592677
H	-1.213113	0.827788	3.921097
H	-1.669598	3.247187	2.779843
H	-2.546758	3.744930	0.594023
H	-0.478662	4.753922	1.332193
Si	3.604254	1.664814	-0.732276
Si	1.000940	3.825463	-1.055715
Si	-1.297010	3.515314	1.368431
Si	-1.851854	0.336978	2.669197

TS3Ti-SiH$_3$

B3LYP/BS1 SCF energy: -3622.237911 a.u.

M06-L/BS2 SCF energy in solution:

-3622.427618 a.u.

M06-L/BS2 Free energy in solution:

-3622.17319 a.u.

Ti	-0.422628	-0.030895	-0.830531
N	-2.477316	-0.911132	-0.401288
C	-5.001039	-1.926588	0.238630
C	-3.504498	-0.084919	-0.120998
C	-2.702638	-2.239135	-0.373285
C	-3.943678	-2.784164	-0.061054
C	-4.773872	-0.552275	0.207703
H	-3.294849	0.975268	-0.190510
H	-1.859873	-2.870976	-0.628595
H	-4.069498	-3.861328	-0.059412
H	-5.564386	0.158058	0.423487
H	-5.982144	-2.321205	0.485046
C	2.605557	-0.831199	0.125177
C	4.951829	-2.291473	0.566906
C	3.607180	-0.322464	0.970157
C	2.774670	-2.083287	-0.481795
C	3.947389	-2.802902	-0.257957
C	4.777691	-1.048981	1.180255
H	3.452912	0.630670	1.465532
H	2.002018	-2.465635	-1.136382
H	4.077760	-3.767834	-0.738881
H	5.547909	-0.647544	1.832078
H	5.862357	-2.859000	0.734138
Cl	-1.536658	1.575511	-2.171280
Cl	-0.109771	-1.720644	-2.365567
C	0.354548	1.971182	0.393745
N	1.416707	-0.073434	-0.069068
C	1.440740	1.322105	-0.247787
H	2.228293	3.305753	-1.960293
H	3.364397	1.170704	-2.230026
H	3.920315	2.638349	-0.391278
H	0.910334	4.617472	0.951072
H	-1.417419	4.036720	0.726386
H	-0.012932	4.238021	-1.243999
C	-0.112513	-0.250414	1.162846
C	-0.366876	1.131773	1.315313
H	0.619017	-2.815995	1.699246
H	-1.498711	-2.123439	2.621306
H	0.561362	-1.375398	3.643808
H	-0.888783	2.993087	3.350042
H	-1.535271	0.693475	3.731223
H	-2.835011	2.085714	2.265702
Si	2.815237	2.141782	-1.256140
Si	-0.063390	3.806503	0.166356
Si	-1.451381	1.772875	2.712486
Si	-0.100707	-1.695415	2.351138

IM4Ti-SiH$_3$

B3LYP/BS1 SCF energy: -3622.268319 a.u.

M06-L/BS2 SCF energy in solution:

-3622.458295 a.u.

M06-L/BS2 Free energy in solution:

-3622.20253 a.u.

Ti	0.369322	0.374974	0.793172
N	2.534513	-0.188002	0.738463
C	5.243950	-0.873449	0.731642
C	3.489047	0.758764	0.631682
C	2.923186	-1.473914	0.857908
C	4.262691	-1.855020	0.847647
C	4.846691	0.460191	0.628060
H	3.138327	1.782100	0.560933
H	2.128321	-2.198543	0.989664
H	4.519994	-2.904477	0.941030
H	5.571445	1.262656	0.545451
H	6.296727	-1.139353	0.727640
C	-2.824449	-0.253315	-0.004434
C	-5.539469	-0.559226	0.525011
C	-3.726191	-0.391844	-1.065725
C	-3.268863	-0.264818	1.318470
C	-4.632583	-0.415825	1.576448
C	-5.085470	-0.548909	-0.796078
H	-3.360041	-0.369908	-2.087867
H	-2.550343	-0.185709	2.126466
H	-4.981987	-0.428323	2.604229

H	-5.787916	-0.656972	-1.616990
H	-6.598417	-0.679308	0.733481
Cl	0.707269	2.169811	2.176866
Cl	-0.032348	-1.419122	2.329062
C	0.357960	0.981603	-1.392607
N	-1.420870	-0.076032	-0.304164
C	-0.853593	1.244528	-0.606317
H	-0.836603	3.935277	-0.619865
H	-2.449256	2.943284	0.912453
H	-2.878502	2.958501	-1.459572
H	0.674875	2.977200	-3.322814
H	2.588100	1.596337	-2.828139
H	1.894410	3.307650	-1.280061
C	-0.644141	-1.086126	-0.985203
C	0.407224	-0.447006	-1.635406
H	-2.142231	-3.100601	0.170307
H	0.152650	-3.678788	-0.400952
H	-1.498588	-3.449501	-2.132322
H	1.507794	-0.894681	-4.116159
H	1.432097	-2.799906	-2.630809
H	3.066759	-1.055129	-2.280677
Si	-1.816325	2.841367	-0.421005
Si	1.407857	2.275974	-2.230970
Si	1.676489	-1.339011	-2.709048
Si	-1.055008	-2.923939	-0.811312

IM3Pd-F

B3LYP/BS1 SCF energy: -1467.471842 a.u.

M06-L/BS2 SCF energy in solution: -1467.714253 a.u.

M06-L/BS2 Free energy in solution: -1467.385212 a.u.

Pd	-0.485119	-0.193107	0.034298
C	-2.060267	0.945335	0.080416
N	-3.060999	1.546941	0.106941
C	-4.457541	1.935467	0.183246
C	-1.779649	-1.713323	0.066349
C	-0.172438	-3.545452	-0.415938
C	0.985664	-2.836358	-0.310494
C	0.785827	1.414154	-0.148861
C	2.387221	3.432816	-0.589225
N	1.461581	2.344659	-0.352408
C	-1.476638	-3.012783	-0.178224
N	1.111390	-1.521803	-0.013583
C	2.405400	-1.055059	0.357396
C	2.604475	-0.496958	1.630747
C	3.494439	-1.105800	-0.531131
C	3.852130	0.008333	2.002247
H	1.768957	-0.476419	2.323466
C	4.740786	-0.608460	-0.152907
H	3.352230	-1.538231	-1.515288
C	4.928092	-0.044696	1.113710
H	3.985504	0.427430	2.996259
H	5.571648	-0.663943	-0.851465
H	5.902772	0.335153	1.406494
C	-4.974830	1.496277	1.565989
H	-4.845943	0.418626	1.692411
H	-4.434825	2.010753	2.365815
H	-6.037871	1.739933	1.651180
C	-5.192716	1.183111	-0.942192
H	-6.262858	1.403913	-0.889261
H	-4.821451	1.491865	-1.923421
H	-5.042980	0.106236	-0.834555
C	-4.555553	3.457980	0.003097
H	-4.154819	3.763540	-0.967491
H	-5.604634	3.762344	0.054688
H	-4.005261	3.981752	0.789928
C	1.576308	4.644969	-1.080888
H	0.842508	4.954235	-0.331271
H	2.254540	5.482545	-1.266880
H	1.050529	4.411551	-2.010826
C	3.100370	3.742656	0.739889
H	3.813220	4.557967	0.585714
H	2.381271	4.050901	1.504149
H	3.640504	2.863726	1.100087
C	3.391294	2.964586	-1.658815
H	3.929630	2.076480	-1.319056
H	2.878580	2.726709	-2.594946
H	4.112606	3.764288	-1.851198
F	-3.114797	-1.479783	0.342292
F	-2.469582	-3.933871	-0.197089

F	-0.105560	-4.851069	-0.784997
F	2.130337	-3.535031	-0.503024

TS3Pd-F
B3LYP/BS1 SCF energy: -1467.418717 a.u.
M06-L/BS2 SCF energy in solution:
-1467.658119 a.u.
M06-L/BS2 Free energy in solution:
-1467.33553 a.u.

Pd	-0.389870	0.077934	0.017736
C	-2.376167	0.284071	-0.318322
N	-3.539162	0.310626	-0.462435
C	-4.973789	0.244770	-0.627097
C	-0.324867	-1.974811	0.078187
C	0.848444	-2.472399	2.047599
C	1.758034	-1.663102	1.432811
C	0.334332	2.019282	0.259516
C	1.462363	4.345260	0.663070
N	0.812162	3.074926	0.431377
N	1.389851	-1.174456	0.202056
C	-0.259350	-2.757117	1.217791
C	2.299461	-1.177946	-0.883906
C	1.977976	-0.499852	-2.070395
C	3.518610	-1.877343	-0.810512
C	2.857846	-0.510914	-3.152095
H	1.028745	0.026046	-2.135158
C	4.399006	-1.862479	-1.889079
H	3.777505	-2.413002	0.095574
C	4.076909	-1.184529	-3.067809
H	2.585452	0.013034	-4.064309
H	5.341860	-2.396780	-1.809359
H	4.763930	-1.187455	-3.908747
C	1.926081	4.894884	-0.698601
H	2.627874	4.205813	-1.175878
H	1.074611	5.044612	-1.368110
H	2.426899	5.856236	-0.551813
C	2.663367	4.096153	1.594702
H	3.178744	5.041832	1.786634
H	2.333731	3.678554	2.549763
H	3.370505	3.399064	1.137562
C	0.440708	5.291162	1.320340
H	0.091167	4.883921	2.272907
H	0.909952	6.261195	1.508425
H	-0.423553	5.443810	0.668070
C	-5.612284	1.259281	0.339220
H	-5.274202	2.275125	0.116484
H	-6.700931	1.225339	0.237833
H	-5.352432	1.024611	1.375000
C	-5.300158	0.597099	-2.090061
H	-4.821313	-0.108421	-2.774254
H	-6.382273	0.551582	-2.243700
H	-4.956379	1.606270	-2.333199
C	-5.420621	-1.190995	-0.293307
H	-4.943396	-1.910799	-0.963457
H	-5.157469	-1.449756	0.735724
H	-6.505646	-1.272547	-0.407492
F	-0.714889	-2.551992	-1.084474
F	-1.040661	-3.855568	1.378487
F	0.957602	-2.881905	3.324140
F	2.856311	-1.157622	2.040329

IM3Pd-SiH$_3$
B3LYP/BS1 SCF energy: -2233.316459 a.u.
M06-L/BS2 SCF energy in solution:
-2233.497904 a.u.
M06-L/BS2 Free energy in solution:
-2233.08653 a.u.

Pd	-0.479628	0.269583	-0.231538
C	-2.329522	0.983961	-0.135897
N	-3.422795	1.388112	-0.044121
C	-4.808102	1.799675	0.038505
C	-1.172733	-1.633536	-0.649160
C	0.306285	-2.540337	1.166702
C	1.363924	-1.689277	0.881212
C	0.501055	2.049987	0.263844
C	2.147923	3.952544	0.969648
N	1.177003	2.940074	0.604679
N	1.295609	-0.798030	-0.180885
C	-0.783012	-2.649809	0.163436
H	-2.090723	-2.935821	-3.012925

H	-3.564762	-1.294481	-2.049907
H	-1.563210	-0.606974	-3.154922
H	-0.718482	-5.417950	-0.483717
H	-2.227482	-4.757228	1.264890
H	-2.756864	-4.231413	-1.000916
H	-0.819603	-3.300216	3.630427
H	0.181627	-5.064527	2.361814
H	1.569828	-3.455477	3.449901
H	2.400240	-1.058513	3.397956
H	3.774388	-2.608353	2.180732
H	3.669498	-0.287771	1.530860
C	2.356010	-0.627624	-1.057141
C	2.410612	0.477846	-1.940622
C	3.408969	-1.572232	-1.142960
C	3.496001	0.667288	-2.790026
H	1.578969	1.174035	-1.964953
C	4.497421	-1.362777	-1.988135
H	3.345116	-2.492597	-0.571400
C	4.562294	-0.238338	-2.811844
H	3.501861	1.527142	-3.456001
H	5.290676	-2.105568	-2.018314
H	5.404258	-0.087512	-3.480426
C	3.000432	4.245045	-0.279404
H	3.480423	3.332920	-0.643226
H	2.383402	4.658091	-1.082471
H	3.774264	4.975522	-0.026230
C	3.012551	3.379439	2.107668
H	3.764161	4.119493	2.397361
H	2.399703	3.145445	2.982430
H	3.522929	2.468061	1.786228
C	1.384258	5.206270	1.429348
H	0.759910	4.986226	2.299747
H	2.100121	5.985831	1.704704
H	0.746124	5.590099	0.628464
C	-4.876212	3.074079	0.898858
H	-4.290665	3.880313	0.448289
H	-5.915994	3.403733	0.978408
H	-4.494394	2.884796	1.905824
C	-5.303350	2.071480	-1.394201
H	-5.220270	1.171695	-2.009032
H	-6.352714	2.378662	-1.362827
H	-4.721409	2.869826	-1.862930
C	-5.600857	0.649908	0.687347
H	-5.525285	-0.259629	0.086066
H	-5.224968	0.436030	1.691479
H	-6.654688	0.933292	0.762816
Si	-2.126078	-1.652643	-2.257407
Si	-1.634804	-4.333236	-0.033894
Si	0.300378	-3.618387	2.697308
Si	2.872254	-1.433635	2.035549

TS3Pd-SiH$_3$

B3LYP/BS1 SCF energy: -2233.304870 a.u.

M06-L/BS2 SCF energy in solution:

-2233.484726 a.u.

M06-L/BS2 Free energy in solution:

-2233.07512 a.u.

Pd	0.456913	0.283419	0.278014
C	2.452132	0.557795	0.317049
N	3.615953	0.698094	0.323535
C	5.057393	0.801548	0.341330
C	0.407046	-1.812204	0.493031
C	-0.735910	-2.204800	-1.588222
C	-1.668441	-1.325661	-1.036487
C	-0.200980	2.244826	-0.102931
C	-1.249901	4.479528	-0.972680
N	-0.641545	3.262121	-0.482481
N	-1.345950	-0.814062	0.216359
C	0.283041	-2.601763	-0.635439
H	0.637052	-3.622056	2.652976
H	2.370867	-1.955311	2.457222
H	0.168386	-1.321650	3.169985
H	0.486333	-5.408216	-1.012590
H	2.303477	-4.099230	-1.861890
H	2.044437	-4.369375	0.499587
H	-0.255507	-1.955838	-4.346985
H	-0.090870	-4.155798	-3.410662
H	-2.246357	-3.145653	-3.742444
H	-2.880766	-0.430386	-3.345733
H	-4.367434	-1.644623	-1.920891
H	-3.665106	0.577192	-1.311016

C	-2.333238	-0.646679	1.208592
C	-2.221039	0.360479	2.184859
C	-3.438147	-1.520285	1.272797
C	-3.203099	0.514967	3.160918
H	-1.354629	1.013886	2.166493
C	-4.424083	-1.347059	2.243077
H	-3.500907	-2.346457	0.572392
C	-4.316655	-0.328417	3.191896
H	-3.095549	1.300384	3.904484
H	-5.270676	-2.027839	2.267806
H	-5.079922	-0.203979	3.954254
C	-2.141921	5.047183	0.146890
H	-2.923900	4.333355	0.418563
H	-1.550664	5.273630	1.038428
H	-2.616765	5.970023	-0.198792
C	-2.087073	4.119777	-2.214357
H	-2.566197	5.022578	-2.604457
H	-1.455622	3.693792	-2.998715
H	-2.863084	3.392665	-1.961247
C	-0.123147	5.464916	-1.332607
H	0.524445	5.047912	-2.108716
H	-0.558267	6.396082	-1.706782
H	0.487428	5.695217	-0.455083
C	5.490391	1.633121	-0.880168
H	5.056933	2.636378	-0.840909
H	6.580313	1.725676	-0.891509
H	5.172785	1.152276	-1.809306
C	5.467120	1.497144	1.652845
H	5.134856	0.918355	2.518727
H	6.556454	1.589031	1.692634
H	5.031500	2.498227	1.714881
C	5.636512	-0.623733	0.270353
H	5.300403	-1.221209	1.121780
H	5.322877	-1.124177	-0.649602
H	6.729348	-0.575104	0.286417
Si	0.915941	-2.214441	2.247088
Si	1.290671	-4.176437	-0.769131
Si	-0.841638	-2.874913	-3.328247
Si	-3.213751	-0.702367	-1.919303

IM3Ti-HMe

B3LYP/BS1 SCF energy: -2617.961094 a.u.
M06-L/BS2 SCF energy in solution:
-2618.120392 a.u.
M06-L/BS2 Free energy in solution:
-2617.78707 a.u.

Ti	0.524939	-0.222080	1.135807
N	-1.076771	-1.529892	-0.186935
C	-2.898617	-2.831058	-1.875990
C	-2.192452	-2.102647	0.300729
C	-0.870960	-1.602477	-1.516315
C	-1.745662	-2.238502	-2.390342
C	-3.122367	-2.758673	-0.504260
H	-2.328579	-2.027333	1.373086
H	0.042164	-1.143011	-1.875280
H	-1.520176	-2.266189	-3.451237
H	-4.002409	-3.201115	-0.049879
H	-3.603059	-3.336889	-2.529712
C	-1.056062	1.680188	-0.223603
C	-3.717423	2.620955	-0.270710
C	-1.737216	1.857307	-1.445643
C	-1.729345	2.008498	0.969513
C	-3.050529	2.455348	0.943161
C	-3.047896	2.327950	-1.462295
H	-1.241193	1.622751	-2.381529
H	-1.200521	1.936719	1.915681
H	-3.547725	2.696533	1.878183
H	-3.552367	2.462169	-2.415465
H	-4.739939	2.985373	-0.291527
Cl	2.072214	0.758519	2.504938
Cl	-0.729074	-1.026081	2.918248
C	2.625078	0.817498	-0.940055
N	0.249847	1.145961	-0.141262
C	1.251429	1.533242	-1.162644
C	1.407872	3.064450	-1.163952
H	1.913603	3.392870	-0.250134
H	0.430132	3.549455	-1.203600
H	1.980628	3.409647	-2.027569
C	3.655390	1.326582	-1.966635
H	3.313636	1.199818	-3.000835
H	4.606253	0.799825	-1.861891

H	3.864092	2.386738	-1.814018
C	1.847593	-1.355731	0.101083
C	2.556371	-0.726934	-0.868165
C	1.861598	-2.848789	0.358039
H	1.521467	-3.426651	-0.512079
H	1.208052	-3.120553	1.193252
H	2.869681	-3.196699	0.614584
C	3.402342	-1.522478	-1.845627
H	3.315121	-1.140813	-2.868235
H	3.118284	-2.576337	-1.853073
H	4.467901	-1.476994	-1.584017
H	0.879760	1.216848	-2.152529
H	2.976746	1.147311	0.045564

TS3Ti-HMe
B3LYP/BS1 SCF energy: -2617.913518 a.u.
M06-L/BS2 SCF energy in solution:
-2618.079713 a.u.
M06-L/BS2 Free energy in solution:
-2617.74638 a.u.

Ti	-0.469207	0.641363	-0.571436
N	-2.225892	-0.667268	-0.073076
C	-4.461718	-2.184397	0.655712
C	-3.095492	-0.222992	0.860024
C	-2.478258	-1.856224	-0.657439
C	-3.575217	-2.642410	-0.315635
C	-4.216234	-0.945909	1.249360
H	-2.883892	0.751571	1.284046
H	-1.786679	-2.155359	-1.436151
H	-3.727575	-3.590458	-0.819874
H	-4.882785	-0.535449	1.999996
H	-5.329005	-2.773028	0.938907
C	2.132283	-1.130397	0.060202
C	3.280165	-3.682540	0.516893
C	3.470745	-1.262832	0.495494
C	1.389053	-2.315323	-0.136619
C	1.954055	-3.565411	0.092600
C	4.029039	-2.521262	0.711863
H	4.084378	-0.384936	0.658791
H	0.372792	-2.244347	-0.503649
H	1.355316	-4.456688	-0.074559
H	5.063372	-2.589911	1.037996
H	3.721917	-4.659533	0.686416
Cl	-2.002419	2.398528	-0.906147
Cl	-0.363369	-0.404367	-2.672123
C	1.199909	2.392012	0.100740
N	1.521724	0.106377	-0.159485
C	2.305174	1.335688	-0.095311
C	3.146680	1.547371	-1.360346
H	2.504141	1.624346	-2.242472
H	3.825747	0.705771	-1.516954
H	3.750057	2.457078	-1.277037
C	1.685094	3.835319	0.254316
H	2.333185	3.935888	1.131340
H	0.843855	4.523182	0.364648
H	2.252475	4.144938	-0.626886
C	0.117817	0.506772	1.356078
C	0.341166	1.872221	1.257847
C	-0.052078	-0.347991	2.562358
H	0.628755	-1.204000	2.458099
H	-1.061227	-0.766303	2.645448
H	0.201667	0.169678	3.495160
C	-0.219030	2.840922	2.276252
H	0.553607	3.487659	2.706928
H	-0.701279	2.307709	3.100257
H	-0.975905	3.488679	1.816266
H	2.955969	1.352227	0.791538
H	0.652329	2.411119	-0.894483

IM4Ti-HMe
B3LYP/BS1 SCF energy: -2617.966619 a.u.
M06-L/BS2 SCF energy in solution:
-2618.121191 a.u.
M06-L/BS2 Free energy in solution:
-2617.78486 a.u.

Ti	0.282439	-1.202613	0.524223
N	-1.855433	-0.969016	-0.071717
C	-4.593333	-0.945837	-0.639900
C	-2.335963	-1.529852	-1.200615
C	-2.737183	-0.411121	0.783973

C	-4.104964	-0.370570	0.531212
C	-3.688561	-1.543005	-1.518057
H	-1.602796	-1.986255	-1.854508
H	-2.320422	-0.004488	1.697749
H	-4.767547	0.095549	1.252180
H	-4.018755	-2.013244	-2.437851
H	-5.656085	-0.935681	-0.861828
C	0.124683	2.063466	-0.174368
C	-1.409653	4.414497	-0.163493
C	0.136446	2.924025	-1.282081
C	-0.634553	2.407978	0.949897
C	-1.399721	3.576027	0.951643
C	-0.634682	4.086813	-1.277571
H	0.752961	2.692161	-2.143004
H	-0.598243	1.765237	1.822883
H	-1.982282	3.832167	1.832082
H	-0.621779	4.741220	-2.144546
H	-2.006011	5.322230	-0.161269
Cl	0.109985	-3.487584	0.442291
Cl	0.202747	-0.600770	2.792908
C	3.061080	-0.237461	-0.040791
N	0.955819	0.879908	-0.132865
C	2.418416	1.154736	0.130343
C	2.683030	1.832400	1.468657
H	2.299017	1.229201	2.295118
H	2.226501	2.825018	1.518895
H	3.763186	1.957199	1.600233
C	4.416058	-0.157971	-0.763221
H	4.304604	0.252612	-1.773266
H	4.880325	-1.144583	-0.848912
H	5.112018	0.485703	-0.212862
C	0.863902	-0.162907	-1.166621
C	1.966677	-1.033685	-0.788880
C	0.315014	0.057493	-2.551261
H	-0.681597	0.509390	-2.547879
H	0.243414	-0.906821	-3.064332
H	0.967095	0.690533	-3.170922
C	2.381889	-2.196999	-1.664400
H	3.028122	-1.876216	-2.494025
H	1.518048	-2.709679	-2.095307
H	2.932071	-2.947614	-1.087781
H	2.771791	1.810895	-0.681254
H	3.252082	-0.669429	0.954393

IM3Ti-NF

B3LYP/BS1 SCF energy: -2484.881912 a.u.

M06-L/BS2 SCF energy in solution:
-2485.038103 a.u.

M06-L/BS2 Free energy in solution:
-2484.81409 a.u.

Ti	-0.042644	-0.664171	0.011840
N	-2.273645	0.015466	-0.122515
C	-4.872952	0.973938	-0.499874
C	-2.496139	1.286199	-0.514268
C	-3.340530	-0.779294	0.081203
C	-4.650242	-0.340923	-0.097782
C	-3.771399	1.803167	-0.710838
H	-1.612548	1.893079	-0.678169
H	-3.121187	-1.791922	0.400069
H	-5.472378	-1.026326	0.077300
H	-3.890759	2.834096	-1.025938
H	-5.882513	1.345786	-0.647207
Cl	0.027492	-0.218076	-2.245986
Cl	-0.749723	-2.491151	1.184659
C	2.666264	1.074301	-0.048653
N	1.898035	-1.160134	0.069128
C	2.896371	-0.314119	-0.204752
C	4.214844	-0.858243	-0.698042
H	4.253999	-0.815633	-1.793496
H	4.332297	-1.897336	-0.398914
H	5.052051	-0.277519	-0.306952
C	3.716630	2.021186	-0.597980
H	4.241933	1.590744	-1.452412
H	4.476194	2.290524	0.149854
H	3.264071	2.954231	-0.942414
C	0.465444	0.853508	1.107561
C	1.576522	1.607166	0.705193
C	-0.407580	1.237330	2.287654
H	-1.060683	0.410540	2.583615
H	-1.064066	2.089774	2.060926
H	0.191169	1.504502	3.167351

C	1.693867	3.061788	1.141474
H	0.941852	3.319620	1.887410
H	1.551586	3.736851	0.288208
H	2.679071	3.287540	1.563238
F	2.313185	-2.502587	0.168662

TS3Ti-NF
B3LYP/BS1 SCF energy: -2484.842133 a.u.
M06-L/BS2 SCF energy in solution:
-2485.011051 a.u.
M06-L/BS2 Free energy in solution:
-2484.79018 a.u.

Ti	0.259371	-0.639381	-0.233439
N	-1.931263	-0.103552	-0.170846
C	-4.608348	0.685584	-0.162525
C	-2.330074	0.993457	-0.846245
C	-2.856927	-0.814265	0.502259
C	-4.199843	-0.450065	0.534257
C	-3.653813	1.418569	-0.867371
H	-1.558446	1.522449	-1.394074
H	-2.496874	-1.703376	1.006831
H	-4.905236	-1.057169	1.090956
H	-3.923672	2.303355	-1.433401
H	-5.650370	0.990833	-0.161660
Cl	0.453515	-0.225482	-2.513753
Cl	-0.288980	-2.849358	-0.013459
C	1.987633	1.466682	-0.024054
N	1.794385	-0.651438	1.014567
C	2.468534	0.139899	0.096971
C	3.628829	-0.426719	-0.671938
H	3.632710	-0.049278	-1.696897
H	3.586206	-1.516262	-0.685929
H	4.575121	-0.141605	-0.196566
C	2.593918	2.447847	-0.985870
H	3.685926	2.474058	-0.909390
H	2.217637	3.458360	-0.811357
H	2.341801	2.171772	-2.018105
C	0.396523	0.568745	1.414310
C	0.914363	1.747721	0.856000
C	-0.307943	0.385727	2.721905
H	-0.282999	-0.665748	3.023505
H	-1.362509	0.677950	2.640047
H	0.144227	0.977448	3.526784
C	0.505079	3.144726	1.262685
H	1.358515	3.735631	1.616812
H	-0.237177	3.117340	2.064415
H	0.059279	3.692954	0.424328
F	2.671461	-1.801397	1.458179

IM4Ti-NF
B3LYP/BS1 SCF energy: -2484.891086 a.u.
M06-L/BS2 SCF energy in solution:
-2485.093130 a.u.
M06-L/BS2 Free energy in solution:
-2484.86702 a.u.

Ti	0.209763	-0.001040	-0.779348
N	-1.858042	0.000098	-0.061846
C	-4.551872	0.001356	0.700084
C	-2.529951	1.159839	0.122372
C	-2.530404	-1.159053	0.124665
C	-3.866292	-1.197107	0.505058
C	-3.865857	1.199155	0.502689
H	-1.972035	2.070143	-0.060697
H	-1.972770	-2.069899	-0.056573
H	-4.354440	-2.156674	0.634947
H	-4.353621	2.159175	0.630637
H	-5.597016	0.001811	0.993047
Cl	-0.033552	1.862194	-2.055425
Cl	-0.035327	-1.865135	-2.053761
C	0.890317	0.715907	1.412514
N	2.422591	-0.001294	-0.157068
C	1.821466	1.111823	0.417162
C	2.428720	2.451949	0.162702
H	1.837840	3.266767	0.587142
H	2.560089	2.631603	-0.905999
H	3.419163	2.423467	0.627653
C	0.123806	1.610068	2.340163
H	0.666705	1.717483	3.287391
H	-0.867484	1.212177	2.577028
H	0.001022	2.613214	1.923349

C	1.819522	-1.112092	0.419560	C	0.121305	-1.602984	2.344221
C	0.889036	-0.712389	1.414173	H	0.663031	-1.706975	3.292503
C	2.424154	-2.453958	0.167913	H	-0.001080	-2.607619	1.930875
H	2.557998	-2.634585	-0.900296	H	-0.870265	-1.204171	2.578433
H	1.829983	-3.267030	0.591166	F	4.066673	-0.001623	0.707607
H	3.413448	-2.428098	0.635499				
